# Regioselective electrophilic aromatic borylation as a method for synthesising sterically hindered benzothiadiazole fluorophores†

**DOI:** 10.1039/d2ra08319a

**Published:** 2023-02-16

**Authors:** Dominic Taylor, Thomas Malcomson, Adilet Zhakeyev, Georgina M. Rosair, Martin J. Paterson, Jose Marques-Hueso, Scott J. Dalgarno, Filipe Vilela

**Affiliations:** a Institute of Chemical Sciences, School of Engineering and Physical Science, Heriot-Watt University Riccarton Edinburgh EH14 4AS UK f.vilela@hw.ac.uk; b Department of Chemistry, Lancaster University Lancaster LA1 4YB UK; c Institute of Sensors, Signals and Systems, School of Engineering and Physical Science, Heriot-Watt University Riccarton Edinburgh UK

## Abstract

Regioselective stepwise phenylation of 4,7-diarylbenzo[*c*][1,2,5]thiadiazole fluorophores has been achieved through a facile one-pot, three-step synthetic strategy involving sequential borylation, hydroxydechlorination and Suzuki–Miyaura cross-coupling reactions. Crucial to the selectivity was the use of BCl_3_ to regioselectively install a boronic acid group in the *ortho*-position of only one of the diaryl groups. The subsequent introduction of *ortho*-phenyl groups through Suzuki–Miyaura cross-coupling gave rise to twisted structures with hindered intramolecular rotation, providing a structural lever with which the fluorophore absorption and emission properties could be adjusted.

## Abstract 2

A regioselective one-pot, three-step phenylation strategy has been applied to 4,7-diarylbenzo[*c*][1,2,5]thiadiazoles to restrict internal bond rotation and alter the molecules' fluorescence properties.

## Graphical abstract

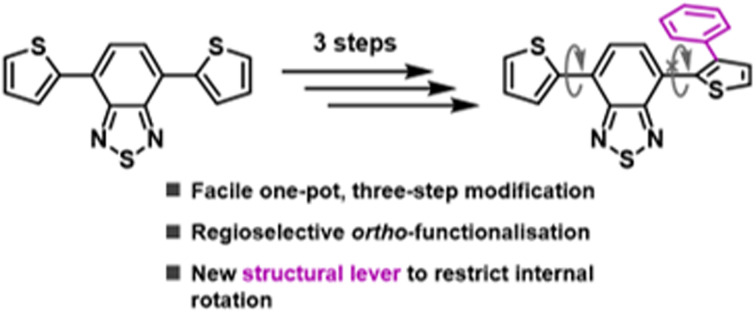

## Introduction

In recent years, organic π-conjugated electron donor–acceptor (D–A) species have garnered significant interest due to their utility in organic light harvesting and emitting applications, such as organic photovoltaics,^1^ photocatalysts,^2^ and fluorophores.^3^ The successful design of an efficient light harvesting electron D–A system is underpinned by a fundamental understanding of the structure–property relationship of a particular combination of electron donor and acceptor building blocks. Amongst the various electron acceptor building blocks, the benzo[*c*][1,2,5]thiadiazole (BTZ) group has emerged as a promising candidate, mainly due to its strongly electron accepting nature in combination with its photostability.^4–6^ Combination of the BTZ group with various donor groups has led to light harvesting applications including photocatalysis,^7–13^ photovoltaic devices,^14^ and fluorescent sensing.^5,15,16^BTZ offers a large scope for chemical derivatisation including replacing the sulfur atom with other chalcogens (O, Se and Te) or generating fused polycyclic systems, with such modifications allowing the photophysical and optoelectronic properties of a BTZ D–A system to be controlled.^17,18^

**Fig. 1:**
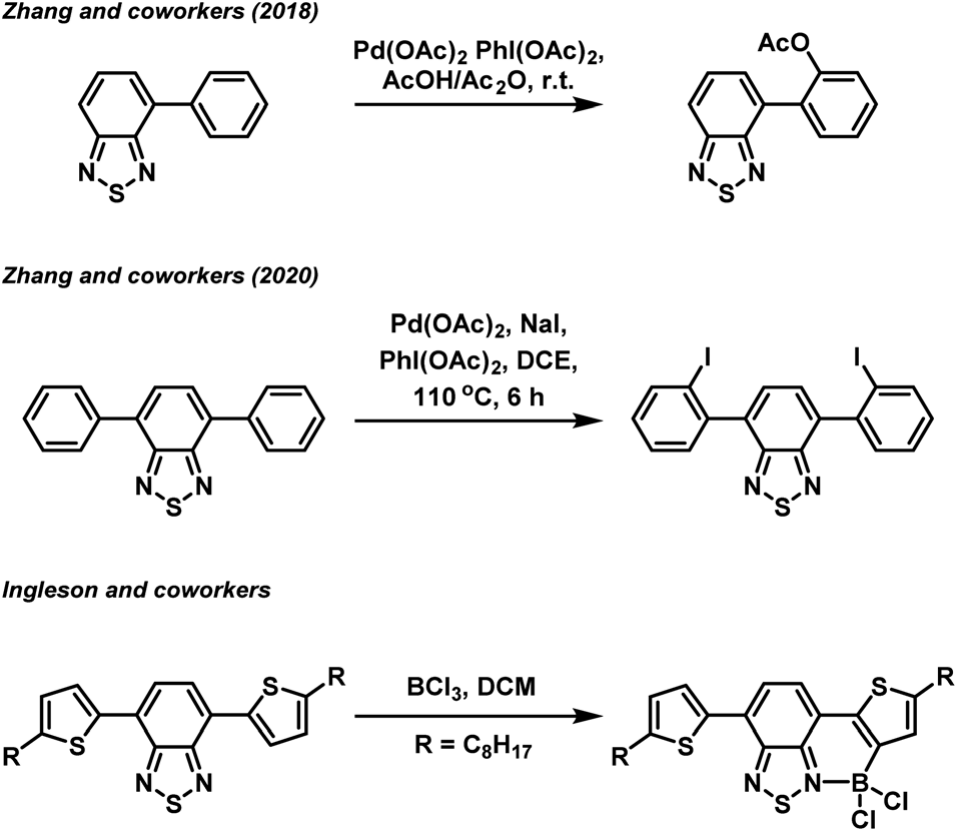
Examples of regioselective *ortho*-functionalisation of 4,7-diarylBTZs.

Beyond direction modification to the acceptor group, derivatisation of the donor aryl groups, most often located in the 4- and 7-positions (4,7-diarylBTZ), has also been explored. Recently, we have reported on the effect that changing the aryl group has on the photophysical and photoredox properties of a 4,7-diarylBTZ D–A photocatalyst system.^19^ These photocatalysts were broadly assembled through the use of Suzuki–Miyaura cross-coupling, with the variation in structure achieved by varying the combination of coupling partners. A more direct approach towards aryl group modification has been realised by Zhang and coworkers, who have reported both the palladium catalysed regioselective C–H acyloxylation and halogenation of 4-aryl and 4,7-diaryl substituted BTZs using hypervalent iodine reagents (Fig. 1).^20,21^ Similarly, regioselective C–H borylation of 4,7-diarylBTZs has been reported by Ingleson and coworkers through the use of BCl_3_.^22^ This approach resulted in the *ortho*-regioselective installation of a BCl_2_ group that engaged in the formation of a dative bond with the BTZ nitrogen atom.^23,24^ This generated a rigid, planar structure that featured extended π-conjugation and reduced the separation between the highest occupied molecular orbital (HOMO) and lowest unoccupied molecular orbital (LUMO). The susceptibility the B–Cl bond to reaction with water allowed for the formation of a boronic acid group, which Ingleson and coworkers took advantage of to synthesise thermally activated delayed fluorophores through Suzuki–Miyaura cross coupling reactions.^25^ Inspired by this approach, we herein report on the regioselective *ortho*-phenylation of 4,7-diphenylbenzo[*c*][1,2,5]thiadiazole (pH-BTZ) and 4,7-di(thiophen-2-yl)benzo[*c*][1,2,5]thiadiazole (Th-BTZ) through a similar one-pot, three step strategy. The regioselective installation of a phenyl group in the *ortho*-position introduced steric bulk that twisted the molecule around the electron donor–acceptor torsion angle, leading to hypsochromic shifts in the wavelengths of maximum absorption (*λ*_abs_) and emission (*λ*_em_), as well as changes in the lifetime of fluorescence (*τ*_f_) and photoluminescence quantum yields (PLQY).

## Results and discussion

### Fluorophore synthesis

Our previous studies conducted into BTZ compounds as fluorophores focused on the changes in light absorption and emission that could be made by derivatisation of pH-BTZ, which exhibited a *λ*_abs_ at 380 nm and a *λ*_em_ at 482 nm in chloroform.^19^ The most significant changes were observed upon substitution of the phenyl rings with heterocycles, leading to significant hypsochromic (*e.g*. 4-pyridyl groups) or bathochromic shifts (*e.g*. 2-thienyl, 2-pyrrolyl, 2-thiazoyl groups) for both absorption and emission. In particular, substitution of the phenyl groups with Th-BTZ resulted in the *λ*_abs_ and *λ*_em_ bathochromically shifting to 446 and 552 nm respectively, driven mainly by an increase in the energy of the HOMO. We were therefore interested in applying this borylation/Suzuki–Miyaura cross-coupling approach developed by Ingleson and co-workers to both pH-BTZ and Th-BTZ as representative examples of non-planar and planar D–A fluorophores respectively.

**Scheme 1:**
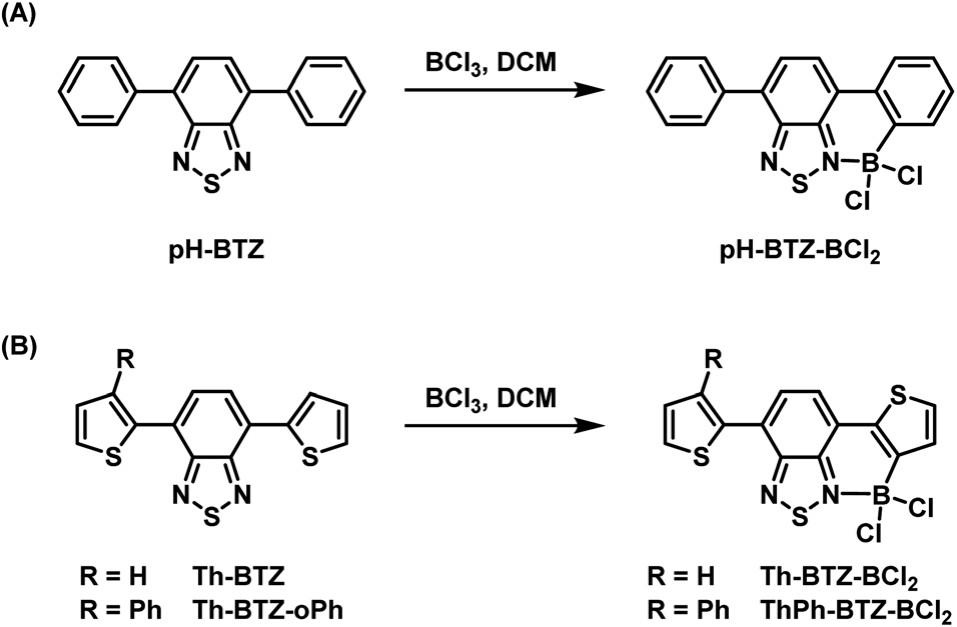
Regioselective *ortho*-borylation of 4,7-diarylBTZ containing (A) phenyl and (B) 2-thienyl groups using BCl_3_.

The addition of BCl_3_ (1 M in DCM) to a solution of pH-BTZ or Th-BTZ in DCM resulted in the formation of a dative bond with the BTZ nitrogen atom and borylation of the aryl C–H bond *ortho*-to the BTZ group (Scheme 1).^24^ After allowing these solutions to stir overnight at room temperature, all of the volatiles were removed under reduced pressure to yield pure borylated products pH-BTZ-BCl_2_ and Th-BTZ-BCl_2_ in 95 and 90% yield respectively. UV-vis absorption spectra recorded for pH-BTZ-BCl_2_ and Th-BTZ-BCl_2_ in chloroform revealed large bathochromic shifts in absorption, with maxima located at 510 and 604 nm respectively, which can be rationalised by the formation of a rigid, planar structure with extensive π-conjugation (Fig. S10 and S14†).

**Scheme 2:**
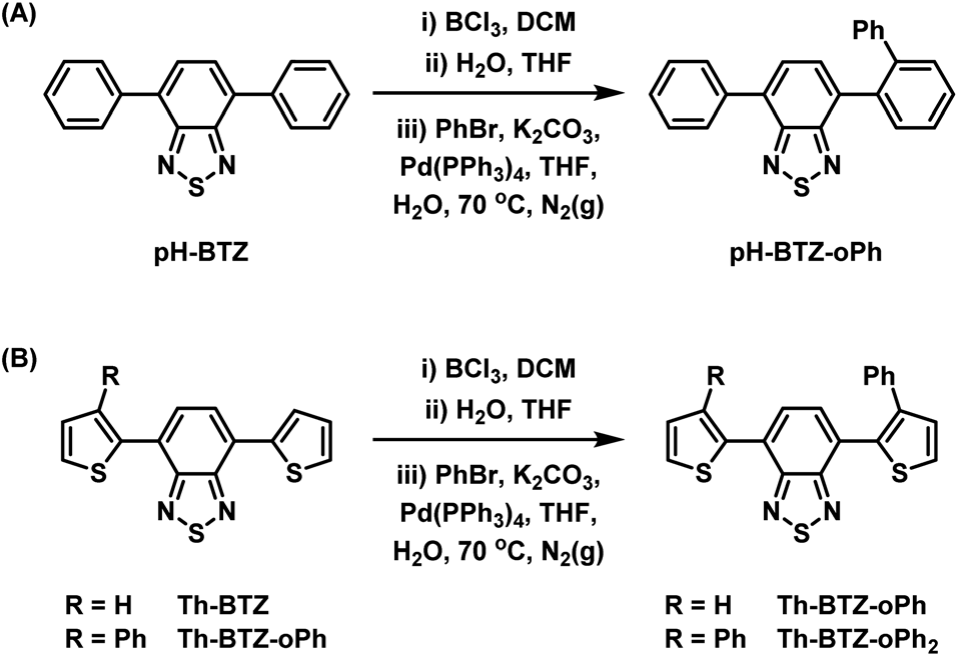
One pot, three-step approach towards regioselective *ortho*-phenylation of 4,7-diarylBTZ containing (A) phenyl and (B) 2-thienyl groups.

Following the successful isolation of pH-BTZ-BCl_2_ and Th-BTZ-BCl_2_, the one-pot, three-step phenylation strategy described in Scheme 2 was investigated. After the initial borylation step, pH-BTZ-BCl_2_ and Th-BTZ-BCl_2_ were dissolved in a THF/water mixture to elicit formation of the boronic acid which was not isolated. Following degassing these mixture with nitrogen gas, bromobenzene (2 equivalents), Pd(PPh_3_)_4_ (5 mol%) and potassium carbonate (10 equivalents) were added and the mixture heated to 70 °C. Following completion of the reaction, pH-BTZ-oPh and Th-BTZ-oPh were isolated in 55 and 57% yields, respectively.

Following the installation of the first *ortho*-phenyl group to Th-BTZ, the borylation/Suzuki–Miyaura cross coupling strategy was applied a second time to form Th-BTZ-oPh_2_ (Scheme 2B). This strategy was not extended to pH-BTZ-oPh as we have previously reported the synthesis of the symmetric molecule pH-BTZ-oPh_2_ directly *via* a single Suzuki–Miyaura cross-coupling reaction from 4,7-dibromoBTZ.^19^ However, pH-BTZ-oPh_2_ was also characterised in parallel with the fluorophores discussed in this report as an analogue of Th-BTZ-oPh_2_.

### Fluorophore UV/vis absorption and emission properties

**Fig. 2:**
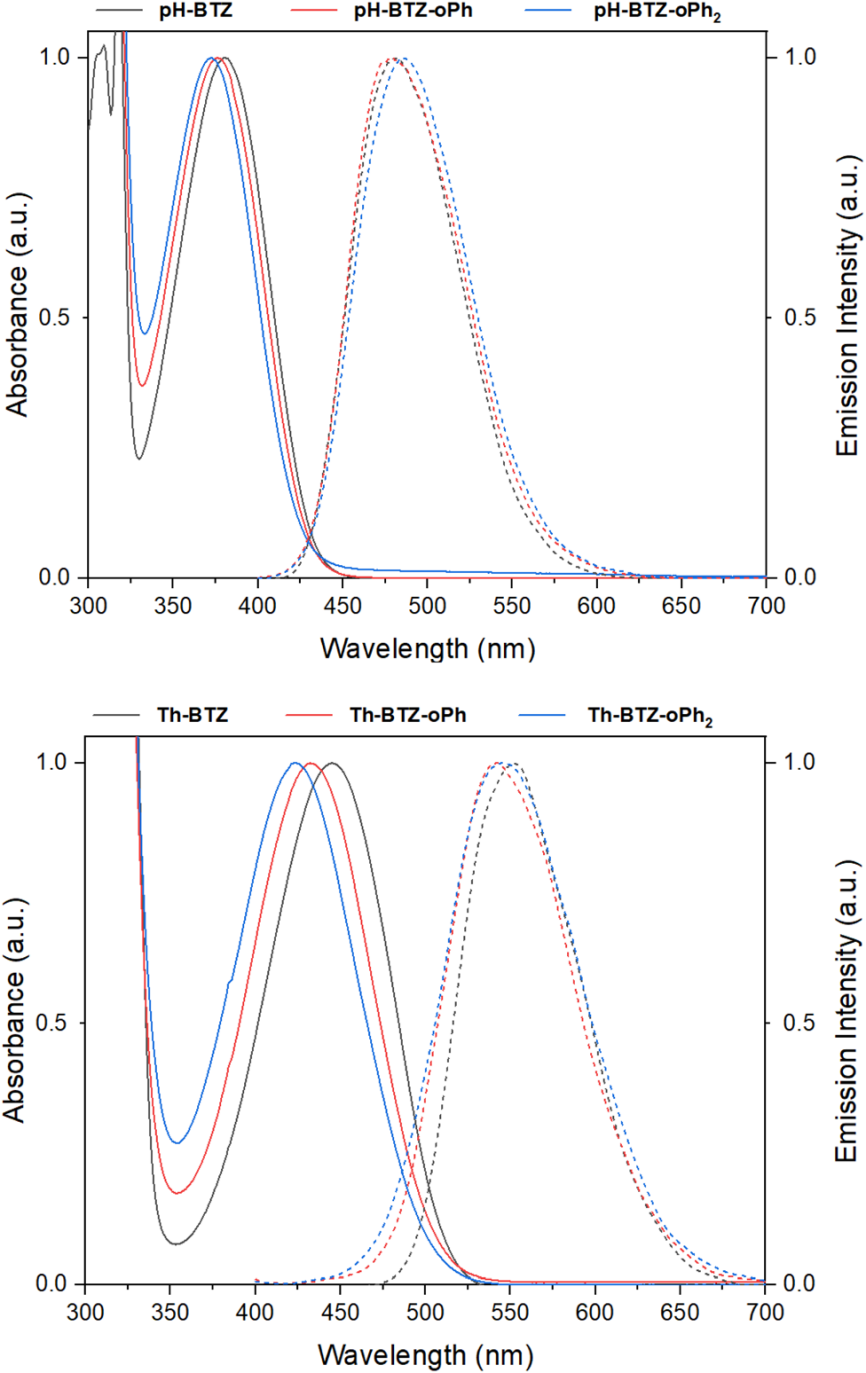
Normalised UV-vis absorption (solid lines) and emission (dashed lines) spectra for pH-BTZ (top) and Th-BTZ (bottom) derivatives in chloroform solution.

UV-vis absorption spectra were recorded for each of the fluorophores in chloroform solution. From an initial value of 380 nm, the absorption of pH-BTZ underwent modest hypsochromic shifts upon installing the *ortho*-phenyl groups (Table 1 and Fig. 2). This resulted in *λ*_abs_ of 377 and 373 nm for pH-BTZ-oPh and pH-BTZ respectively. Larger hypsochromic shifts in absorption were observed upon *ortho*-phenylation of Th-BTZ and Th-BTZ-oPh. From an initial value of 446 nm, the introduction of a single *ortho*-phenyl group resulted in a hypsochromic shift in *λ*_abs_ to 432 nm, with the addition of a second to form Th-BTZ-oPh_2_ further shifting the *λ*_abs_ to 423 nm. In both cases, the hypsochromic shift can be attributed to the sterically bulky *ortho*-phenyl groups increasing the donor–acceptor torsion angle, disrupting π-conjugation. This is consistent with previous observations made by Pathak *et al.*, with regards the effect that introducing methyl groups had on the absorption profiles of 4,7-diarylBTZs.^26^ The hypsochromic shifts we observed were more pronounced for fluorophores bearing thiophene rings, as pH-BTZ was already non-planar in solution while Th-BTZ instead preferentially adopted a planar geometry and would therefore experience comparatively greater twisting.

**Table 1:** Summary of the photophysical data for the pH-BTZ and Th-BTZ series of fluorophores

Compound	*λ*_abs_ a/nm	*λ*_em_ a ^,^ b/nm	Stokes' Shift/nm	*ε*_M_ c/× 10^3^ M^−1^ cm^−1^	PLQY^a^^,^^d^	*τ*_f_ a ^,^ e/ns	*k* f_r_/× 10^7^ s^−1^	*k*_nr_ f/× 10^7^ s^−1^
pH-BTZ	380	482	102	7.5 ± 0.1	0.864	10.01	8.63	1.36
pH-BTZ-oPh	376	480	104	6.9 ± 0.1	1.00	12.69	7.88	0.00
pH-BTZ-oPh_2_	373	486	113	6.5 ± 0.2	1.00	15.33	6.52	0.00
Th-BTZ	446	552	106	12.1 ± 0.4	0.881	13.13	6.71	0.91
Th-BTZ-oPh	432	543	111	9.3 ± 0.4	0.864	12.84	6.73	1.06
Th-BTZ-oPh_2_	423	547	124	6.8 ± 0.4	0.795	13.01	6.11	1.58

a In chloroform solution.

b Excitation at the wavelength of maximum absorbance was used for each fluorophore.

c Molar attenuation coefficient (*ε*_M_) measured as the gradient of a Beer–Lambert plot.

d Absolute PLQY.

e Fluorescence lifetime was measured by time-resolved photoluminescence spectroscopy (Fig. S17).

f *k*_r_ and *k*_nr_ were estimated from the PLQY and *τ*_f_^29^.

Fluorescence lifetimes (*τ*_f_) for each of the fluorophores involved in this study were measured using time-resolved photoluminescence spectroscopy (Table 1 and Fig. S17†). For the fluorophores based on pH-BTZ, successive *ortho*-phenylation led to an increase in *τ*_f_ from an initial value of 10.01 ns for pH-BTZ to 12.69 and 15.33 ns for pH-BTZ-oPh and pH-BTZ-oPh_2_ respectively. This is presumably due to the steric hinderance caused by introducing the *ortho*-phenyl substituents, preventing rotation around the donor–acceptor dihedral bond. This would eliminate a pathway for non-radiative relaxation of the excited state which could otherwise compete with radiative relaxation.^27^ This trend was also present in the measured photoluminescence quantum yields (PLQYs), which rose from 0.864 for pH-BTZ to 1.00 for both pH-BTZ-oPh and pH-BTZ-oPh_2_. This effect has previously been demonstrated in BTZ fluorophores by El-Zohry *et al.*, where intermolecular hydrogen bonding between carboxylic acid substituent groups suppressed intramolecular rotation about the electron donor–acceptor dihedral angle, ultimately enhancing PLQY.^28^

The trends observed for the pH-BTZ series of fluorophores were not present in the series of fluorophores based on Th-BTZ, with neither *τ*_f_ nor the PLQYs correlating with the number of phenyl substituents introduced to the electron donor 2-thienyl groups. *τ*_f_, in particular, was largely unaffected by introducing the phenyl substituents with values measured to be in the range of 12.84–13.13 ns. This would suggest that regioselective phenylation did not impact rotation of the thiophene group relative to the BTZ group to the same degree as the phenyl groups of pH-BTZ.

To gain further insight into the effect of the *ortho*-phenylation on pH-BTZ and Th-BTZ, the rate constants for radiative (*k*_r_) and non-radiative (*k*_nr_) were estimated from the PLQY and *τ*_f_ using eqn (1) and (2).^29^1
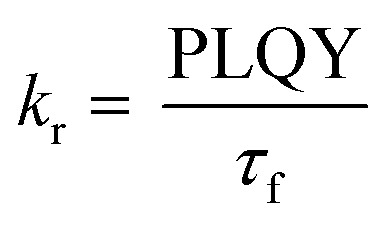
2
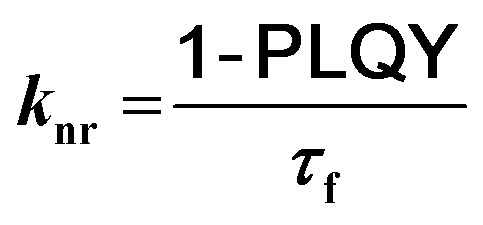


For radiative relaxation, the overall effect of *ortho*-phenylation of pH-BTZ was a modest reduction in *k*_r_ from 8.63 × 10^7^ s^−1^ to 7.88 × 10^7^ s^−1^ and 6.52 × 10^7^ s^−1^ for pH-BTZ-oPh and pH-BTZ-oPh_2_ respectively. The value of *k*_nr_ was essentially reduced to zero for both pH-BTZ-oPh and pH-BTZ-oPh_2_ by the increase in the PLQY to 1.00, highlighting the suppression of non-radiative relaxation pathways by *ortho*-phenylation. This can be directly contrasted with the effect that *ortho*-phenylation had on the Th-BTZ series of fluorophores, where *k*_r_ and *k*_nr_ showed little variation in value, although *k*_r_ still outweighed *k*_nr_.

### Computational studies

Further characterisation was conducted through density functional theory (DFT) and time dependant-DFT (TD-DFT) analysis. Investigation of the barriers to rotation of the electron donor moiety relative to the central acceptor moiety (Fig. S21 and S22†) shows a preferential deviation from planarity of 40° for the pH-BTZ structure which increases to a 60° deviation upon incorporation of the *ortho*-phenyl moiety. In contrast, Th-BTZ is shown to adopt a planar geometry with a 3 kJ mol^−1^ preference for the sulfur of the thiophene moiety to be orientated away from the central chalcogen. Planarity is lost upon incorporation of *ortho*-phenyl substituent, instead adopting a 40° deviation and inverting the orientation of the thiophene moiety, opting to place the bulkier phenyl substituent further from the central chalcogen.

Each structure containing *ortho*-phenyl groups showed a substantially lower rotational barrier when moving the phenyl group away from the central chalcogen, whereas rotating the phenyl group past the central chalcogen produced a larger energy barrier. In the case of pH-BTZ-oPh and pH-BTZ-oPh_2_, the energy barrier to rotation is *ca*. 56 kJ mol^−1^, rendering this rotation unlikely (Fig. S21†). In comparison, rotation around the BTZ-thiophene bonds in Th-BTZ-oPh and Th-BTZ-oPh_2_ presents a substantially lower energy barrier of around 27 kJ mol^−1^ (Fig. S22†). The reason for this lower energy barrier is most likely due to the pentagonal shape of the thiophene groups, which would slightly orientate any *ortho*-phenyl substituent groups they bare away from the BTZ group and reduce the steric hinderance. The lower energy barriers to internal rotation that Th-BTZ-oPh and Th-BTZ-oPh_2_ present could also explain why *τ*_f_ and PLQY are not massively changed relative to Th-BTZ, as internal rotation is not suppressed to the same degree as pH-BTZ based fluorophores.

**Fig. 3:**
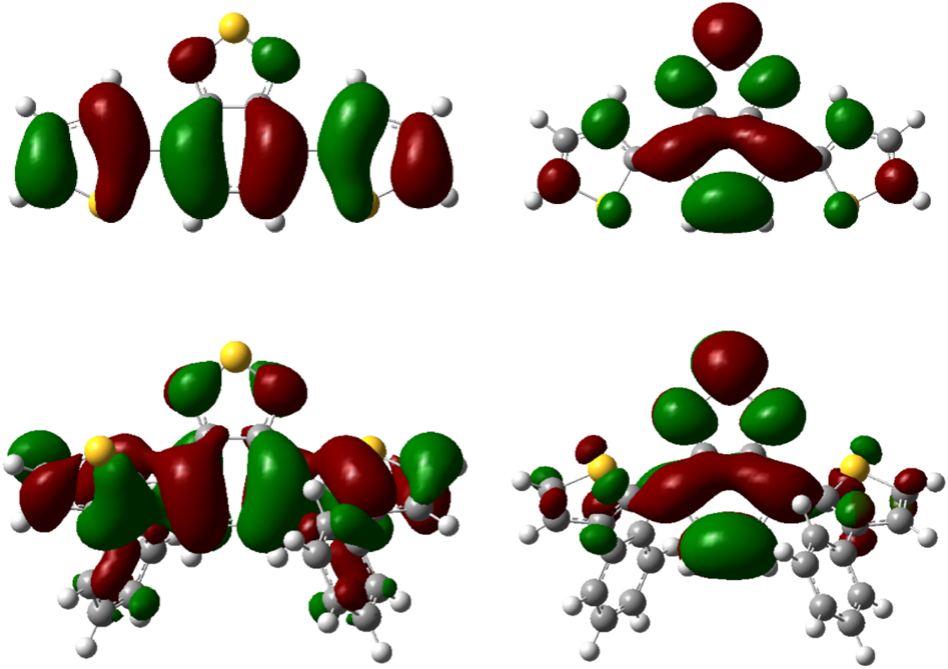
NTO representations of the hole (left) and particle (right) orbitals of the bright states responsible for the lowest energy peaks observed in the absorption spectra of Th-BTZ (top) and Th-BTZ-oPh_2_ (bottom).

Additionally, TD-DFT spectra were obtained for each spectra (Fig. S19 and S20†) which show promising alignment with those observed from experimental data (Table S2†). Natural transition orbital (NTO) analysis of the excited state providing the major contribution to the 375 nm and 425 nm peaks of pH-BTZ and Th-BTZ, respectively, showed similar character across all structures, irrespective of *ortho*-functionalisation (Tables S5–S10†). This transition, shown in Fig. 3 for the Th-BTZ structure shows a π–π* transition involving the movement of charge from the outer, electron donor groups, onto the central BTZ electron acceptor group.

### Single crystal X-ray structures

**Fig. 4:**
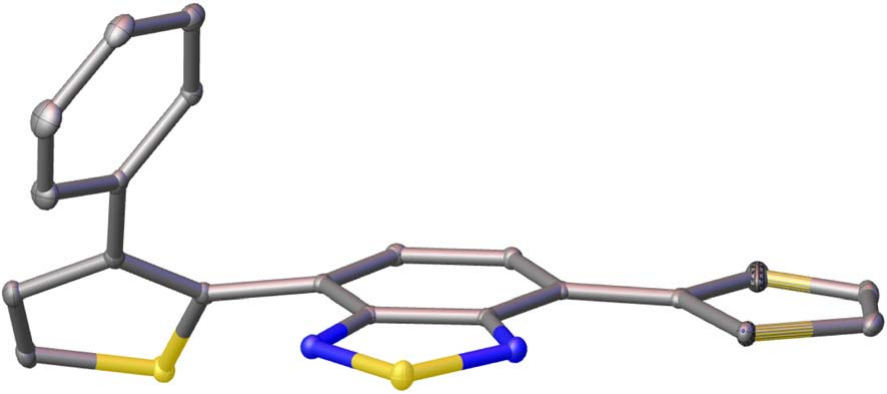
X-ray crystal structure of Th-BTZ-oPh. The atoms are shown as ellipsoids at 50% probability. H atoms omitted for clarity.

Single crystals of pH-BTZ-oPh, Th-BTZ-oPh and Th-BTZ-oPh_2_ suitable for X-ray diffraction were grown by slow evaporation from chloroform or acetone solution (Fig. S1–S8†). The structural twisting was best exemplified by the crystal structure of Th-BTZ-oPh in which the thiophene ring bearing a phenyl group was twisted to an angle of 50°, while the unmodified thiophene ring remained coplanar with the BTZ group (Fig. 4). This can be contrasted with the reported crystal structure of Th-BTZ previously reported by McCulloch and coworkers, in which both thiophene rings are coplanar with the BTZ group.^30^

## Experimental

### General methods and materials

4,7-Dibromobenzo[*c*][1,2,5]thiadiazole,^31^ palladium tetrakis(triphenylphosphine) (0) (Pd(PPh_3_)_4_),^32^ 4,7-diphenylbenzo[*c*][1,2,5]thiadiazole (pH-BTZ), 4-([1,1′-biphenyl]-2 yl)-7-phenylbenzo[*c*][1,2,5]thiadiazole (oPh-BTZ) and 4,7-di(thiophen-2-yl)benzo[*c*][1,2,5]thiadiazole (Th-BTZ) were all synthesised according to literature procedures (see ESI† for details).^19^ All commercially available reagents were used as received and purchased from Sigma-Aldrich, Fluorochem, Apollo Scientific or Fischer Scientific. DCM was dried over 4 Å activated molecular sieves before using in borylation reactions.

NMR spectra of synthetic products were recorded using a Bruker AVIII 300 MHz spectrometer using the residual solvent peak as an internal reference. All IR spectra were recorded on solid powder/crystals using a Nicolet™ iS™ 5 FTIR spectrometer. UV-vis absorption spectra for the synthesised fluorophores were obtained using a PerkinElmer Lambda 35 spectrometer in chloroform solution in quartz cuvettes with a path length of 1 cm. Emission spectra in the visible region were recorded using a PerkinElmer LS 55 fluorescence spectrometer in chloroform solution using quartz cuvettes with a path length of 1 cm. The excitation wavelength used was the wavelength of maximum absorption for each individual fluorophore. Single crystal X-ray structures were collected using a Bruker D8 venture using a Cu-Kα (*λ* = 1.5418 Å) IμS 3.0 microfocus source, using the APEX3 program suite, with the crystal kept at 100.0 K during data collection. The structures were solved using Olex2, using the SHELXT structure solution program using intrinsic phasing and refined with the SHELXL refinement package using least squares minimisation.^33–35^

### Synthesis of *ortho*-functionalised BTZ fluorophores

#### 4-([1,1′-biphenyl]-2 yl)-7-phenylbenzo[*c*][1,2,5]thiadiazole (pH-BTZ-oPh)

pH-BTZ (72 mg, 0.25 mmol) was dissolved in dry DCM (5 mL) and BCl_3_ (1 M in DCM, 1.5 mmol, 1.5 mL) added. The solution was stirred at room temperature overnight under a constant stream of nitrogen. The solvent and any excess BCl_3_ were removed under reduced pressure to yield a dark red powder. The residue was dissolved in THF (15 mL) and deionised water (5 mL) then the solution was stirred for 3 hours. K_2_CO_3_ (346 mg, 2.5 mmol) and bromobenzene (79 mg, 0.5 mmol) were added to the reaction mixture which was then bubbled with nitrogen for 30 minutes. Pd(PPh_3_)_4_ (14 mg, 0.013 mmol) was added and then the solution was heated to 70 °C for 16 hours. Following this time, the solution was cooled to room temperature then poured onto water (50 mL) and extracted with DCM (3 × 25 mL). The combined organic phases were dried over MgSO_4_ and the solvent removed under reduced pressure. The crude product was recrystallised from hot methylated spirits to yield small yellow crystals that were washed with *n*-hexane (50 mg, 55%).^1^H NMR (CDCl_3_, 300 MHz, 25.0 °C) *δ*_H_ 7.93 (m, 2H), 7.66 (m, 1H), 7.58 (d, 1H, *J* = 7.2 Hz), 7.52 (m, 5H), 7.43 (m, 1H), 7.36 (d, 1H, *J* = 7.2 Hz), 7.12 (m, 5H). ^13^C NMR (CDCl_3_, 75.5 MHz, 25.0 °C) *δ*_C_ 154.8 (C), 153.4 (C), 141.8 (C), 141.4 (C), 137.3 (C), 136.1 (C), 133.8 (C), 132.7 (C), 131.2 (CH), 130.6 (CH), 129.3 (CH), 129.2 (CH), 128.6 (CH), 128.3 (CH), 127.8 (CH), 127.6 (CH), 127.3 (CH), 126.6 (CH). IR ***ν̄*** (cm^−1^) 3050 (w, C–H str.). UV-vis (CHCl_3_) *λ*_ma*x*_ (nm) 377.

#### 4-(3-Phenylthiophen-2-yl)-7-(thiophen-2-yl)benzo[*c*][1,2,5]thiadiazole (Th-BTZ-oPh_2_)

Th-BTZ (300 mg, 1.0 mmol) was dissolved in dry DCM (5 mL) and BCl_3_ (1 M in DCM, 6 mmol, 6 mL) was added. The solution was stirred at room temperature overnight under a dynamic stream of nitrogen then the solvent and excess BCl_3_ removed under reduced pressure to yield a dark blue powder. The residue was dissolved in THF (30 mL) and deionised water (10 mL) then the solution was stirred for 3 hours. K_2_CO_3_ (1.3821 g, 2.5 mmol) and bromobenzene (314 mg, 2.0 mmol) were added to the reaction mixture which was then bubbled with nitrogen for 30 minutes. Pd(PPh_3_)_4_ (57 mg, 0.05 mmol) was added and then the solution was heated to 70 °C for 16 hours. Following this time, the solution was cooled to room temperature then poured onto water (50 mL) and extracted with DCM (3 × 25 mL). The combined organic phases were dried over MgSO_4_ and the solvent removed under reduced pressure. The crude product was then purified *via* silica gel column-chromatography using DCM : hexane 1 : 4 as the eluent to yield red crystals (217 mg, 57%). ^1^H NMR (CDCl_3_, 300 MHz, 25.0 °C) *δ*_H_ 8.13 (dd, 1H, *J* = 3.9, 1.2 Hz), 7.74 (d, 1H, *J* = 7.5 Hz), 7.57 (d, 1H, *J* = 5.2 Hz), 7.47 (dd, 1H, *J* = 5.2, 1.1 Hz), 7.45 (d, 1H, *J* = 7.5 Hz), 7.30 (m, 2H), 7.29 (d, 1H, *J* = 5.2 Hz), 7.26 (m, 3H), 7.22 (dd, 1H, *J* = 5.2, 3.8 Hz). ^13^C NMR (CDCl_3_, 75.5 MHz, 25.0 °C) *δ*_C_ 154.2 (C), 152.3 (C), 141.1 (C), 139.3 (C), 136.8 (C), 133.1, 130.6 (CH), 130.0 (CH), 128.8 (CH), 128.5 (CH), 128.0 (CH), 127.7 (CH), 127.0 (CH), 127.0 (CH), 126.5 (CH), 126.2 (C), 125.4 (C). IR ***ν̄*** (cm^−1^) 3050 (w, C–H str.). UV-vis (CHCl_3_) *λ*_ma*x*_ (nm) 432.

#### 4,7-Bis(3-phenylthiophen-2-yl)benzo[*c*][1,2,5]thiadiazole (Th-BTZ-oPh_2_)

Th-BTZ-oPh (188 mg, 0.5 mmol) was dissolved in dry DCM (5 mL) and BCl_3_ (1 M in DCM, 3 mmol, 3 mL) was added. The solution was stirred at room temperature overnight under a dynamic stream of nitrogen then the solvent and excess BCl_3_ were removed under reduced pressure to yield a dark blue powder. The residue was dissolved in THF (30 mL) and deionised water (10 mL) then the solution was stirred for 3 hours. K_2_CO_3_ (691 mg, 5.0 mmol) and bromobenzene (157 mg, 1.0 mmol) were added to the reaction mixture which was then bubbled with nitrogen for 30 minutes. Pd(PPh_3_)_4_ (29 mg, 0.025 mmol) was added and then the solution was heated to 70 °C for 16 hours. Following this time, the solution was cooled to room temperature then poured onto water (50 mL) and extracted with DCM (3 × 25 mL). The combined organic phases were dried over MgSO_4_ and the solvent removed under reduced pressure. The crude product was then purified *via* silica gel column-chromatography using DCM : hexane 1 : 4 as the eluent to yield orange-red crystals (45 mg, 20%). ^1^H NMR (CDCl_3_, 300 MHz, 25.0 °C) *δ*_H_ 7.56 (d, 2H, *J* = 5.1 Hz), 7.27 (d, 2H, *J* = 5.1 Hz), 7.26 (s, 2H), 7.23 (m, 10H). ^13^C NMR (CDCl_3_, 75.5 MHz, 25.0 °C) *δ*_C_ 153.9 (C), 141.2 (C), 136.7 (C), 132.9 (C), 130.2 (CH), 130.0 (CH), 128.8 (CH), 128.4 (CH), 127.0 (CH), 126.7 (C), 126.6 (CH). IR ***ν̄*** (cm^−1^) 3050 (w, C–H str.). UV-vis (CHCl_3_) *λ*_ma*x*_ (nm) 423.

### PLQY and fluorescence lifetime measurements

PLQYs measurements were obtained following the conventions and considerations from Jones *et al.*,^36^ using a FLS920 spectrofluorometer (Edinburgh Instruments Ltd) equipped with an extended red-sensitive photon multiplier detector (R2658P, Hamamatsu) and an integrating sphere with a 102 mm inner diameter (Yobin Yvon) were used for all PLQY measurements. For the excitation, a 450 W xenon lamp (Xe2, Edinburgh Instruments Ltd) was employed. The samples consisting of 20 μM fluorophores diluted in CHCl_3_, were contained in a square quartz cuvette with a path length of 1 cm and placed in the centre of the integrating sphere. Pure CHCl_3_ was used as reference. The excitation wavelengths were set to the peak absorption of each sample with a bandwidth set to 5 nm. The excitation and emission regions were measured with a 0.25 nm step size. The associated measurement error is 3%.

Lifetimes measurements were performed using the FLS920 spectrofluorometer, equipped with time-correlated single photon counting (TCSPC) electronics, and a pulsed light emitting diode (EPLED, from Edinburgh Instruments). The 405 nm excitation has an optical pulse approximately 1 ns long. The collection was recorded at the highest intensity emission, and the decay was fit with a single exponential.

## Conclusions

In conclusion, we have demonstrated that regioselective *ortho*-borylation on the aryl groups of BTZ fluorophores can be achieved using BCl_3_. Through subsequent hydroxydechlorination and Suzuki–Miyaura cross-coupling reactions, phenyl groups could be installed that interfered with rotation around the electron D–A bond. Evidence of this was observed both in the non-planar X-ray crystal structures and DFT calculation of the energy barrier for rotation about the electron D–A bond. In the case of fluorophores based on pH-BTZ, *ortho*-phenylation resulted in increased fluorescence lifetimes and PLQYs of up to 1.00. While *ortho*-phenylation of Th-BTZ produced larger changes in the absorption and emission spectra, the fluorescence lifetimes and PLQYs were not as significantly impacted. This facile one-pot, three-step approach presents an additional structural modification lever with which the optoelectronic and photophysical properties of BTZ electron D–A fluorophores can be controlled. In future, we envisage that this approach could be extended beyond Suzuki–Miyaura cross-coupling strategies, by taking advantage of the versatile chemistry of boronic acids to generate bespoke BTZ electron D–A systems.

## Conflicts of interest

There are no conflicts to declare.

## Supplementary Material

RA-013-D2RA08319A-s001

RA-013-D2RA08319A-s002
