# Supplementary material for: Regioselective electrophilic aromatic borylation as a method for synthesising sterically hindered benzothiadiazole fluorophores
Source: RSC Adv. 2023 Feb 16;13(9):5826–32. doi: 10.1039/d2ra08319a (PMC9951066; doi:10.1039/d2ra08319a)
Supplement: RA-013-D2RA08319A-s001 [file RA-013-D2RA08319A-s001.pdf]

Electronic Supplementary Material (ESI) for RSC Advances.
This journal is © The Royal Society of Chemistry 2023

Regioselective Electrophilic Aromatic Borylation as a Method for Synthesising Sterically Hindered Benzothiadiazole Fluorophores

Electronic Supplementary Information

Dominic Taylor,[a] Thomas Malcomson,[b] Adilet Zhakeyev,[c] Georgina M. Rosair,[a] Martin J. Paterson,[a] Jose Marques-Hueso,[c] Scott. J. Dalgarno[a,*] and Filipe Vilela[a,*]

[a]School of Engineering and Physical Sciences, Heriot-Watt University, Riccarton, Edinburgh, EH14 4AS, UK.

[b]Department of Chemistry, Lancaster University, Lancaster, LA1 4YB, UK.

[c]Institute of Sensors, Signals and Systems, School of Engineering and Physical Science, Heriot-Watt University, Riccarton, Edinburgh, EH14 4AS, UK.

E-mail: f.vilela@hw.ac.uk, s.j.dalgarno@hw.ac.uk

Table of Contents

**1.**	**General Experimental Details**	2
**2.**	**Synthetic Details**	3
2.1	Synthesis of Starting Materials	3
2.2	Synthesis of **BTZ** Fluorophores	4
**3.**	**Single Crystal X-Ray Structures**	9
**4.**	**Photophysical Properties of the Fluorophores**	13
4.1	UV-Vis Absorption and Emission Spectra	13
4.2	Molar Attenuation Coefficient Measurements	17
4.3	Absolute Photoluminescence Quantum Yield and Fluorescence Lifetime Measurements	17
4.4	Estimation of Radiative and Non-radiative Relaxation Rate Constants	19
**5.**	**Computational Studies**	20
5.1	General Information	20
5.2	Theoretical Absorption Spectra	23
5.3	Excited State Orbitals	24
5.4	Energy Barriers to Bond Rotation	30
**6.**	**Characterisation of Products**	31
**7.**	**References**	45

1. General Experimental Details

All commercially available compounds were used as received and purchased from Sigma-Aldrich, Fluorochem, Apollo Scientific or Fischer Scientific. Where stated, reactions were carried out under an atmosphere of oxygen free nitrogen purchased from BOC using oven dried glassware. Column chromatography was performed using Silicagel 60 Å 40 – 63 microns from Fluorochem. Thin layer chromatography was run using Silica gel 60 F254 TLC plates and the spots were visualised using UV illumination.

NMR spectra of synthetic products were recorded using a Bruker AVIII 300 MHz spectrometer using the residual solvent peak as an internal reference. All IR spectra were recorded on solid powder/crystals using a Nicolet™ iS™ 5 FTIR spectrometer. UV-Vis absorption spectra for the synthesised fluorophores were obtained using a Perkin-Elmer Lambda 35 spectrometer in chloroform solution in quartz cuvettes with a path length of 1 cm. Emission spectra in the visible region were recorded using a Perkin Elmer LS 55 fluorescence spectrometer in chloroform solution using quartz cuvettes with a path length of 1 cm. The excitation wavelength used was the wavelength of maximum absorption for each individual fluorophore. For comparison between different spectra, each set of data was normalised to the maximum absorbance observed in the region of 350 – 800 nm.

PLQYs measurements were obtained following the conventions and considerations from Jones et al.,[1] using a FLS920 spectrofluorometer (Edinburgh Instruments Ltd) equipped with an extended red-sensitive photon multiplier detector (R2658P, Hamamatsu) and an integrating sphere with a 102 mm inner diameter (Yobin Yvon) were used for all PLQY measurements. For the excitation, a 450 W Xenon lamp (Xe2, Edinburgh Instruments Ltd) was employed. The samples consisting of 20 μM fluorophores diluted in $CHCl_3$, were contained in a square quartz cuvette with a path length of 1 cm and placed in the centre of the integrating sphere. Pure $CHCl_3$ was used as reference. The excitation wavelengths were set to the peak absorption of each sample with a bandwidth set to 5 nm. The excitation and emission regions were measured with a 0.25 nm step size. The associated measurement error is 3%.

Lifetimes measurements were performed using the FLS920 spectrofluorometer, equipped with time-correlated single photon counting (TCSPC) electronics, and a pulsed light emitting diode (EPLED, from Edinburgh Instruments). The 405 nm excitation has an optical pulse approximately 1 ns long. The collection was recorded at the highest intensity emission, and the decay was fit with a single exponential.

Single crystal x-ray structures were collected using a Bruker D8 venture using a Cu-K_α (λ = 1.5418 Å) IμS 3.0 microfocus source, using the APEX3 program suite, with the crystal kept at 100.0 K during data collection. The structures were solved using Olex2, using the SHELXT structure solution program using Intrinsic Phasing and refined with the SHELXL refinement package using Least Squares minimisation.[2–4]

2. Synthetic Details

2.1 Synthesis of Starting Materials

Tetrakis(triphenylphosphine) palladium (0)[5]

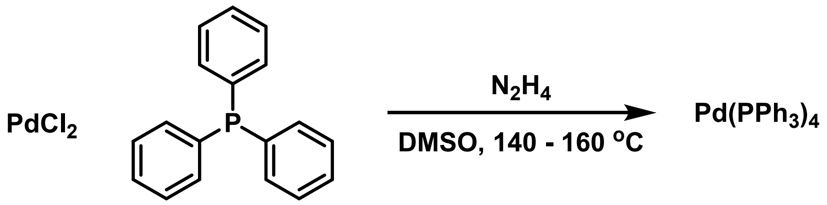

A dry, 250 mL 2-neck flask was charged with triphenylphosphine (2.63 g, 10 mmol) and palladium (II) chloride (351 mg, 2 mmol), then connected to a Schlenk line, then evacuated and back-filled with nitrogen three times. The flask was then charged with anhydrous DMSO (25 mL) and rapidly evacuated and backfilled with nitrogen three times. The mixture was then heated to 140 – 160 °C under nitrogen until an orange solution formed, then stirring continued for 15 minutes. Hydrazine hydrate (50 – 60%, 0.6 mL) was added and the mixture removed from the heat and stirring stopped, then allowed to cool to room temperature and left to sit for approximately 1 hour. During this time, yellow crystals formed that were then filtered through a sintered glass adaptor under nitrogen. The crystals were then washed with dry methanol (5 x 15 mL) and dry diethyl ether (5 x 10 mL) and allowed to dry under nitrogen and vacuum. The crystals were then transferred to a dry round bottom flask, sealed with a septum then evacuated and backfilled with nitrogen five times. The final mass of dried crystals was 2.057 g (89%).

4,7-Dibromobenzo[*c*][1,2,5]thiadiazole[6]

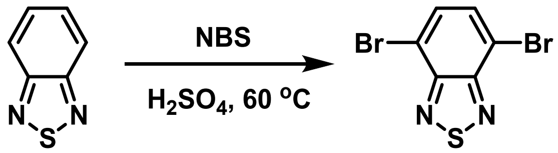

Benzo[*c*][1,2,5]thiadiazole (2.5 g, 18.4 mmol) and *N*-bromosuccinimide (NBS) (6.877 g, 38.64 mmol) were dissolved in concentrated sulfuric acid (25 mL) and the mixture heated to 60 °C for 4 hours. Following this, the reaction was allowed to cool to room temperature and then poured onto an ice-water mixture. The precipitate that formed was then filtered and washed with copious amounts of water. The final product was dried under vacuum to give an off-white powder (4.524 g, 85%). **^{1}H NMR** ($CDCl_3$, 300 MHz, 25.0 °C) δ_H 7.73 (s, 2 *H*).

2.2 Synthesis of BTZ Fluorophores

4,7-Diphenylbenzo[*c*][1,2,5]thiadiazole (pH-BTZ)[7]

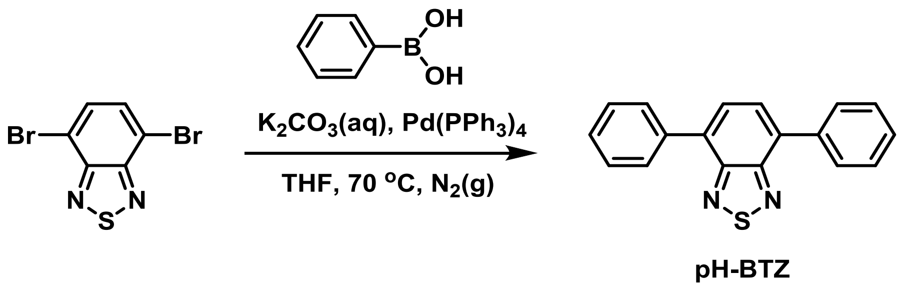

4,7-Dibromobenzo[*c*][1,2,5]thiadiazole (294 mg, 1.0 mmol), benzene boronic acid (305 mg, 2.5 mmol), potassium carbonate (276 mg, 2.0 mmol) and $Pd(PPh_3)_4$ (58 mg, 0.05 mmol) were added to a 2-neck round bottom flask. The flask was evacuated and backfilled with nitrogen gas three times then degassed THF (40 mL) and deionised water (5 mL) were added. The reaction was then heated to 70 °C for 16 hours then allowed to cool to room temperature and poured onto deionised water and extracted with DCM (3 x 25 mL). The combined organic phases were dried over $MgSO_4$ and the solvent removed under reduced pressure. The crude product was recrystallised from methylated spirits to give yellow needles (173 mg, 60%). **^{1}H NMR** ($CDCl_3$, 300 MHz, 25.0 °C) δ_H 7.97 (m, 4 *H*), 7.80 (s, 2 *H*), 7.59 (m, 4 *H*), 7.47 (m, 2 *H*). **^{13}C NMR** ($CDCl_3$, 75.5 MHz, 25.0 °C) δ_C 154.1 (C), 137.5 (C), 133.4 (C), 129.3 (CH), 128.7 (CH), 128.4 (CH), 128.2 (CH). **UV-Vis** ($CHCl_3$) λ_{max} (nm) 380. **IR** $\bar{\nu}$ (cm^{-1}) 3027 (w, C-H str.).

pH-BTZ-BCl$_2$

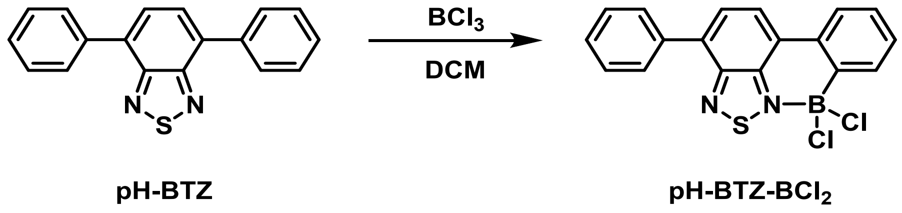

pH-BTZ (72 mg, 0.25 mmol) was dissolved in dry DCM (5 mL) then BCl_3 (1 M in DCM, 1.5 mmol, 1.5 mL) added and the solution stirred at room temperature overnight under a dynamic stream of nitrogen. Following this the solvent and excess BCl_3 were removed under reduced pressure to yield a dark red-purple powder (88 mg, 95%). **^{1}H NMR** ($CDCl_3$, 300 MHz, 25.0 °C) δ_H 8.47 (d, 1 *H*, J = 7.6 Hz), 8.08 (dd, 1 *H*, J = 7.8, 1.0 Hz), 8.05 (d, 1 *H*, J = 7.8 Hz), 8.02 (d, 1 *H*, J = 7.5 Hz), 7.94 (m, 2 *H*), 7.60 (m, 2 *H*), 7.54 (m, 2 *H*), 7.48 (m, 1 *H*). **^{11}B NMR** ($CDCl_3$, 96 MHz, 25.0 °C) δ_H 4.30 (1 *B*). **UV-Vis** ($CHCl_3$) λ_{max} (nm) 510. A ^{13}C NMR spectra of satisfactory quality could not be obtained due to the low solubility of **pH-BTZ-BCl$_2$** in common solvents.

4-([1,1'-Biphenyl]-2-yl)-7-phenylbenzo[*c*][1,2,5]thiadiazole (pH-BTZ-oPh)

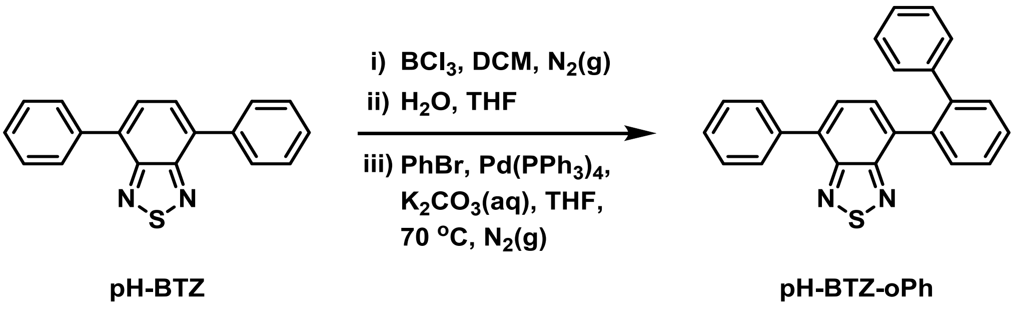

pH-BTZ (72 mg, 0.25 mmol) was dissolved in dry DCM (5 mL) and BCl_3 (1 M in DCM, 1.5 mmol, 1.5 mL) added. The solution was stirred at room temperature overnight under a constant stream of nitrogen. The solvent and any excess BCl_3 were removed under reduced pressure to yield a dark red powder. The residue was dissolved in THF (15 mL) and deionised water (5 mL) then the solution was stirred for 3 hours. K_2CO_3 (346 mg, 2.5 mmol) and bromobenzene (79 mg, 0.5 mmol) were added to the reaction mixture which was then bubbled with nitrogen for 30 minutes. $Pd(PPh_3)_4$ (14 mg, 0.013 mmol) was added and then the solution was heated to 70 °C for 16 hours. Following this time, the solution was cooled to room temperature then poured onto water (50 mL) and extracted with DCM (3 x 25 mL). The combined organic phases were dried over $MgSO_4$ and the solvent removed under reduced pressure. The crude product was recrystallised from hot methylated spirits to yield small yellow crystals that were washed with *n*-hexane (50 mg, 55%).^{1}**H NMR** ($CDCl_3$, 300 MHz, 25.0 °C) δ_H 7.93 (m, 2 *H*), 7.66 (m, 1 *H*), 7.58 (d, 1 *H*, J = 7.2 Hz), 7.52 (m, 5 *H*), 7.43 (m, 1 *H*), 7.36 (d, 1 *H*, J = 7.2 Hz), 7.12 (m, 5 *H*). ^{13}**C NMR** ($CDCl_3$, 75.5 MHz, 25.0 °C) δ_C 154.8 (C), 153.4 (C), 141.8 (C), 141.4 (C), 137.3 (C), 136.1 (C), 133.8 (C), 132.7 (C), 131.2 (CH), 130.6 (CH), 129.3 (CH), 129.2 (CH), 128.6 (CH), 128.3 (CH), 127.8 (CH), 127.6 (CH), 127.3 (CH), 126.6 (CH). **IR** $\bar{\nu}$ (cm^{-1}) 3050 (w, C-H str.). **UV-Vis** ($CHCl_3$) λ_{max} (nm) 377.

4,7-Di([1,1'-biphenyl]-2-yl)benzo[*c*][1,2,5]thiadiazole (pH-BTZ-oPh$_2$)[7]

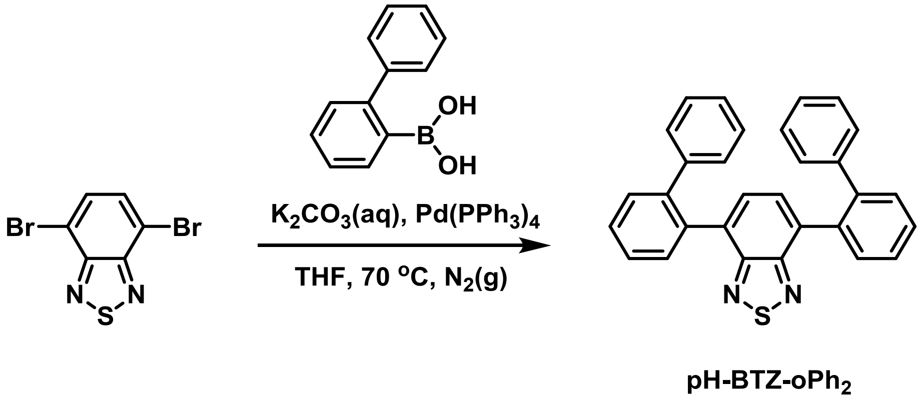

4,7-Dibromobenzo[*c*][1,2,5]thiadiazole (294 mg, 1.0 mmol), 2-biphenyl boronic acid (495 mg, 2.5 mmol), potassium carbonate (276 mg, 2.0 mmol) and $Pd(PPh_3)_4$ (58 mg, 0.05 mmol) were added to a 2-neck round bottom flask. The flask was evacuated and backfilled with nitrogen gas three times then degassed THF (40 mL) and deionised water (5 mL) were added. The reaction was then heated to 70 °C for 16 hours then allowed to cool to room temperature and poured onto deionised water and extracted with DCM (3 x 25 mL). The combined organic phases were dried over $MgSO_4$ and the solvent removed under reduced pressure. The crude product was washed with hot methylated spirits to yield a yellow powder (280 mg, 64%). **^{1}H NMR** ($CDCl_3$, 300 MHz, 25.0 °C) δ_H 7.60 (m, 2 *H*), 7.52 (m, 4 *H*), 7.48 (m, 2 *H*), 7.14 (s, 2 *H*), 7.09 (m, 10 *H*). **UV-Vis** ($CHCl_3$) λ_{max} (nm) 373. **IR** $\bar{\nu}$ (cm^{-1}) 3060 (w, C-H str.). A ^{13}C NMR spectra of satisfactory quality could not be obtained due to the low solubility of **pH-BTZ-oPh$_2$** in common solvents.

4,7-Di(thiophen-2-yl)benzo[*c*][1,2,5]thiadiazole (Th-BTZ)[7]

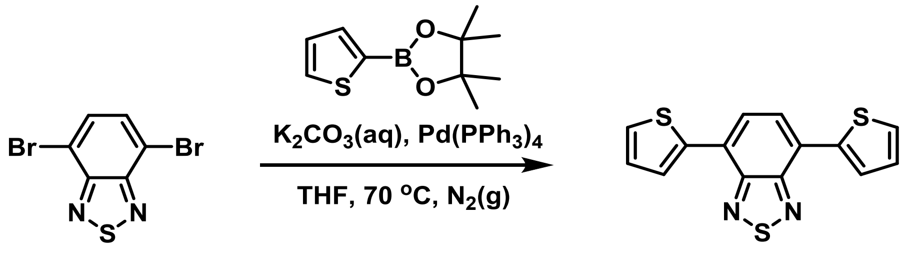

4,7-Dibromobenzo[*c*][1,2,5]thiadiazole (294 mg, 1.0 mmol), 2-thiophene boronic acid pinacol ester (525 mg, 2.5 mmol), potassium carbonate (276 mg, 2.0 mmol) and $Pd(PPh_3)_4$ (58 mg, 0.05 mmol) were added to a 2-neck round bottom flask. The flask was evacuated and backfilled with nitrogen gas three times then degassed THF (40 mL) and deionised water (5 mL) were added. The reaction was then heated to 70 °C for 16 hours then allowed to cool to room temperature and poured onto deionised water and extracted with DCM (3 x 25 mL). The combined organic phases were dried over $MgSO_4$ and the solvent removed under reduced pressure. The crude product was recrystallised from hot methylated spirits to give red needles (200 mg, 67%). **^{1}H NMR** ($CDCl_3$, 300 MHz, 25.0 °C) δ_H 8.13 (dd, J = 3.7, 1.1 Hz, 2 *H*), 7.89 (s, 2 *H*), 7.45 (dd, J = 5.1, 1.1 Hz, 2 *H*), 7.22 (dd, J = 5.1 Hz, 3.7 Hz, 2 *H*). **^{13}C NMR** ($CDCl_3$, 75.5 MHz, 25.0 °C) δ_C 152.7 (C), 139.4 (C), 128.0 (CH), 127.5 (CH), 126.8 (CH), 126.1 (C), 125.8 (CH). **UV-Vis** ($CHCl_3$) λ_{max} (nm) 446. **IR** $\bar{\nu}$ (cm^{-1}) 2990 (w, C-H str.).

Th-BTZ-BCl$_2$

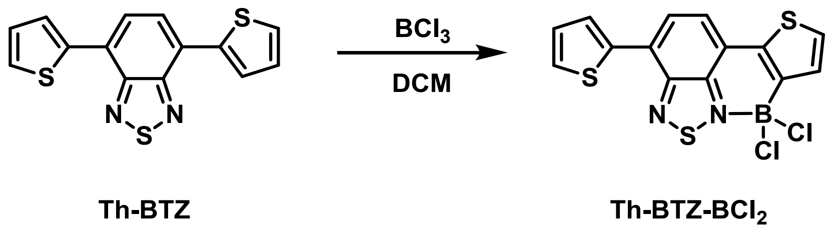

Th-BTZ (75 mg, 0.25 mmol) was dissolved in dry DCM (5 mL) then BCl_3 (1 M in DCM, 1.5 mmol, 1.5 mL) added and the solution stirred at room temperature overnight under a dynamic stream of nitrogen. Following this, the solvent and excess BCl_3 were removed under reduced pressure to yield a dark blue powder (85 mg, 90%). **^{1}H NMR** ($CDCl_3$, 300 MHz, 25.0 °C) δ_H 8.11 (dd, 1 *H*, J = 4.0, 1.1 Hz), 7.98 (d, 1 *H*, J = 7.6 Hz), 7.87 (d, 1 *H*, J = 7.6 Hz), 7.55 (dd, 1 *H*, J = 5.1, 1.1 Hz), 7.51 (m, 2 *H*), 7.25 (m, 1 *H*). **^{11}B NMR** ($CDCl_3$, 96 MHz, 25.0 °C) δ_B 3.79 (1 *B*). UV-Vis ($CHCl_3$) λ_{max} (nm) 604. A ^{13}C NMR spectra of satisfactory quality could not be obtained due to the low solubility of **Th-BTZ-BCl$_2$** in common solvents.

4-(3-phenylthiophen-2-yl)-7-(thiophen-2-yl)benzo[*c*][1,2,5]thiadiazole (Th-BTZ-oPh$_2$)

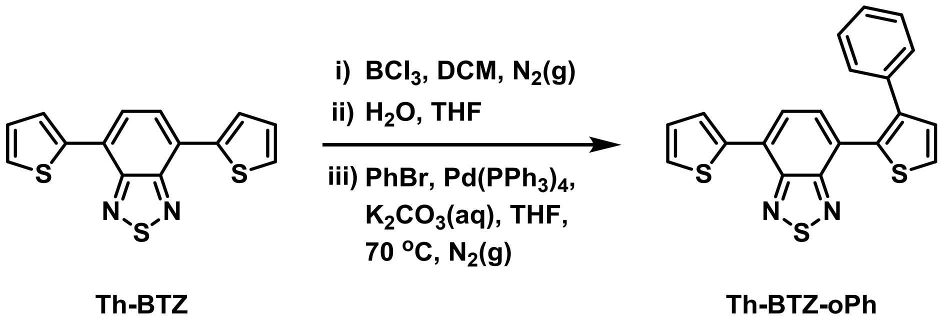

Th-BTZ (300 mg, 1.0 mmol) was dissolved in dry DCM (5 mL) and BCl_3 (1 M in DCM, 6 mmol, 6 mL) was added. The solution was stirred at room temperature overnight under a dynamic stream of nitrogen then the solvent and excess BCl_3 removed under reduced pressure to yield a dark blue powder. The residue was dissolved in THF (30 mL) and deionised water (10 mL) then the solution was stirred for 3 hours. K_2CO_3 (1.3821 g, 2.5 mmol) and bromobenzene (314 mg, 2.0 mmol) were added to the reaction mixture which was then bubbled with nitrogen for 30 minutes. $Pd(PPh_3)_4$ (57 mg, 0.05 mmol) was added and then the solution was heated to 70 °C for 16 hours. Following this time, the solution was cooled to room temperature then poured onto water (50 mL) and extracted with DCM (3 x 25 mL). The combined organic phases were dried over $MgSO_4$ and the solvent removed under reduced pressure. The crude product was then purified *via* silica gel column-chromatography using DCM:hexane 1:4 as the eluent to yield red crystals (217 mg, 57%). **^{1}H NMR** ($CDCl_3$, 300 MHz, 25.0 °C) δ_H 8.13 (dd, 1 *H*, J = 3.9, 1.2 Hz), 7.74 (d, 1 *H*, J = 7.5 Hz), 7.57 (d, 1 *H*, J = 5.2 Hz), 7.47 (dd, 1 *H*, J = 5.2, 1.1 Hz), 7.45 (d, 1 *H*, J = 7.5 Hz), 7.30 (m, 2 *H*), 7.29 (d, 1 *H*, J = 5.2 Hz), 7.26 (m, 3 *H*), 7.22 (dd, 1 *H*, J = 5.2, 3.8 Hz). **^{13}C NMR** ($CDCl_3$, 75.5

MHz, 25.0 °C) δ_C 154.2 (C), 152.3 (C), 141.1 (C), 139.3 (C), 136.8 (C), 133.1, 130.6 (CH), 130.0 (CH), 128.8 (CH), 128.5 (CH), 128.0 (CH), 127.7 (CH), 127.0 (CH), 127.0 (CH), 126.5 (CH), 126.2 (C), 125.4 (C). **UV-Vis** ($CHCl_3$) λ_{max} (nm) 432. **IR** $\bar{\nu}$ (cm^{-1}) 3050 (w, C-H str.).

4,7-Bis(3-phenylthiophen-2-yl)benzo[*c*][1,2,5]thiadiazole (Th-BTZ-oPh$_2$)

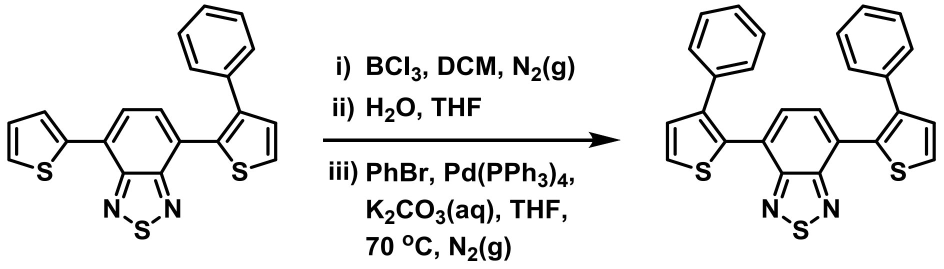

Th-BTZ-oPh **Th-BTZ-oPh$_2$**

Th-BTZ-oPh (188 mg, 0.5 mmol) was dissolved in dry DCM (5 mL) and BCl_3 (1 M in DCM, 3 mmol, 3 mL) was added. The solution was stirred at room temperature overnight under a dynamic stream of nitrogen then the solvent and excess BCl_3 were removed under reduced pressure to yield a dark blue powder. The residue was dissolved in THF (30 mL) and deionised water (10 mL) then the solution was stirred for 3 hours. K_2CO_3 (691 mg, 5.0 mmol) and bromobenzene (157 mg, 1.0 mmol) were added to the reaction mixture which was then bubbled with nitrogen for 30 minutes. $Pd(PPh_3)_4$ (29 mg, 0.025 mmol) was added and then the solution was heated to 70 °C for 16 hours. Following this time, the solution was cooled to room temperature then poured onto water (50 mL) and extracted with DCM (3 x 25 mL). The combined organic phases were dried over $MgSO_4$ and the solvent removed under reduced pressure. The crude product was then purified *via* silica gel column-chromatography using DCM:hexane 1:4 as the eluent to yield orange-red crystals (45 mg, 20%). **^{1}H NMR** ($CDCl_3$, 300 MHz, 25.0 °C) δ_H 7.56 (d, 2 *H*, J = 5.1 Hz), 7.27 (d, 2 *H*, J = 5.1 Hz), 7.26 (s, 2 *H*), 7.23 (m, 10 *H*). **^{13}C NMR** ($CDCl_3$, 75.5 MHz, 25.0 °C) δ_C 153.9 (C), 141.2 (C), 136.7 (C), 132.9 (C), 130.2 (CH), 130.0 (CH), 128.8 (CH), 128.4 (CH), 127.0 (CH), 126.7 (C), 126.6 (CH). **UV-Vis** ($CHCl_3$) λ_{max} (nm) 423. **IR** $\bar{\nu}$ (cm^{-1}) 3050 (w, C-H str.).

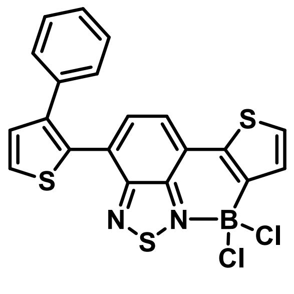

ThPh-BTZ-BCl$_2$

Although synthesised as part of a one-pot, three-step synthesised, a small amount of the intermediate **ThPh-BTZ-BCl$_2$** was withdrawn for analysis by ^{1}H and ^{11}B NMR spectroscopy. **ThPh-BTZ-BCl$_2$** was obtained as a dark-blue powder with ^{1}H NMR showing >99% conversion. **^{1}H NMR** ($CDCl_3$, 300 MHz, 25.0 °C) δ_H 7.71 (d, 1 *H*, J = 7.5 Hz), 7.62 (d, 1 *H*, J = 5.2 Hz), 7.58 (d, 1 *H*, J = 7.5 Hz), 7.49 (m, *2* H), 7.31 (d, 1 *H*, J = 5.2 Hz), 7.28 (m, 5 *H*). **^{11}B NMR** ($CDCl_3$, 96 MHz, 25.0 °C) δ_B 3.32 (1 *B*). A ^{13}C NMR spectra of satisfactory quality could not be obtained due to the low solubility of **ThPh-BTZ-BCl$_2$** in common solvents.

3. Single Crystal X-Ray Structures

Crystal data for pH-BTZ-BCl$_2$ (CCDC Deposition Number 2208644)

Crystal grown by slow evaporation from chloroform solution. Crystal data for $C_{18}H_{11}BCl_2N_2S$ (M =369.06 g/mol): monoclinic, space group P2$_1$/c (no. 14), a = 11.5839(4) Å, b = 7.0849(3) Å, c = 18.9487(8) Å, β = 91.452(3)°, V = 1554.64(11) Å^3, Z = 4, T = 101(2) K, μ(CuKα) = 5.008 mm^{-1}, $Dcalc$ = 1.577 g/cm^3, 37138 reflections measured (7.634° ≤ 2Θ ≤ 140.174°), 2992 unique (R_{int} 0.0661, R_{sigma} = 0.0335) which were used in all calculations. The final R_1 was 0.0323 (I > 2σ(I)) and wR_2 was 0.0868 (all data).

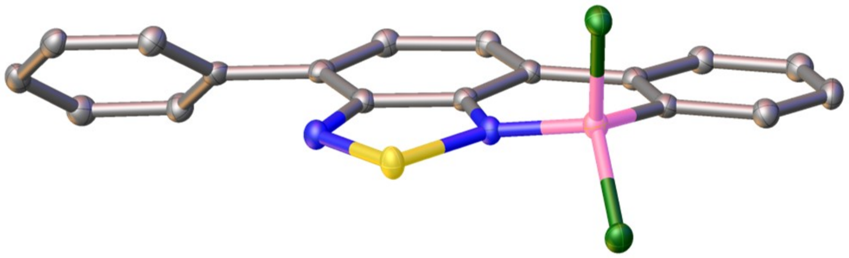

Figure S1 X-ray crystal structure of **pH-BTZ-BCl$_2$**. The atoms are shown as ellipsoids at 50% probability. H atoms omitted for clarity.

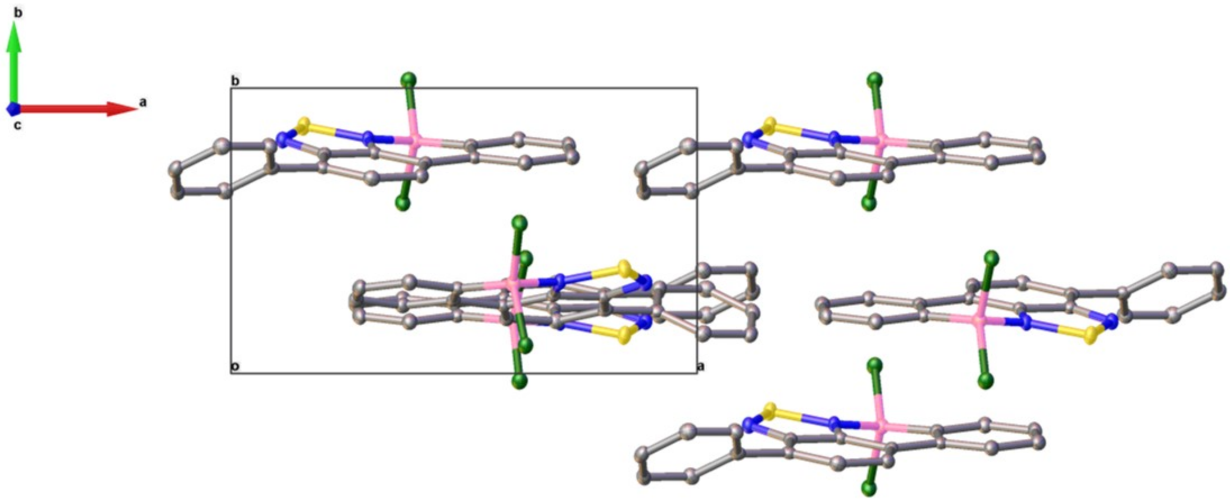

Figure S2 X-ray crystal structure of **pH-BTZ-BCl$_2$** showing the packing of molecules as viewed down the c-axis [001]. Hydrogen atoms omitted for clarity.

Crystal data for pH-BTZ-oPh (CCDC Deposition Number 2208649)

Crystal grown by slow evaporation from chloroform solution. Crystal data for $C_{24}H_{16}N_2S$ (M =364.45 g/mol): monoclinic, space group P2$_1$/n (no. 14), a = 10.4393(6) Å, b = 10.3703(5) Å, c = 17.1418(12) Å, β = 107.199(3)°, V = 1772.77(18) Å^3, Z = 4, T = 100.0 K, μ(CuKα) = 1.689 mm^{-1}, $Dcalc$ = 1.366 g/cm^3, 22077 reflections measured (10.806° ≤ 2Θ ≤ 144.302°), 3468 unique (R_{int} = 0.0329, R_{sigma} = 0.0235) which were used in all calculations. The final R_1 was 0.0351 (I > 2σ(I)) and wR_2 was 0.0883 (all data).

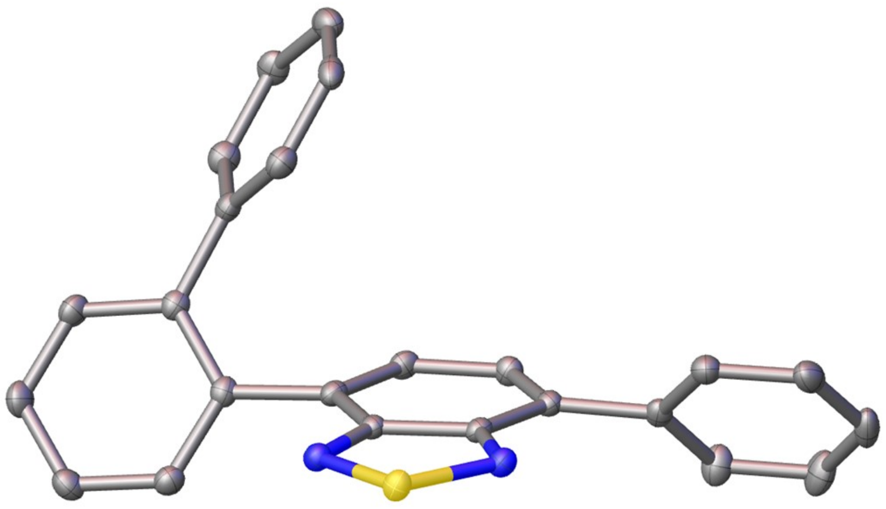

Figure S3 X-ray crystal structure of **pH-BTZ-oPh**. The atoms are shown as ellipsoids at 50% probability. H atoms omitted for clarity.

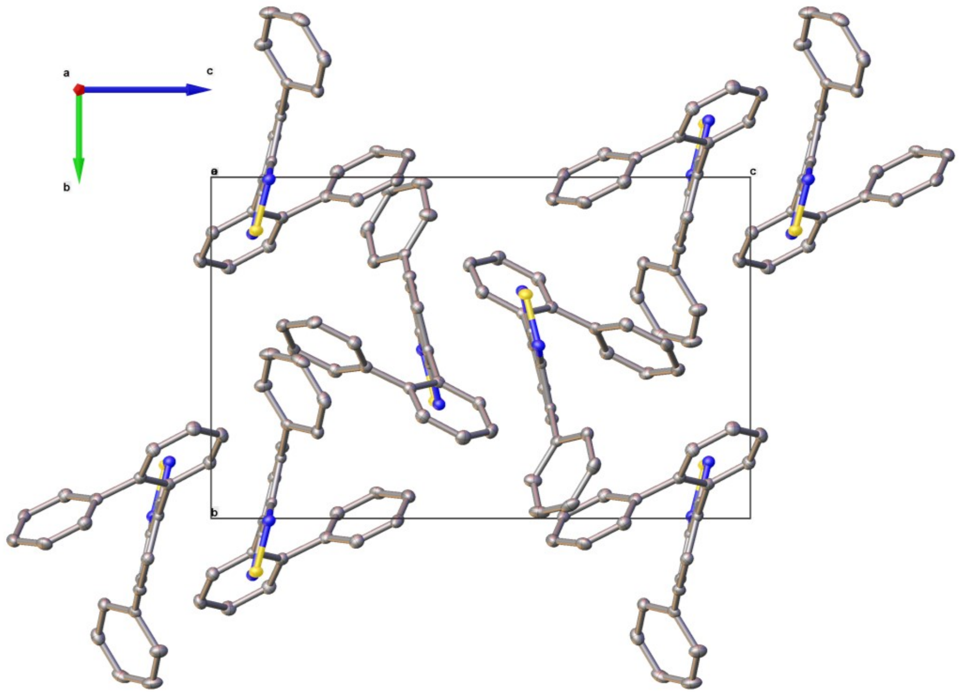

Figure S4 X-ray crystal structure of **pH-BTZ-oPh** showing the packing of molecules as viewed down the a-axis [100]. Hydrogen atoms omitted for clarity.

Crystal data for Th-BTZ-oPh (CCDC Deposition Number 2208650)

Crystal grown by slow evaporation from acetone solution. Crystal data for $C_{20}H_{12}N_2S_3$ (M =376.50 g/mol): triclinic, space group P-1 (no. 2), a = 6.87730(10) Å, b = 7.41610(10) Å, c = 16.8392(3) Å, α = 89.3640(7)°, β = 85.3723(7)°, γ = 73.5196(7)°, V = 820.81(2) Å^3, Z = 2, T = 100(2) K, μ(CuKα) = 4.157 mm^{-1}, $Dcalc$ = 1.523 g/cm^3, 18869 reflections measured (10.542° ≤ 2Θ ≤ 148.97°), 3306 unique (R_{int} = 0.0365, R_{sigma} = 0.0257) which were used in all calculations. The final R_1 was 0.0275 (I > 2σ(I)) and wR_2 was 0.0704 (all data).

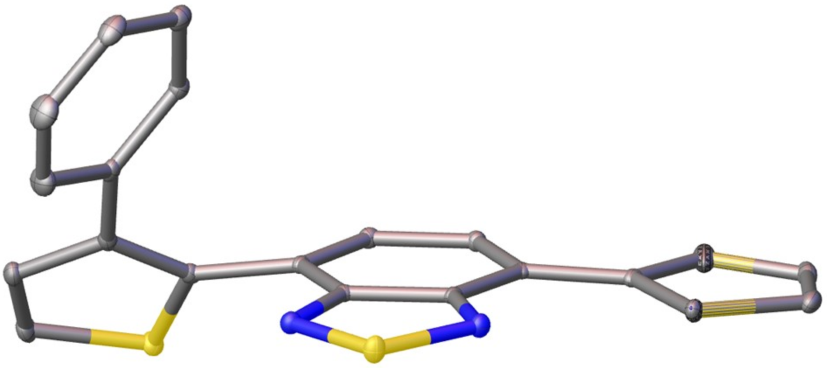

Figure S5 X-ray crystal structure of **Th-BTZ-oPh**. The atoms are shown as ellipsoids at 50% probability. H atoms omitted for clarity.

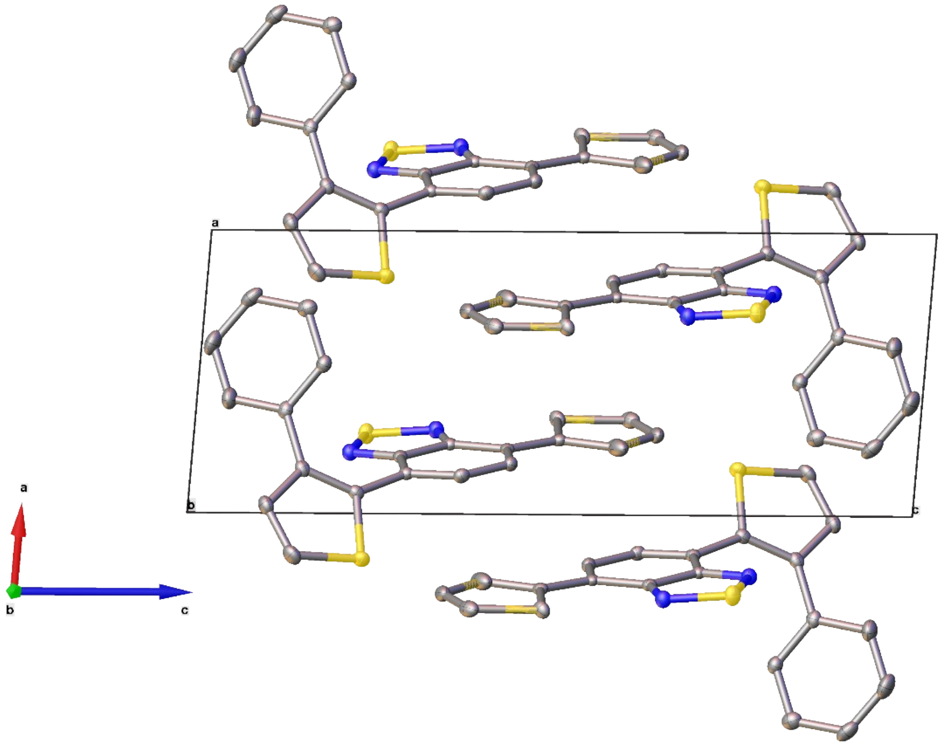

Figure S6 X-ray crystal structure of **Th-BTZ-oPh** showing the packing of molecules as viewed down the b-axis [010]. Hydrogen atoms omitted for clarity.

Crystal data for Th-BTZ-oPh$_2$ (CCDC Deposition Number 2208651)

Grown by slow evaporation from acetone solution. Crystal data for $C_{26}H_{16}N_2S_3$ (M = 452.59 g/mol): monoclinic, space group $P2_1$ (no. 4), a = 9.9159(3) Å, b = 9.8182(3) Å, c = 10.8445(3) Å, β = 101.7930(10)°, V = 1033.50(5) Å^3, Z = 2, T = 100.0 K, μ(CuKα) = 3.407 mm^{-1}, $Dcalc$ = 1.454 g/cm^3, 27584 reflections measured (11.022° ≤ 2Θ ≤ 136.47°), 3619 unique (R_{int} = 0.0326, R_{sigma} = 0.0253) which were used in all calculations. The final R_1 was 0.0224 (I > 2σ(I)) and wR_2 was 0.0586 (all data).

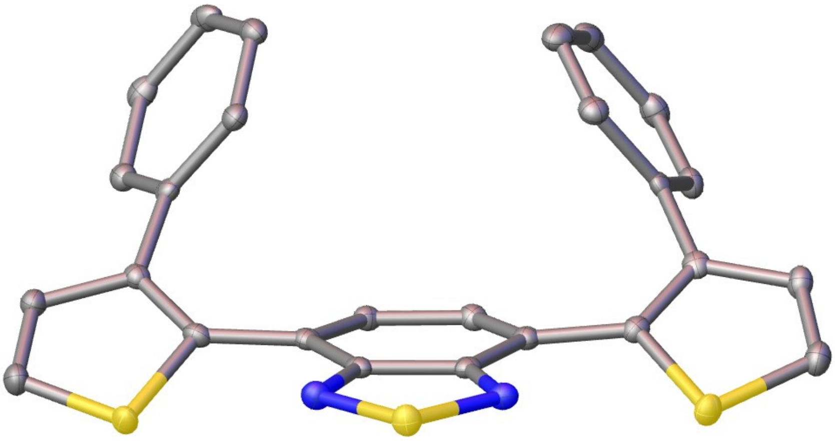

Figure S7 X-ray crystal structure of **Th-BTZ-oPh$_2$**. The atoms are shown as ellipsoids at 50% probability. H atoms omitted for clarity.

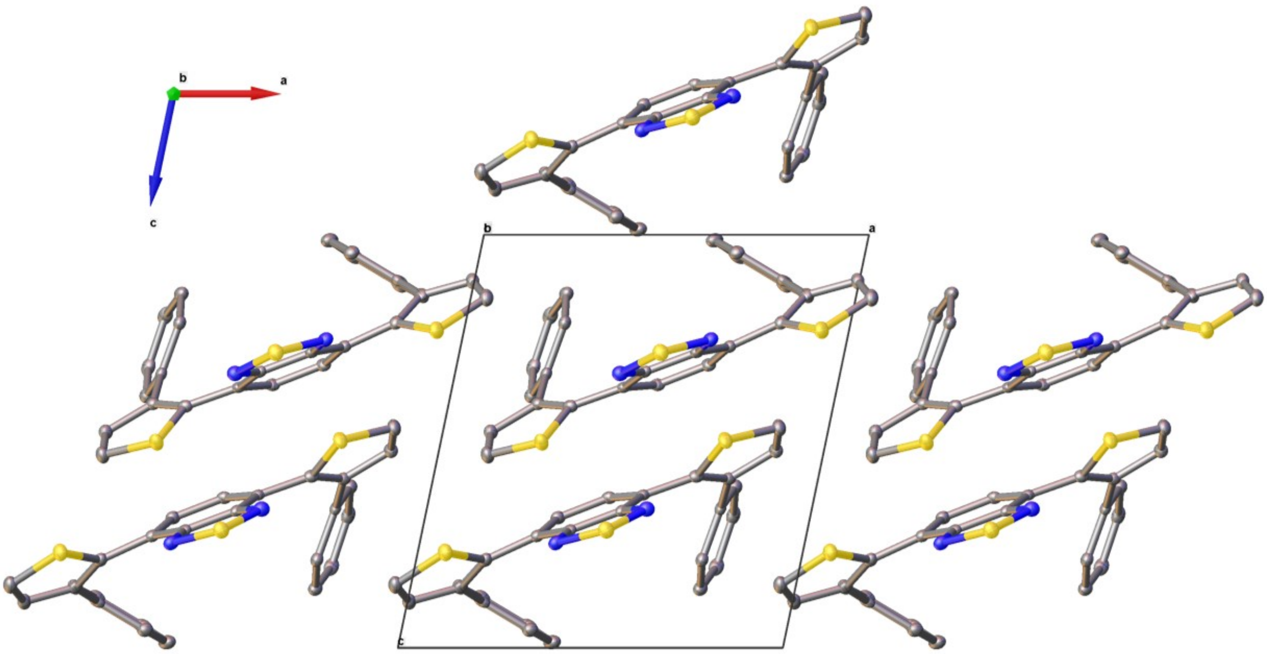

Figure S8 X-ray crystal structure of **Th-BTZ-oPh$_2$** showing the packing of molecules as viewed down the b-axis [010]. Hydrogen atoms omitted for clarity.

4. Photophysical Properties of the Fluorophores

4.1 UV-Vis Absorption and Emission Spectra

UV-Vis absorption spectra for the synthesised fluorophores were obtained using a Perkin-Elmer Lambda 35 in chloroform solution in 1 cm pathlength quartz cuvettes. For comparison between different spectra, each set of data was normalised to the maximum absorbance observed in the region of 350 – 800 nm.

Emission spectra in the visible region were recorded using a Perkin Elmer LS 55 fluorescence spectrometer in chloroform solution using quartz cuvettes. The excitation wavelength used was the wavelength of maximum absorption for each individual fluorophore. Data was collected in the region of 400 – 800 nm. Reflection of the excitation wavelengths were removed by subtracting blank spectra recorded at the same excitation wavelength.

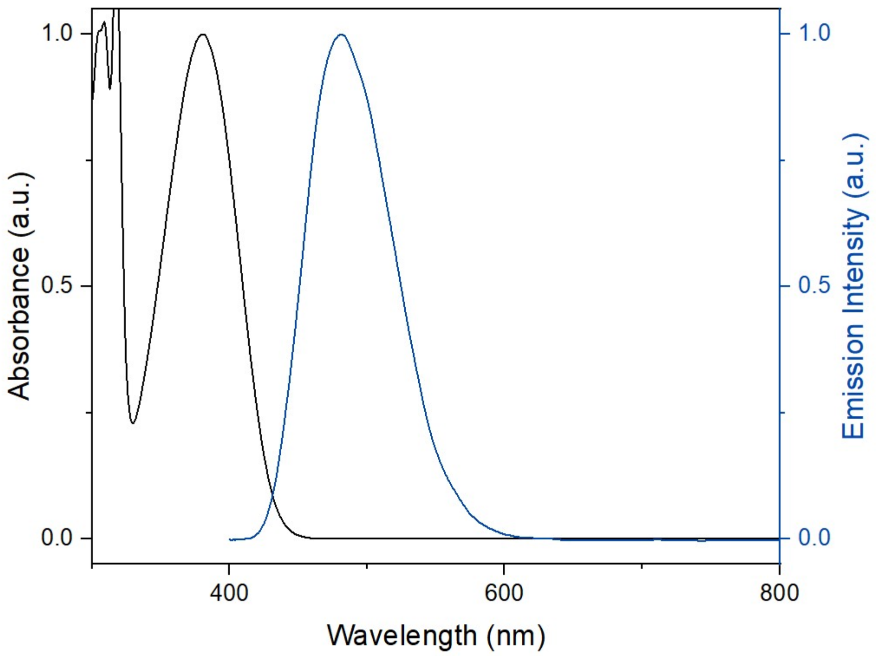

Figure S9 Normalised absorption (black) and emission spectra (blue, λ_{ex} = 380 nm) of **pH-BTZ** in $CHCl_3$.

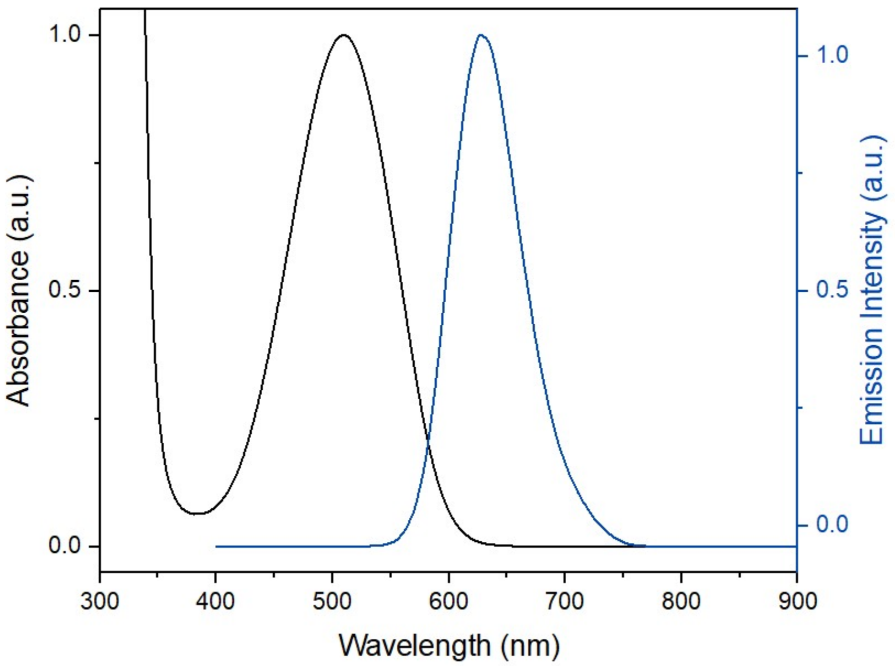

Figure S10 Normalised absorption (black) and emission spectra (blue, λ_{ex} = 510 nm) of **pH-BTZ-BCl$_2$** in $CHCl_3$.

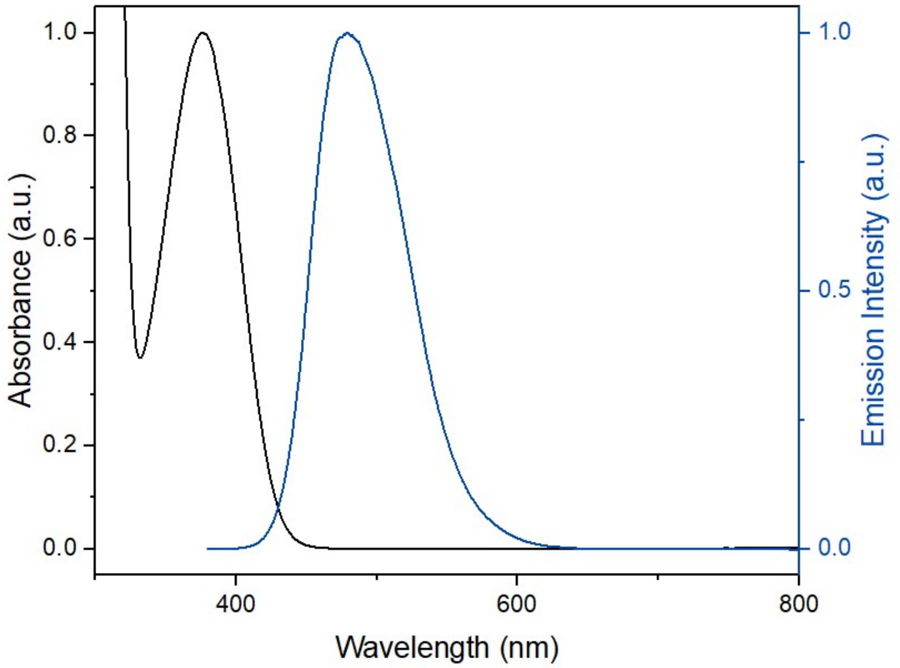

Figure S11 Normalised absorption (black) and emission spectra (blue, λ_{ex} = 377 nm) of **pH-BTZ-oPh** in $CHCl_3$.

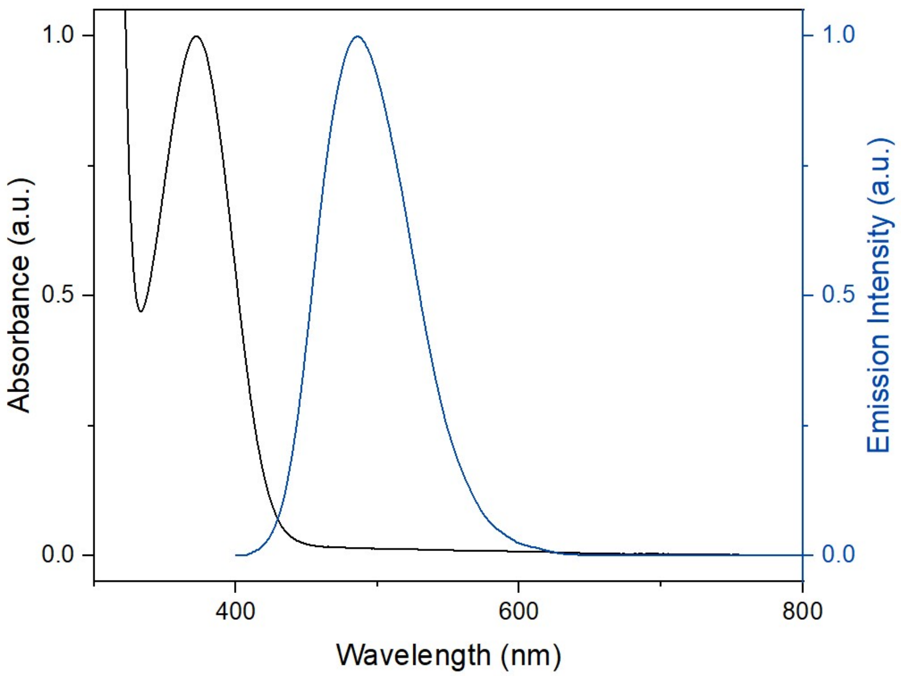

Figure S12 Normalised absorption (black) and emission spectra (blue, λ_{ex} = 373 nm) of **pH-BTZ-oPh$_2$** in $CHCl_3$.

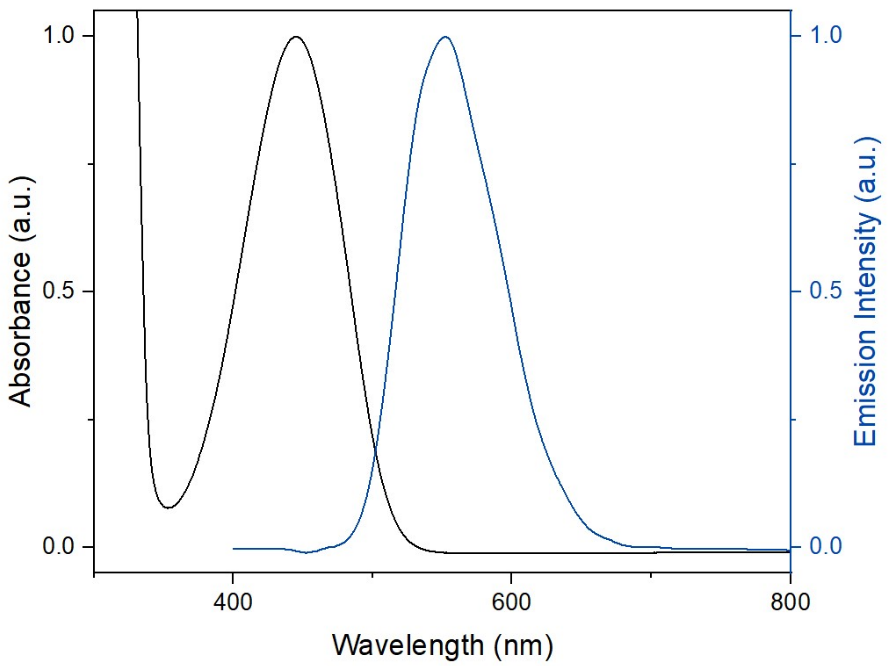

Figure S13 Normalised absorption (black) and emission spectra (blue, λ_{ex} = 446 nm) of **Th-BTZ** in $CHCl_3$.

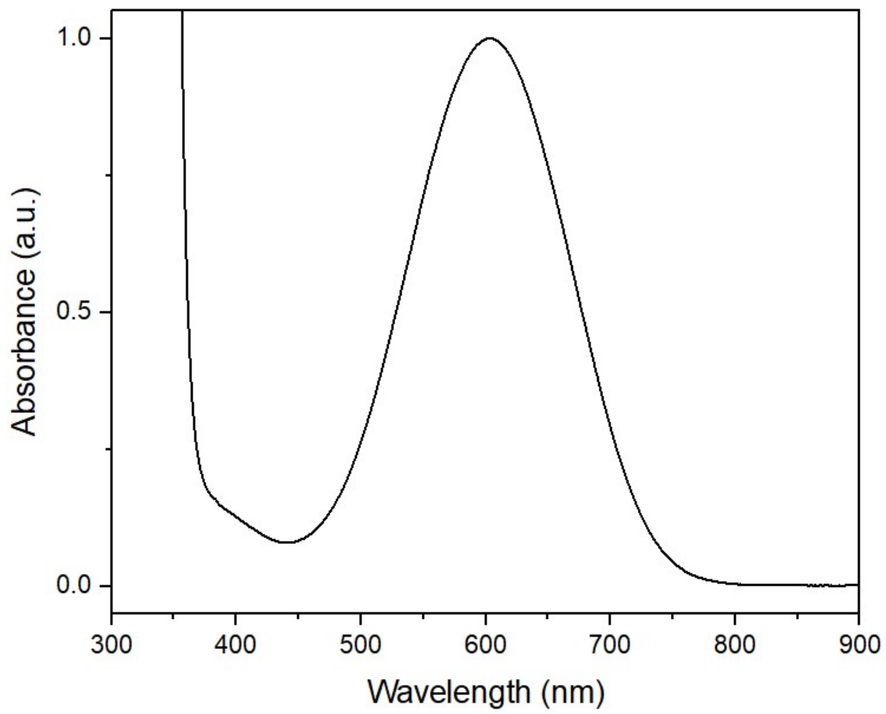

Figure S14 Normalised absorption of **Th-BTZ-BCl$_2$** in $CHCl_3$. No visible emission was detected.

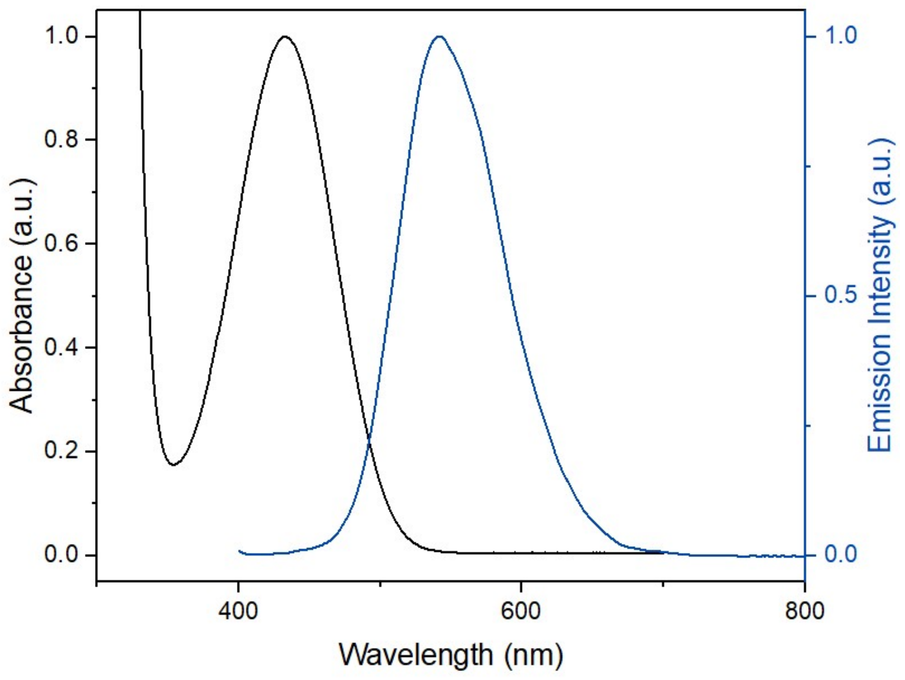

Figure S15 Normalised absorption (black) and emission spectra (blue, λ_{ex} = 432 nm) of **Th-BTZ-oPh** in $CHCl_3$.

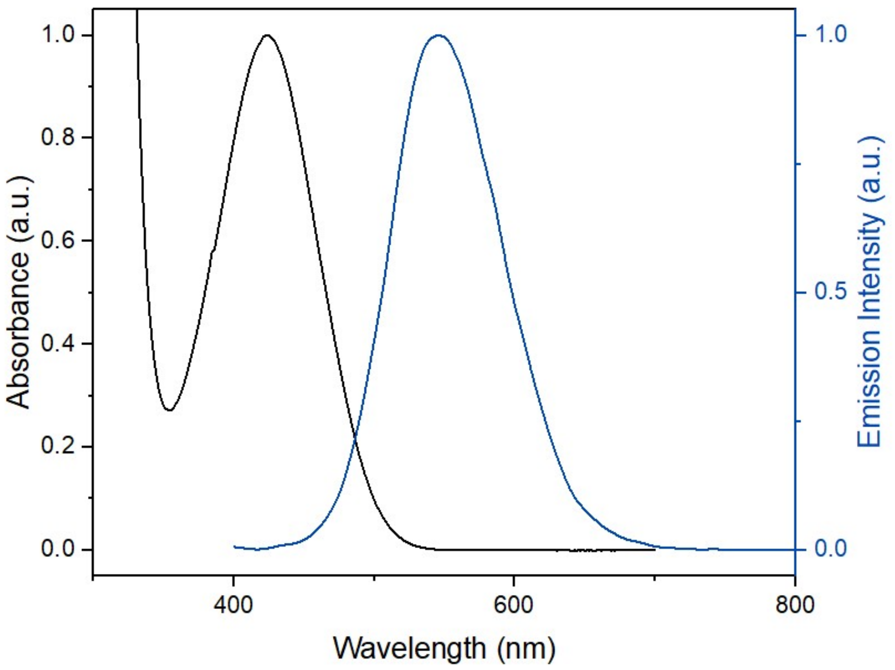

Figure S16 Normalised absorption (black) and emission spectra (blue, λ_{ex} = 423 nm) of **Th-BTZ-oPh$_2$** in $CHCl_3$.

4.2 Molar Attenuation Coefficient Measurements

Molar attenuation coefficients (ε_M) of each fluorophore were measured by preparing stock solutions of each fluorophore in chloroform with concentrations in the range of 0 - 20 µM. The absorbance of each of these solutions was then measured. Plotting absorbance against concentration gave a Beer-Lambert plot, with the attenuation coefficient equal to the gradient. The error quoted in the attenuation coefficient was taken as the error in the line fitting.

4.3 Absolute Photoluminescence Quantum Yield and Fluorescence Lifetime Measurements

PLQYs measurements were obtained using a FLS920 spectrofluorometer, equipped with an extended red-sensitive photon multiplier detector and an integrating sphere with a 102 mm inner diameter. PLQY is defined as the ratio of the number of emitted photons (L_{Sample}) to the number of absorbed photons, where the intensity of unabsorbed excitation light by a reference or by a sample is represented by $E_{Reference}$ and E_{Sample} respectively, as shown by equation 1:

$$PLQY = \frac{\#photons\ emitted}{\#photons\ absorbed} = \frac{L_{Sample}}{E_{Reference} - E_{Sample}} \tag{1}$$

The samples consisting of 20 μM fluorophores diluted in $CHCl_3$, were contained in a square quartz cuvette. Pure $CHCl_3$ was used as reference. Each sample was excited at the wavelength corresponding to absorption maxima, shown in Table S1, using a 450 W Xenon lamp.

Lifetimes measurements were performed using the FLS920 spectrofluorometer, equipped with time-correlated single photon counting (TCSPC) electronics, and a 405 nm pulsed light emitting diode (EPLED) as light source. The optical pulse is approximately 1 ns long (Figure S17). The collection was recorded at the highest intensity emission. The decay was fit with a single exponential, following the equation:

$$I = I_0 \cdot e^{\frac{-t}{\tau}} \quad (2)$$

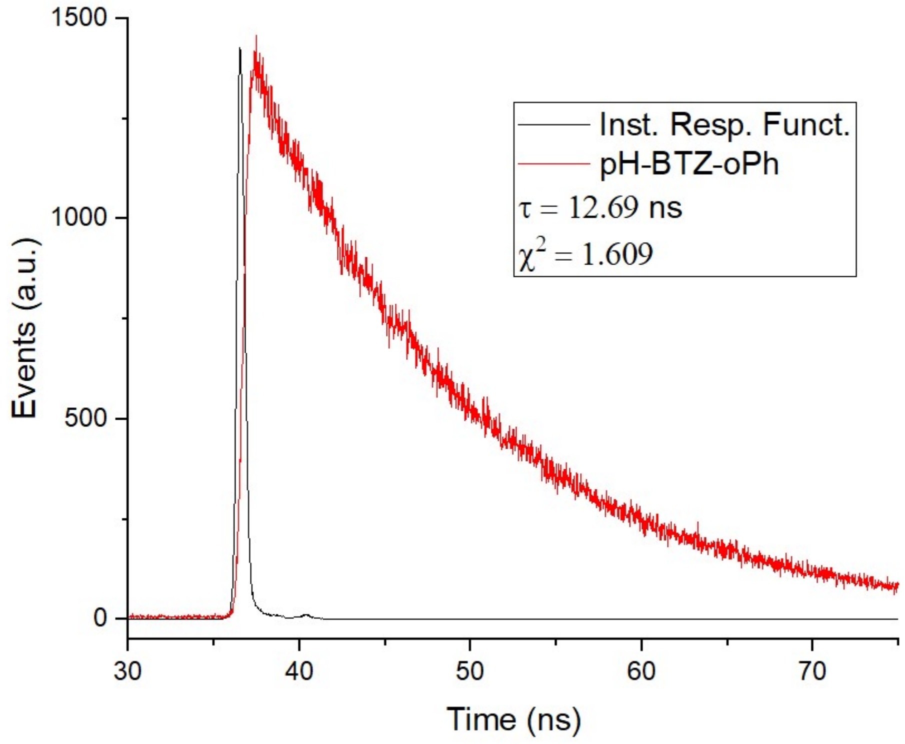

Figure S17 Photoluminescence spectra of **pH-BTZ-oPh**.

4.4 Estimation of Radiative and Non-radiative Relaxation Rate Constants

The rate constants for radiative relaxation (k_r) and non-radiative relaxation (k_{nr}) were estimated from PLQY and τ_f using the relationships expressed in equations 3 and 4.[8]

$$k_r = \frac{PLQY}{\tau_f} \tag{3}$$

$$k_{nr} = \frac{1 - PLQY}{\tau_f} \tag{4}$$

Table S1 Summary of the photophysical data for the **pH-BTZ** and **Th-BTZ** series of fluorophores. [a]In chloroform solution. [b]Excitation wavelength was the wavelength of maximum absorbance for each fluorophore. [c]Molar attenuation coefficient (ϵ_M) was measured as the gradient of a Beer-Lambert plot. [d]Absolute PLQY. [e]Fluorescence lifetime was measured by time-resolved photoluminescence spectroscopy (Figure S17). [f]χ^2 values for each photoluminescence spectra. [g]Estimated using equations 3 and 4.

Compound	λ_{abs}/ nm[a]	λ_{em}/ nm[a,b]	Stokes' Shift/nm	ε_M / x 10^3 M^{-1} cm^{-1} [c]	PLQY [a,d]	τ_f/ns[a,e]	χ^2	k_r/x 10^7 s^{-1} [g]	k_{nr}/x 10^7 s^{-1} [g]
**pH-BTZ**	380	482	102	7.5 ± 0.1	0.864	10.01	1.331	8.63	1.36
**pH-BTZ-oPh**	376	480	104	6.9 ± 0.1	1.00	12.69	1.609	7.88	0.00
**pH-BTZ-oPh$_2$**	373	486	113	6.5 ± 0.2	1.00	15.33	1.768	6.52	0.00
**Th-BTZ**	446	552	106	12.1 ± 0.4	0.881	13.13	1.927	6.71	0.91
**Th-BTZ-oPh**	432	543	111	9.3 ± 0.4	0.864	12.84	1.992	6.73	1.06
**Th-BTZ-oPh$_2$**	423	547	124	6.8 ± 0.4	0.795	13.01	2.101	6.11	1.58

5. Computational Studies

5.1 General Information

All calculations were carried out utilising the Gaussian16 software,[9] and visualised using GaussView6.[10] Geometry optimisations were conducted utilising the B3LYP[11-15]/cc-pVTZ[16] model chemistry incorporating the SMD solvent model to model a dichloromethane solvent environment; structural minima were verified through the presence of only positive curvature upon vibrational frequency analysis.[17] TD-DFT calculations of the absorption spectra, along with NTO analysis, were conducted utilising the CAM-B3LYP[18]//cc-pVTZ model chemistry, again incorporating the SMD solvent model. CAM-B3LYP was selected at the functional of choice due to both its agreement with experimentally derived spectra within this work, and its strong performance in similar systems.[19-23] States showing significant contribution to the absorption peak, selected by an oscillator strength above 0.4, were selected for further analysis. Natural transition orbitals (NTOs) were calculated for each of these states, with the relative particle-hole orbitals relating to these transitions are shown in Tables S5-S10.

Table S2 Comparison of the theoretical and experimental absorption maxima for the synthesised **BTZ**s.

**Compound**	**Experimental λ_{abs}/nm**	**Theoretical λ_{abs}/nm**
**pH-BTZ**	380	369
**pH-BTZ-oPh**	376	362
**pH-BTZ-oPh$_2$**	373	344
**Th-BTZ**	446	451
**Th-BTZ-oPh**	432	413
**Th-BTZ-oPh$_2$**	423	397

Table S3 Comparison between the optimised computational geometry and literature crystal structures for **pH-BTZ** and **Th-BTZ**. Experimental data from reported X-ray crystal structures.[24,25] Theoretical data from previous computational studies conducted on **BTZ** photocatalysts.[7]

**Geometric Feature**	**pH-BTZ**		**Th-BTZ**	
	Theoretical	Experimental	Theoretical	Experimental
**Bond Lengths/Å**				
S-N (1)	1.62554	1.6273	1.6249	1.6096
N-C (2)	1.33713	1.3594	1.33506	1.3462
C-C (3)	1.45205	1.4514	1.45479	1.4442
Acceptor-Donor C-C (4)	1.47843	1.4994	1.45532	1.4330
**Angles/°**				
N-S-N (5)	100.2613	101.45	99.87949	101.509
S-N-C (6)	106.9086	106.32	107.3116	106.3613
Acceptor-Donor C-C-C (7)	123.0716	123.03	123.0282	123.04
**Dihedral Angles/°**				
Acceptor-Donor C-C-C-C (8)	-141.352	-142.53	-	-
Acceptor-Donor C-C-C-S (9)	-	-	-179.97	-175.711

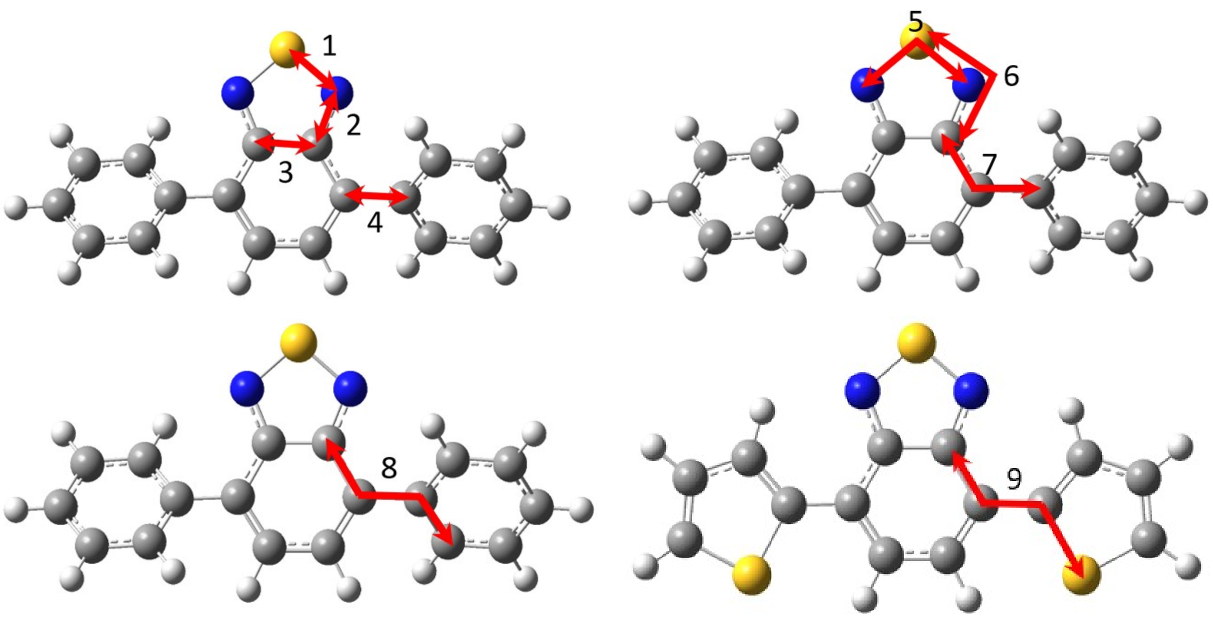

Figure S18 Naming scheme for geometric features compared in Table S3. Colour legend: carbon (grey), hydrogen (white), nitrogen (blue) and sulfur (yellow).

Table S4 Comparison between the optimised computational geometry and crystal structures for **pH-BTZ-oPh**, **Th-BTZ-oPh** and **Th-BTZ-oPh$_2$**. Experimental data from the X-ray crystal structures is shown in section 3.

Geometric Feature	pH-BTZ-oPh		Th-BTZ-oPh		Th-BTZ-oPh$_2$	
	Theoretical	Experimental	Theoretical	Experimental	Theoretical	Experimental
**Bond Lengths/Å**						
S-N	1.6253	1.6173	1.6248	1.6134	1.6255	1.616
N-C	1.3378	1.3518	1.3361	1.3478	1.3362	1.345
C-C	1.4466	1.431	1.4472	1.4277	1.4456	1.434
Acceptor-Donor C-C	1.4796	1.4911	1.458	1.4718	1.4676	1.468
Donor-oPh C-C	1.4866	1.491	1.467	1.4767	1.4676	1.485
**Angles/^{o}**						
N-S-N	100.41	101.59	100.25	101.28	100.36	100.83
S-N-C	106.59	106.17	106.72	106.22	106.66	106.79
Acceptor-Donor C-C-C	122.79	122.29	122.38	121.13	122.6	122.82
Donor-oPh C-C-C	121.76	122.55	122.73	125.60	122.6	127.8
**Dihedral Angles/^{o}**						
Acceptor-Donor C-C-C-C/S	-137.87	-128.38	-156.78	-125.82	-52.8	-33.6
Donor-oPh C-C-C-C	122.48	126.33	-53.29	-17.63	-52.82	-43.4

5.2 Theoretical Absorption Spectra

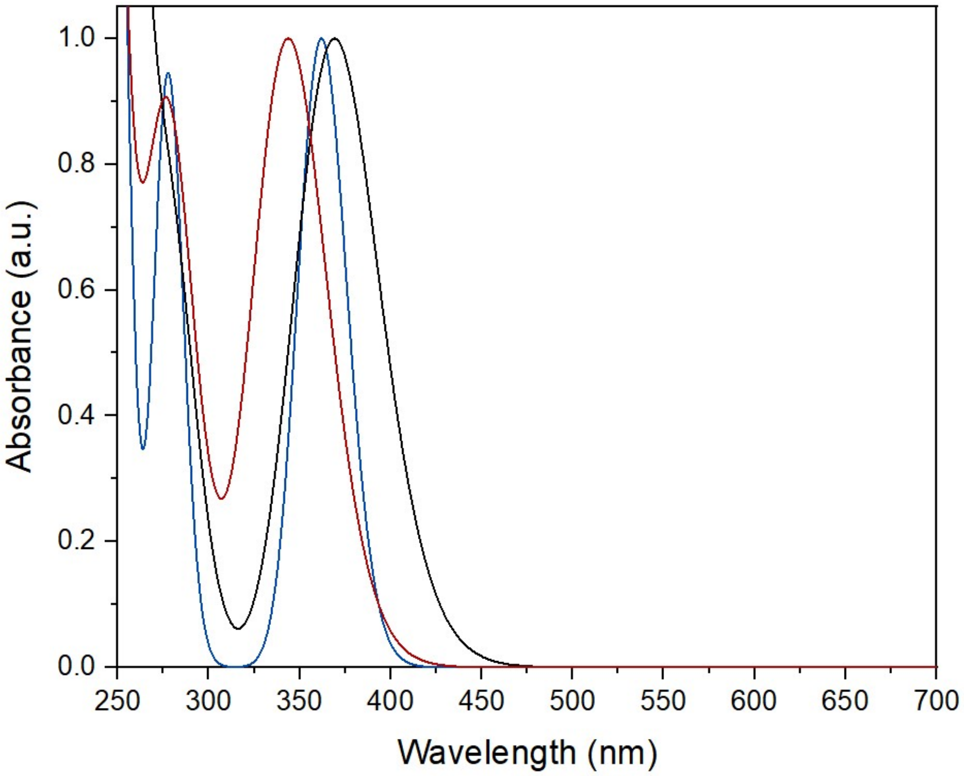

Figure S19 Normalised theoretical absorption spectra of **pH-BTZ** (black) , **pH-BTZ-oPh** (blue) and **pH-BTZ-oPh** (red).

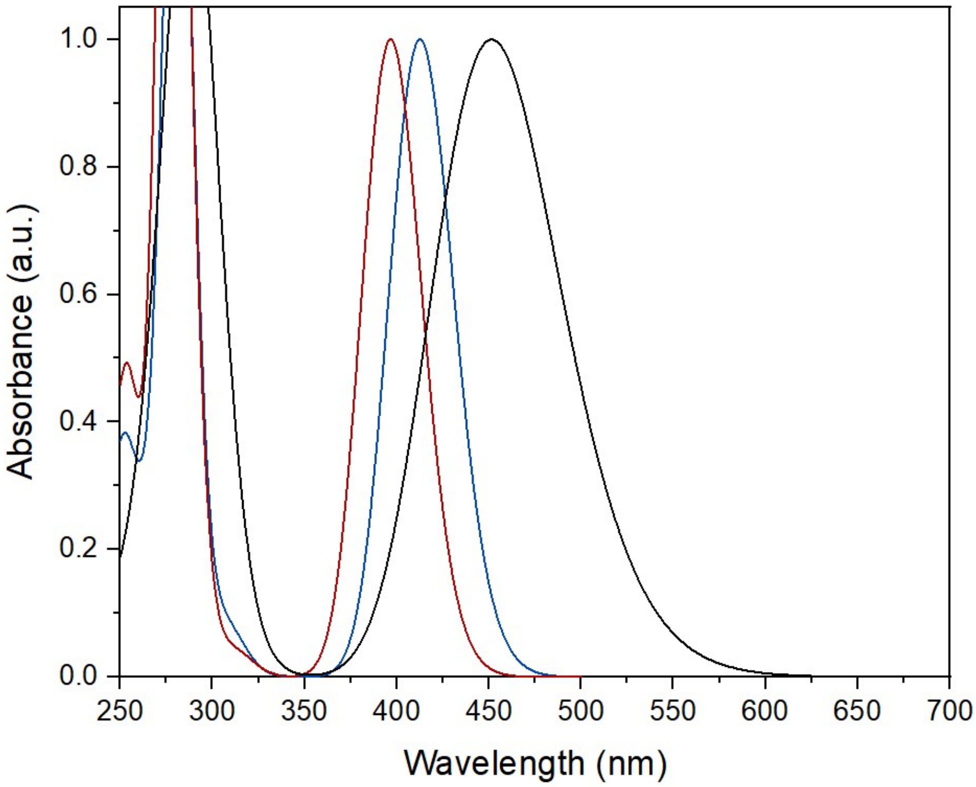

Figure S20 Normalised theoretical absorption spectra of **Th-BTZ** (black) , **Th-BTZ-oPh** (blue) and **Th-BTZ-oPh$_2$** (red).

5.3 Excited State Orbitals

Table S5 Hole and particle natural transition orbitals calculated for **pH-BTZ**.[7]

State	Energy (nm) [contribution]	Hole Orbital	Particle Orbital
**S_1**	371 [0.98888]	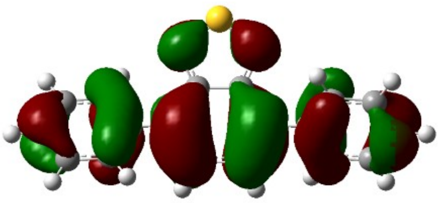	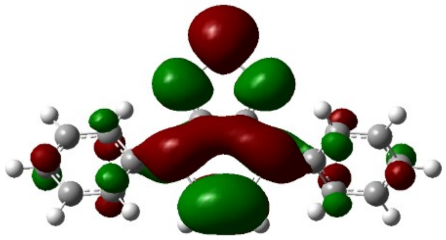
**S_2**	279 [0.90336]	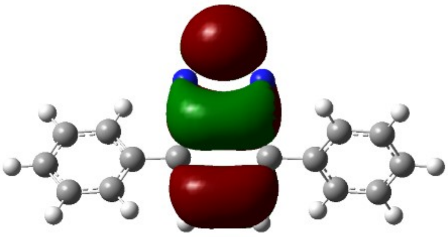	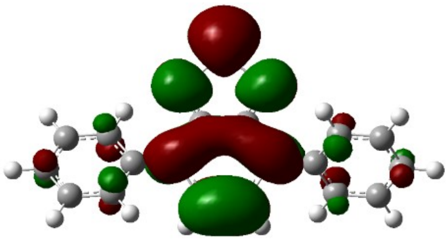
**S_6**	252 [0.63181]	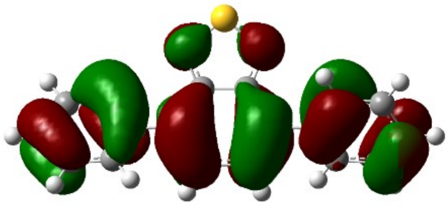	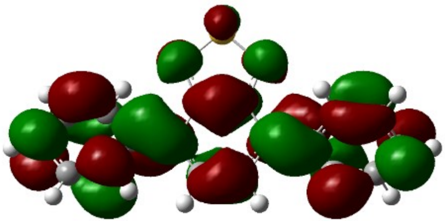

Table S6 Hole and particle natural transition orbitals calculated for **pH-BTZ-oPh**.

State	Energy (nm) [contribution]	Hole Orbital	Particle Orbital
S_1	362 [0.98812]	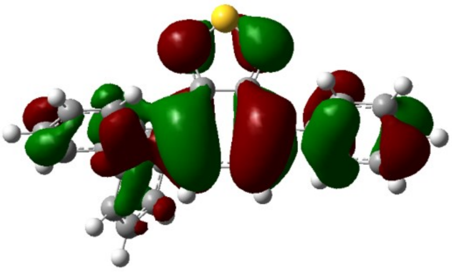	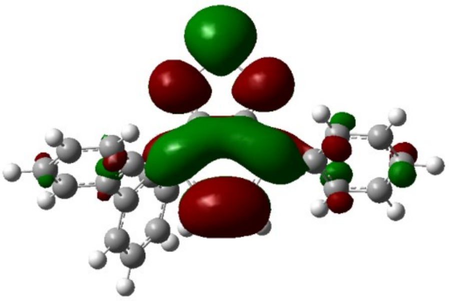
S_2	279 [0.91928]	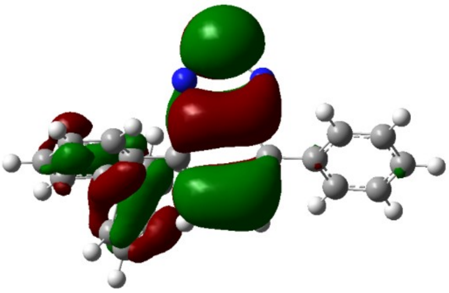	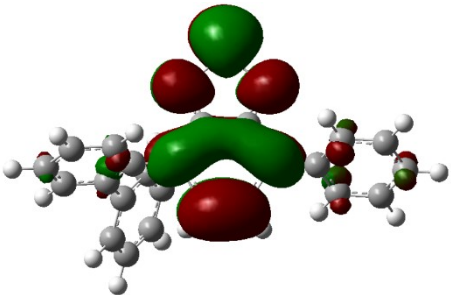
S_6	249 [0.68723]	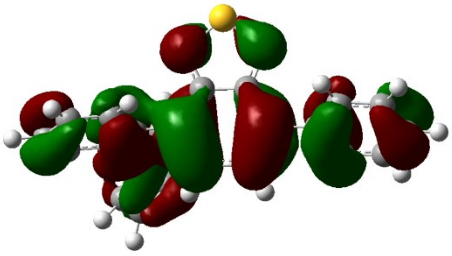	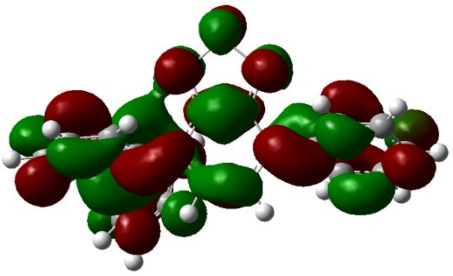

Table S7 Hole and particle natural transition orbitals calculated for **pH-BTZ-oPh$_2$**.[7]

State	Energy (nm)<br>[contribution]	Hole Orbital	Particle Orbital
**S$_1$**	346<br>[0.98655]	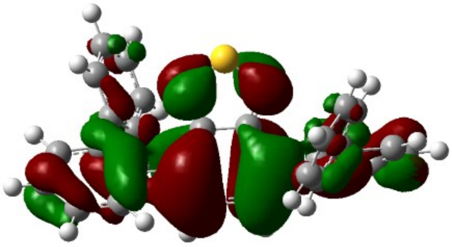	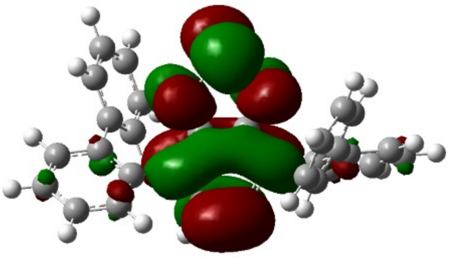
**S$_2$**	287<br>[0.95319]	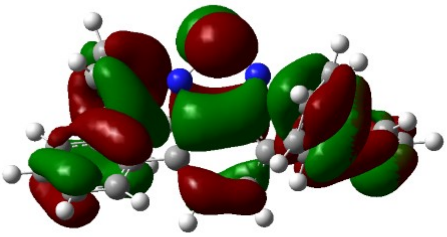	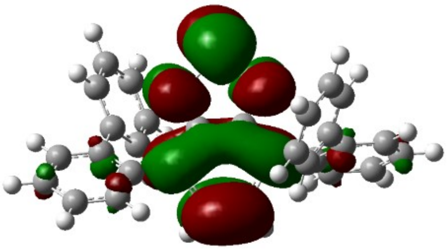
**S$_4$**	275<br>[0.92758]	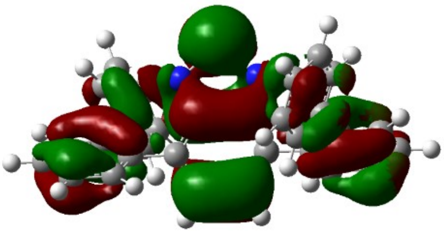	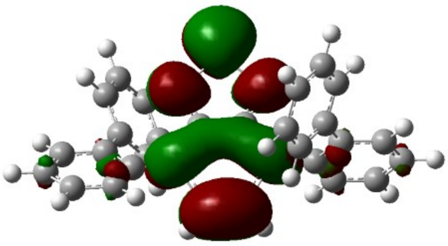

Table S8 Hole and particle natural transition orbitals calculated for **Th-BTZ**.[7]

State	Energy (nm)<br>[contribution]	Hole Orbital	Particle Orbital
**S$_1$**	452<br>[0.98710]	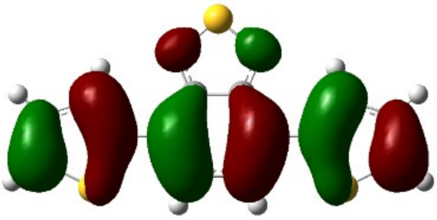	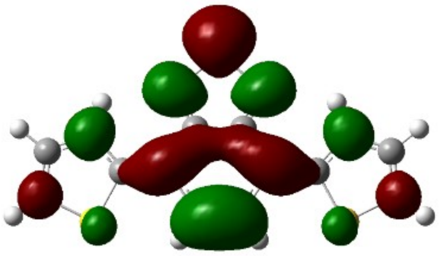
**S$_3$**	288<br>[0.91296]	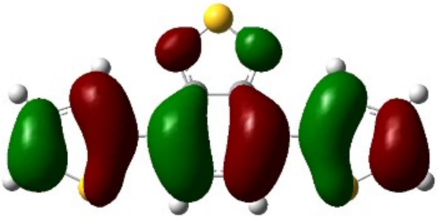	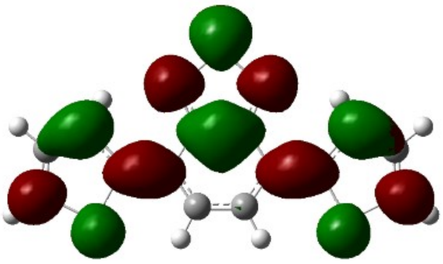

Table S9 Hole and particle natural transition orbitals calculated for **Th-BTZ-oPh**.

State	Energy (nm) [contribution]	Hole Orbital	Particle Orbital
**S_1**	413 [0.98475]	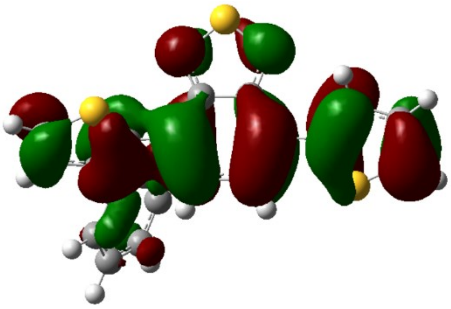	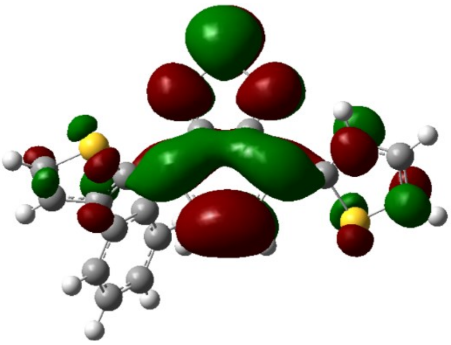
**S_3**	283 [0.85236]	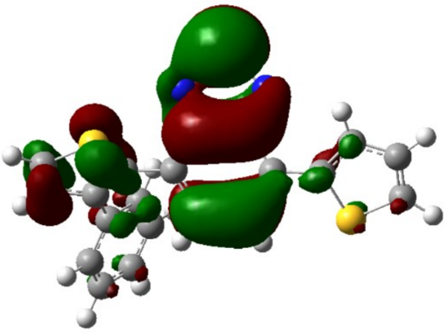	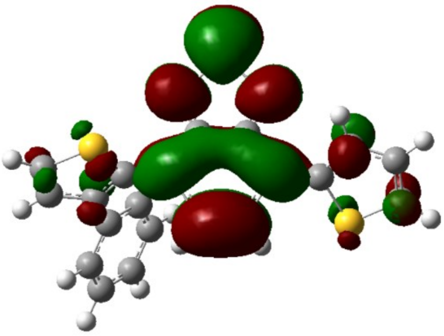
**S_4**	281 [0.69175]	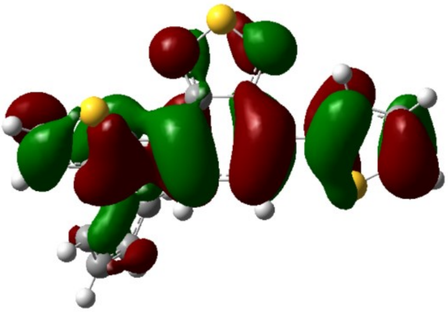	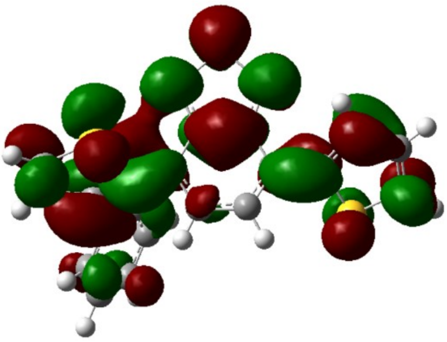
**S_5**	276 [0.79337]	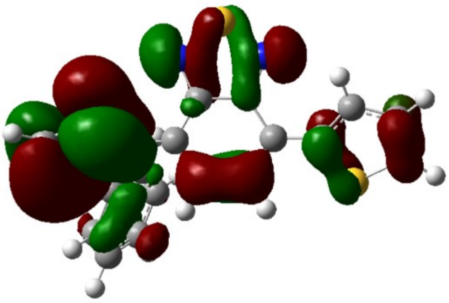	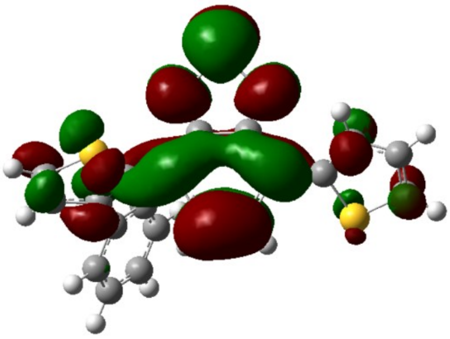

Table S9 (cont) Hole and particle natural transition orbitals calculated for **Th-BTZ-oPh**.

**S_8**	251 [0.49576]	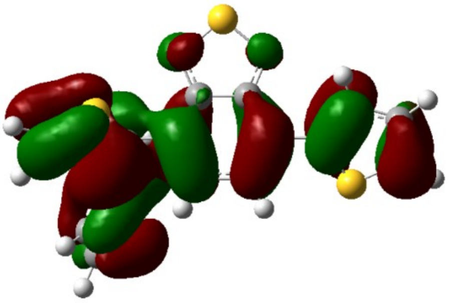 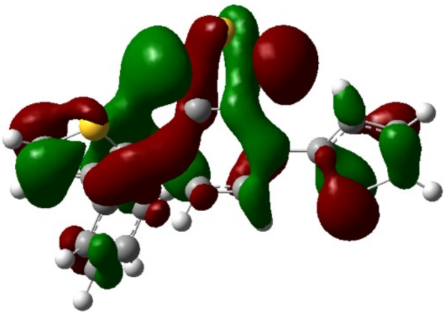	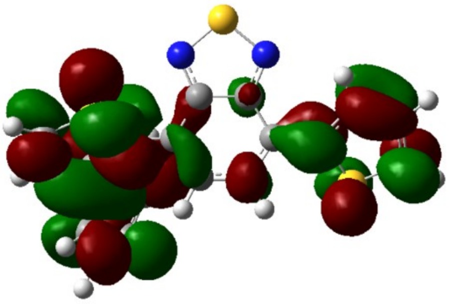 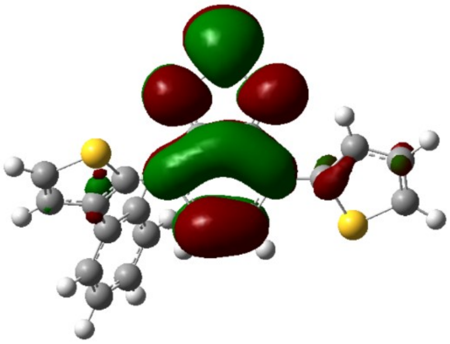

Table S10 Hole and particle natural transition orbitals calculated for **Th-BTZ-oPh$_2$**.

State	Energy (nm) [contribution]	Hole Orbital	Particle Orbital
**S_1**	397 [0.98246]	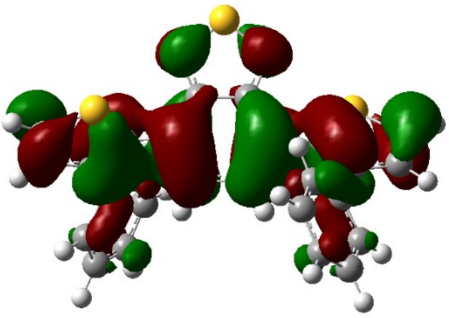	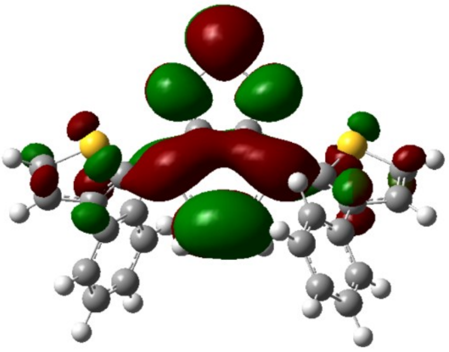
**S_4**	282 [0.90286]	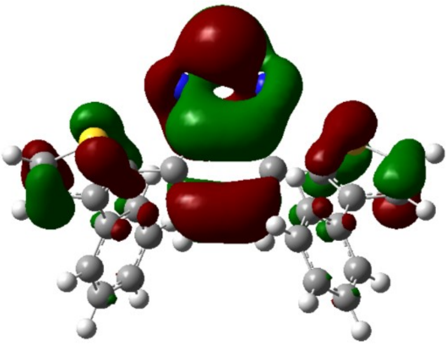	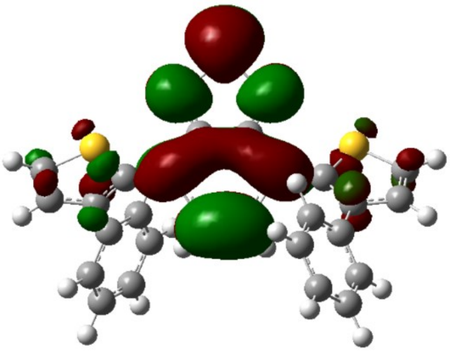
**S_5**	279 [0.93654]	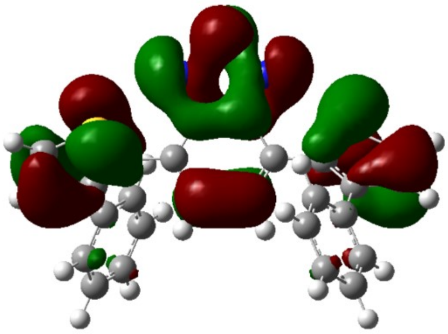	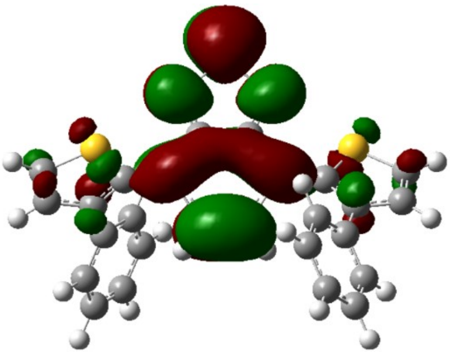
**S_6**	276 [0.7451]	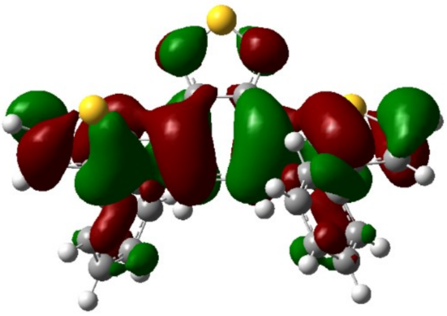	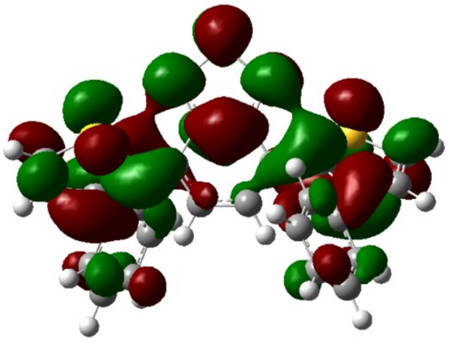

5.4 Energy Barriers to Bond Rotation

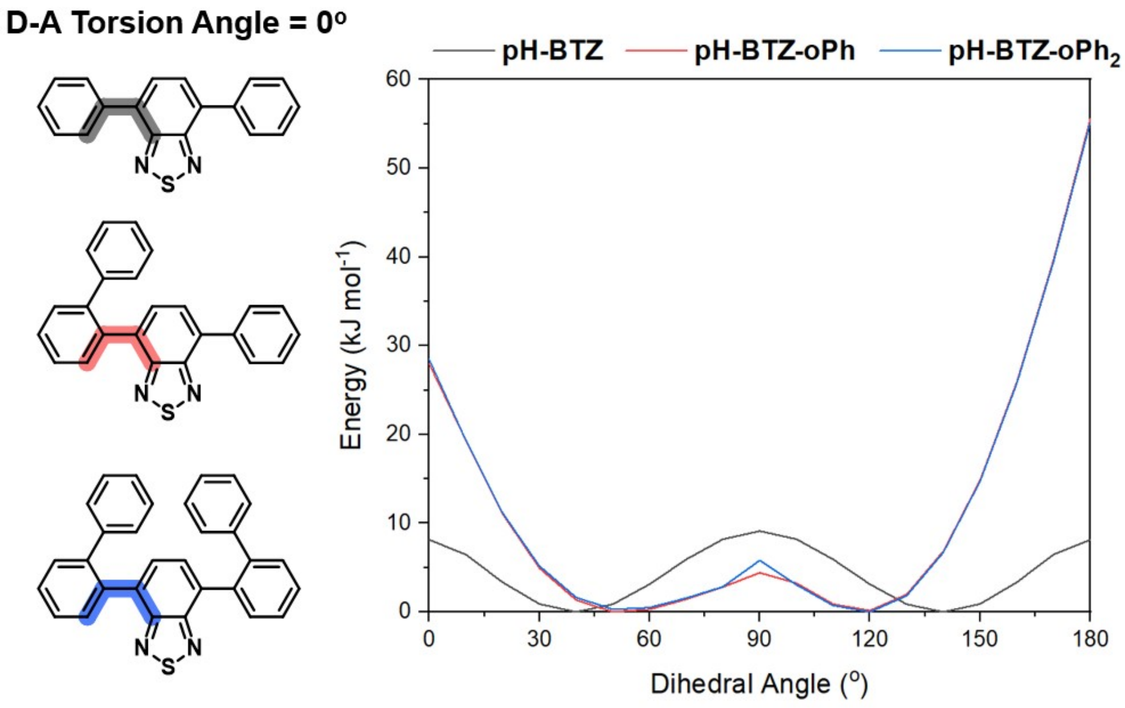

Figure S21 Energy barriers for rotation about the highlighted torsion angle for **pH-BTZ** (black), **pH-BTZ-oPh** (red) and **pH-BTZ-oPh$_2$** (blue).

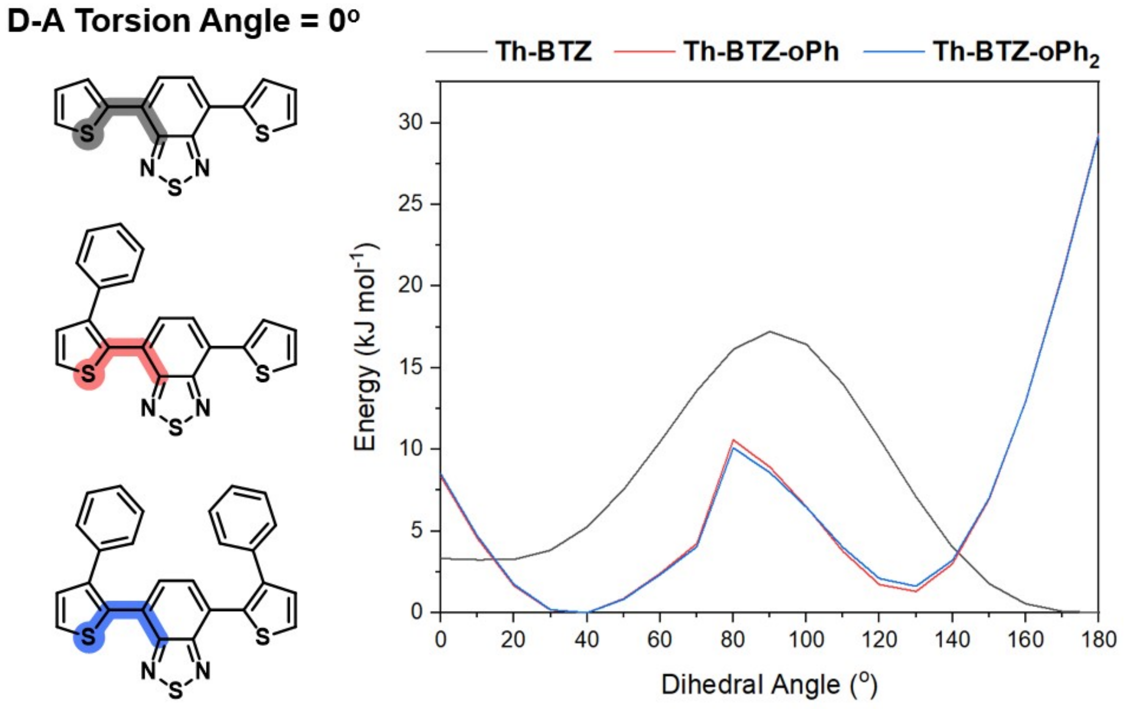

Figure S22 Energy barriers for rotation about the highlighted torsion angle for **Th-BTZ** (black), **Th-BTZ-oPh** (red) and **Th-BTZ-oPh$_2$** (blue).

6. Characterisation of Products

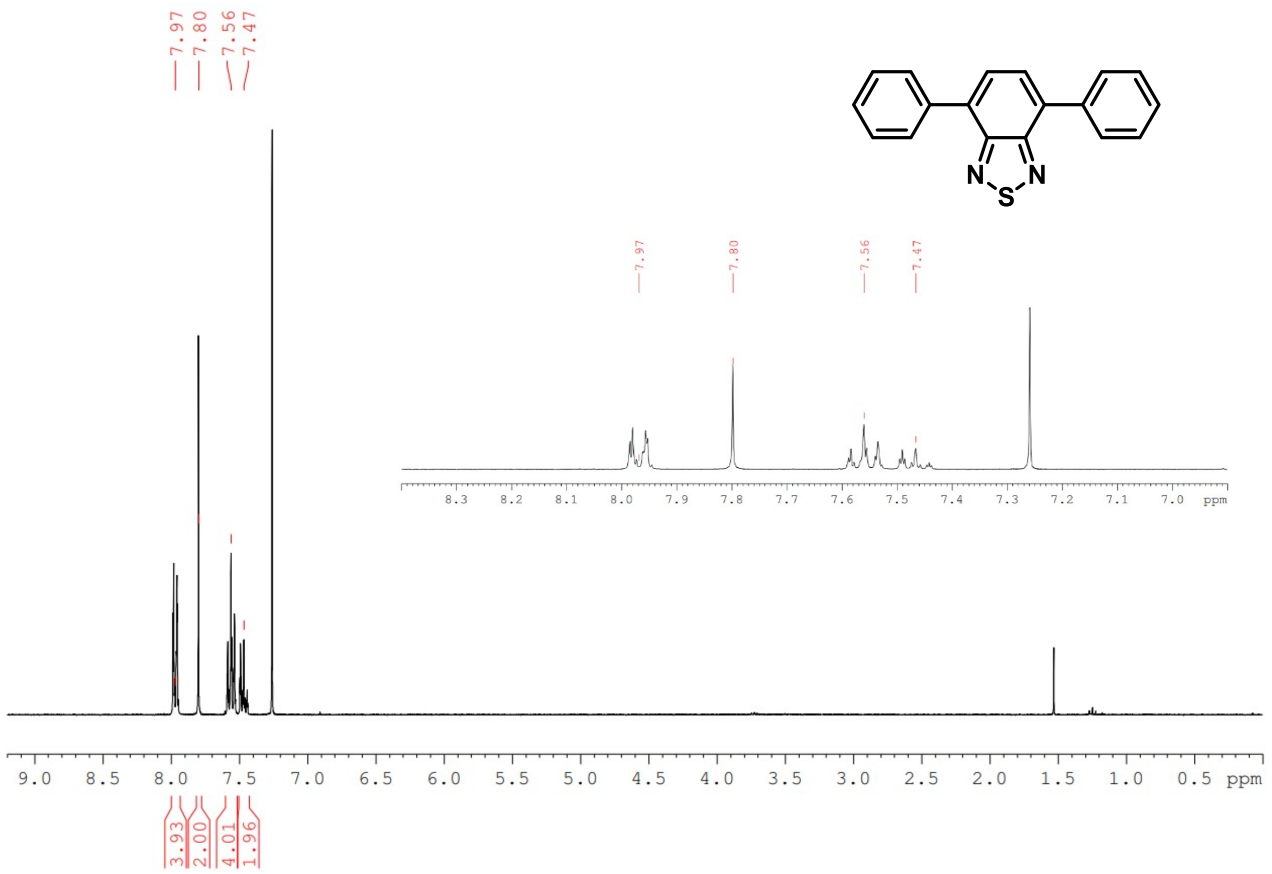

4,7-Diphenylbenzo[*c*][1,2,5]thiadiazole (pH-BTZ)

Figure S23 ^{1}H NMR of **pH-BTZ**.

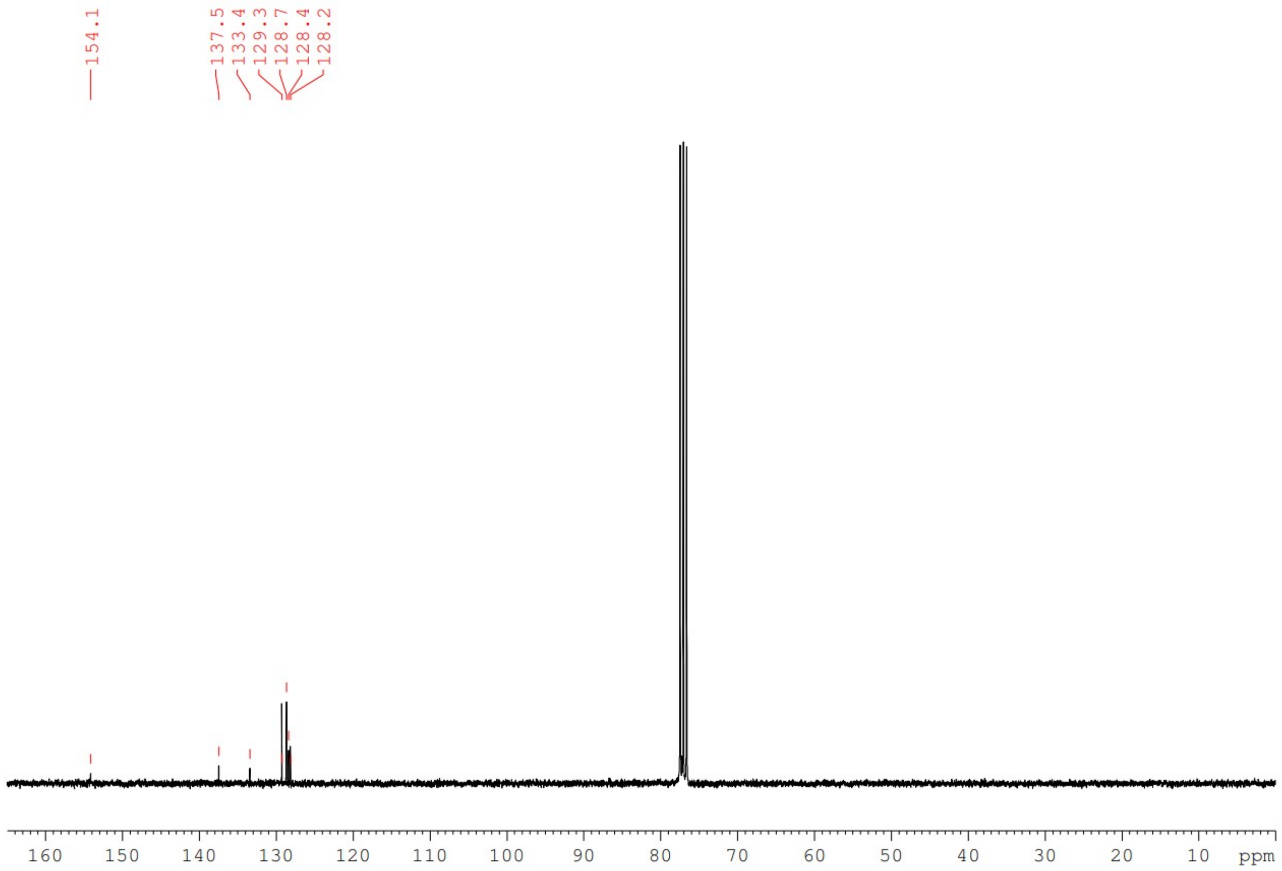

Figure S24 ^{13}C NMR of **pH-BTZ**.

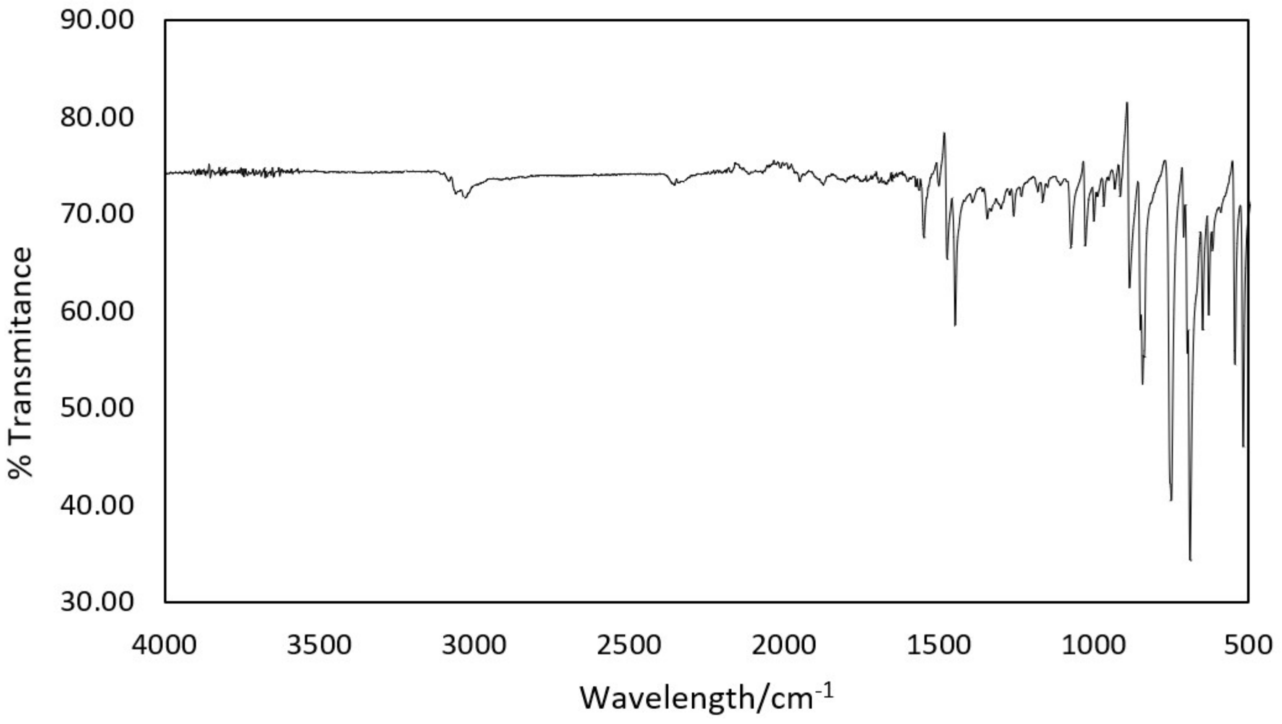

Figure S25 IR spectra of **pH-BTZ**.

pH-BTZ-BCl$_2$

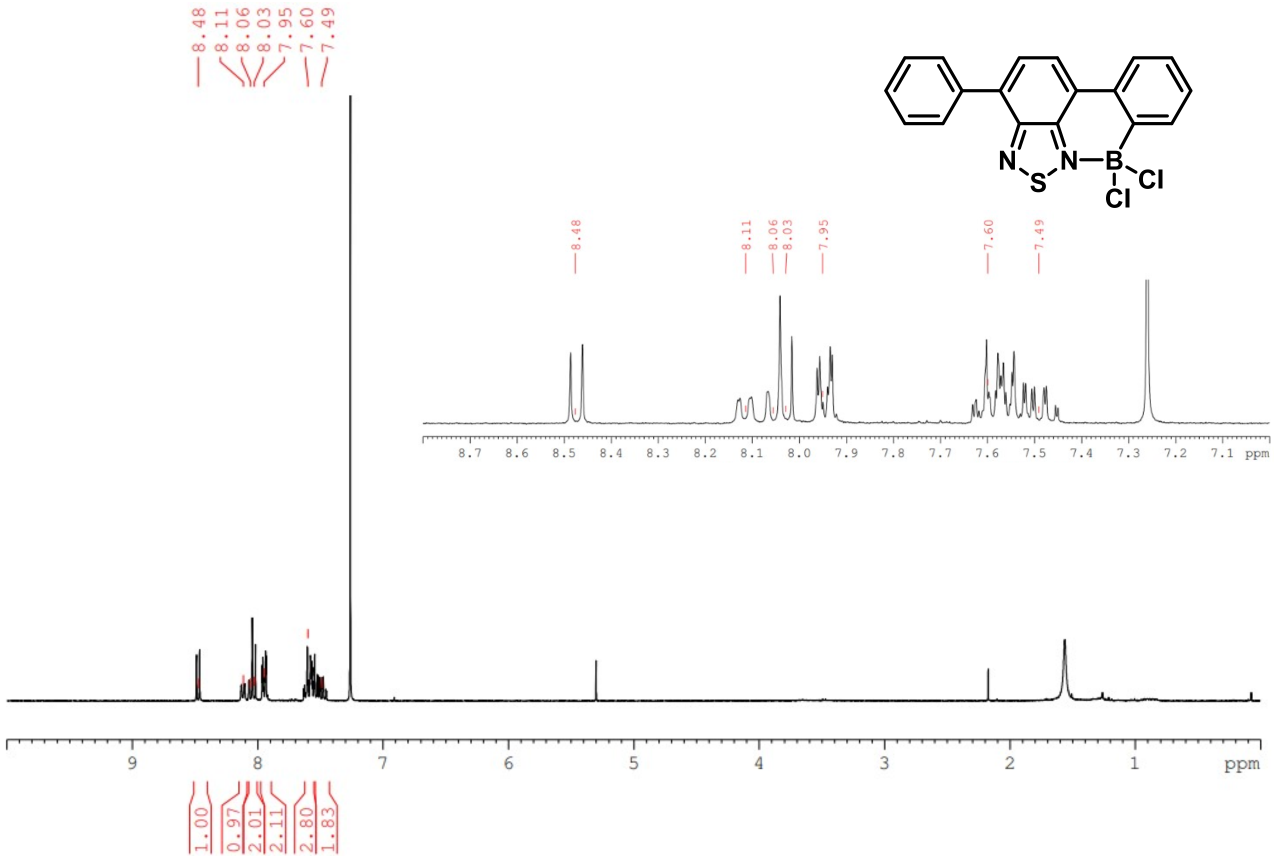

Figure S26 ^{1}H NMR spectra of **pH-BTZ-BCl$_2$**.

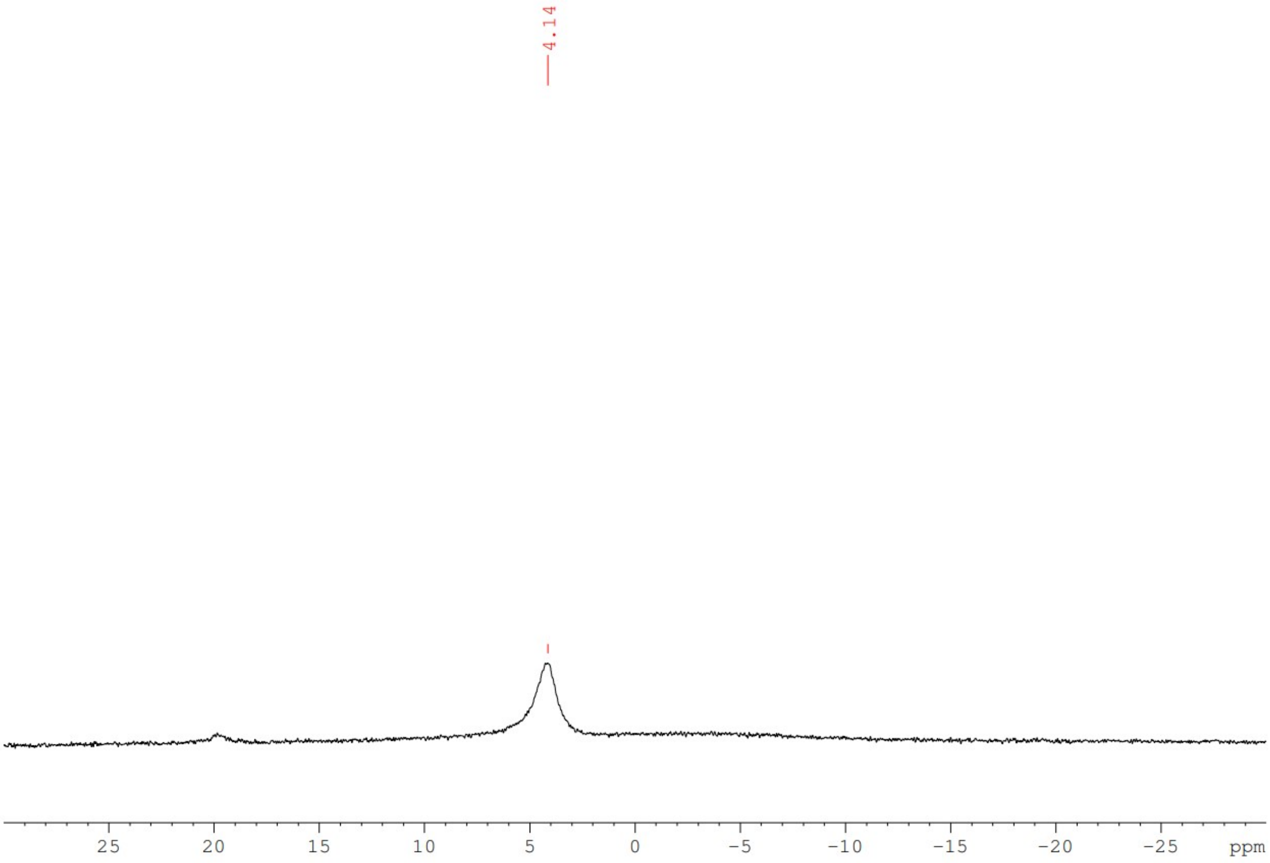

Figure S27 ^{11}B NMR of **pH-BTZ-BCl$_2$**.

4-([1,1'-Biphenyl]-2-yl)-7-phenylbenzo[*c*][1,2,5]thiadiazole (pH-BTZ-oPh)

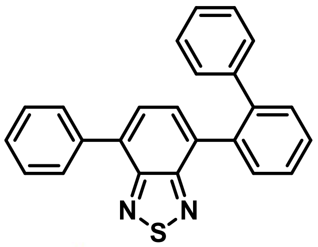

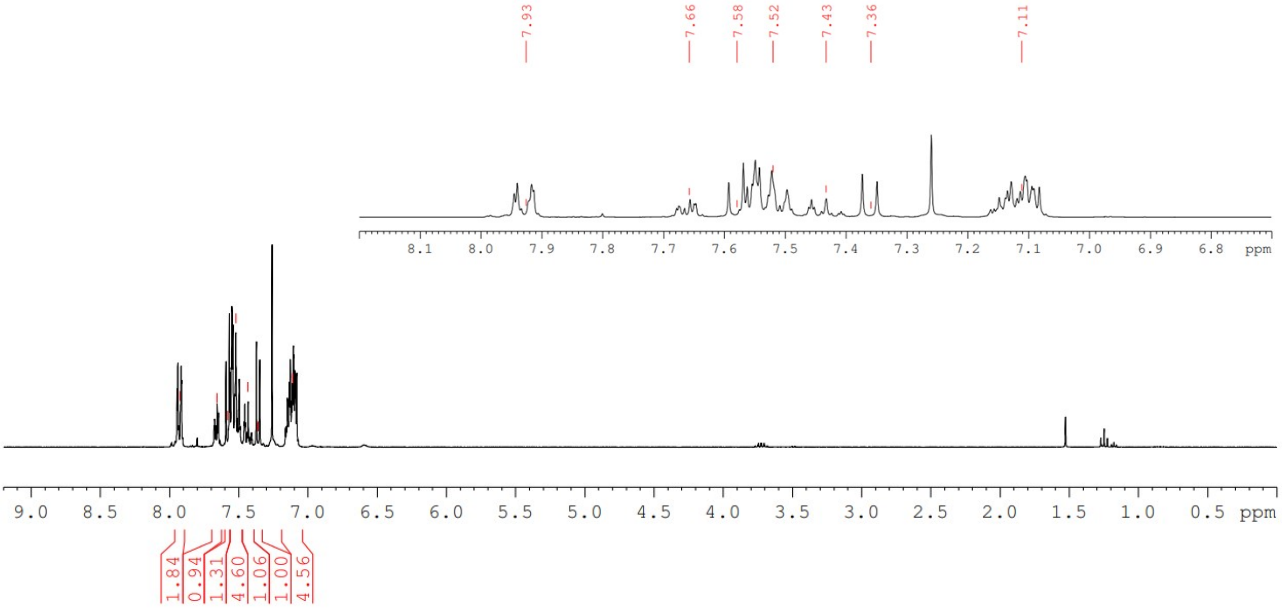

Figure S28 ^{1}H NMR of **pH-BTZ-oPh**.

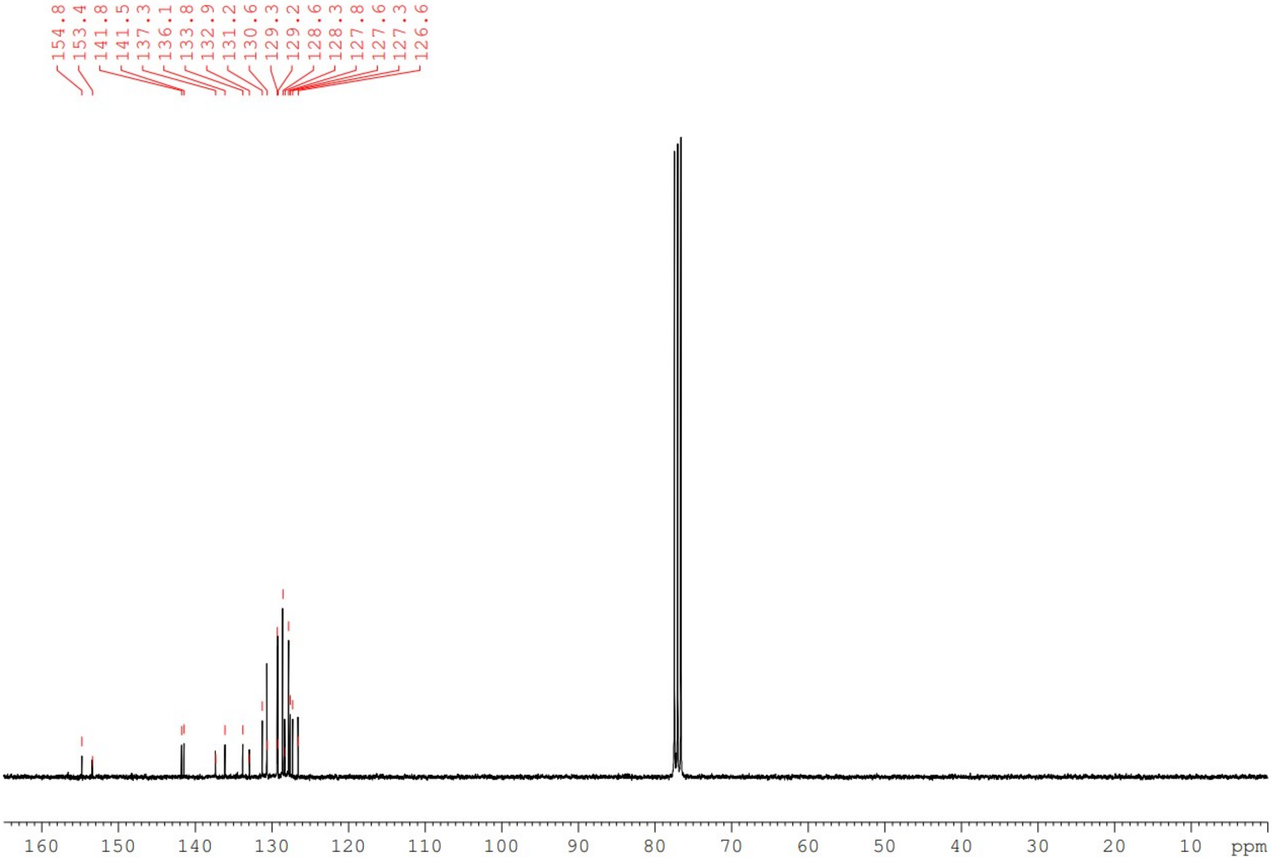

Figure S29 ^{13}C NMR of **pH-BTZ-oPh**.

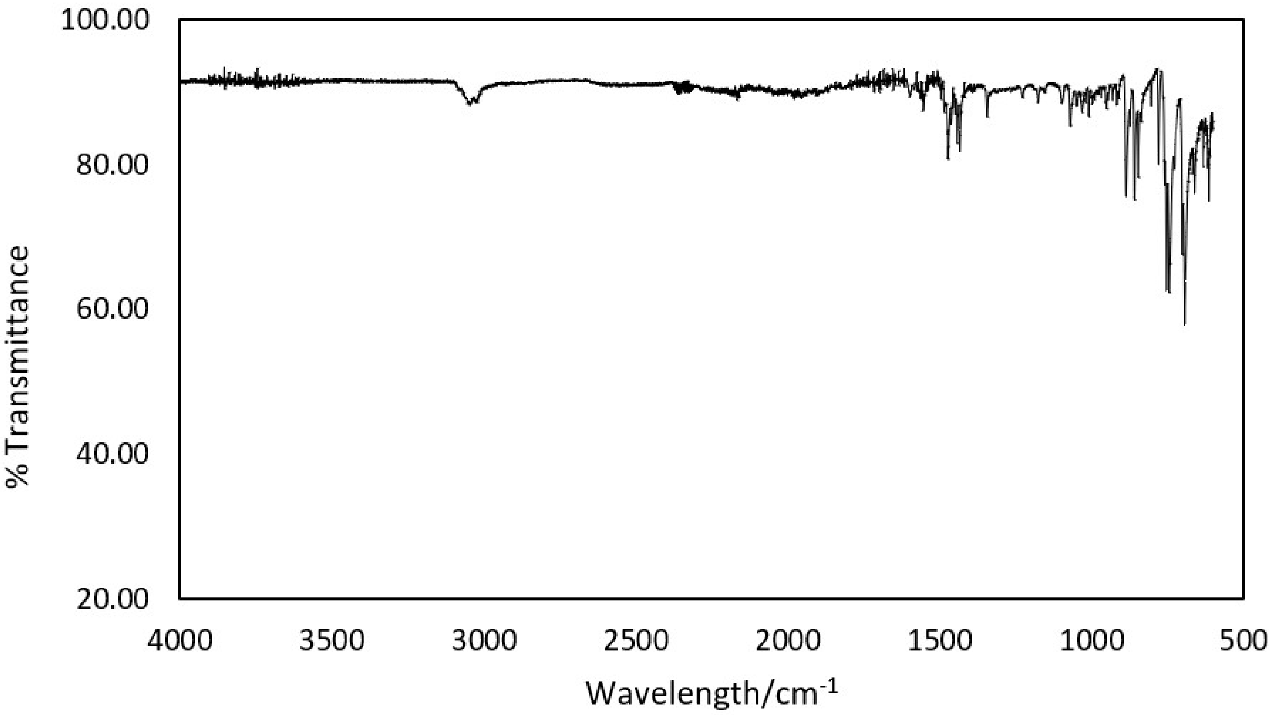

Figure S30 IR spectra of **pH-BTZ-oPh**.

4,7-Di([1,1'-biphenyl]-2-yl)benzo[*c*][1,2,5]thiadiazole (pH-BTZ-oPh$_2$)

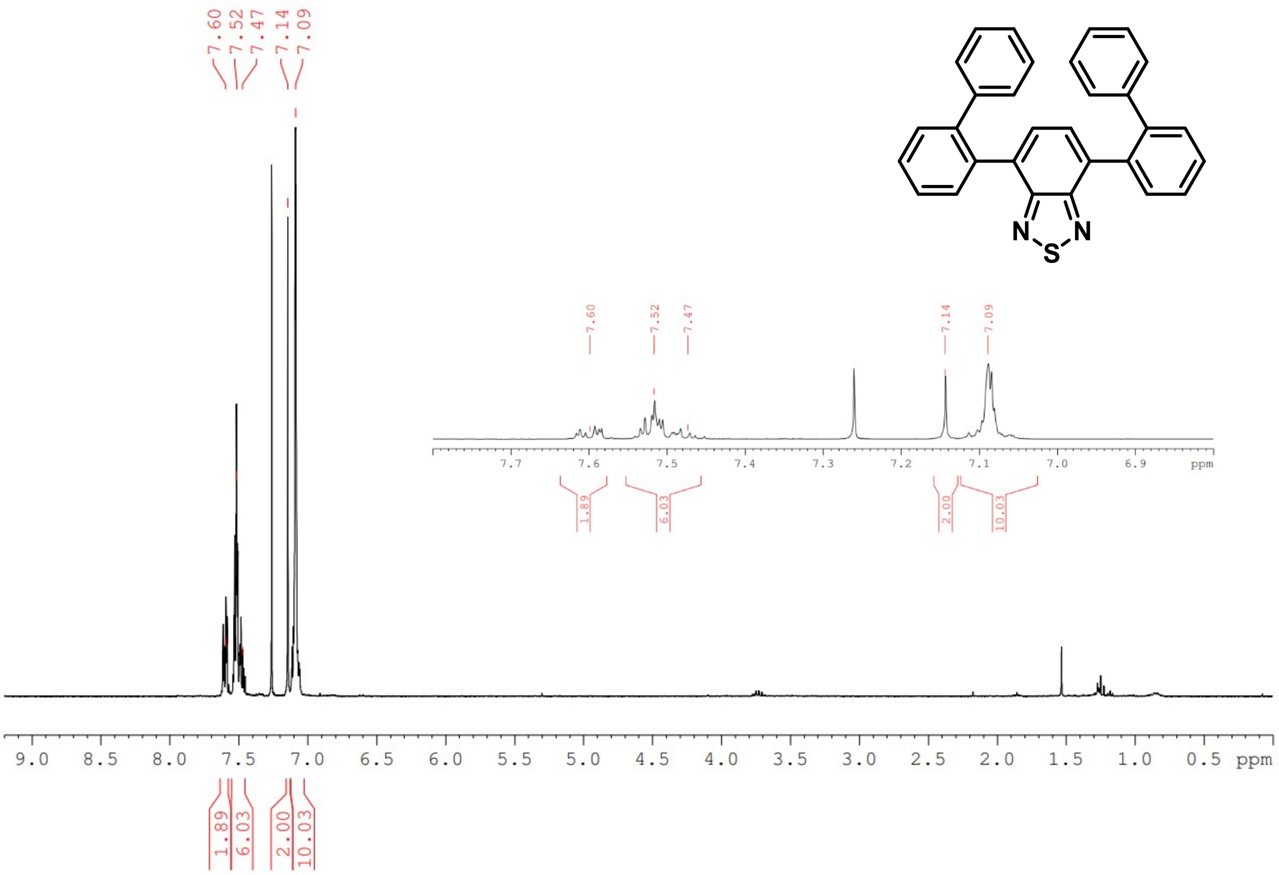

Figure S31 ^{1}H NMR of **pH-BTZ-oPh$_2$**.

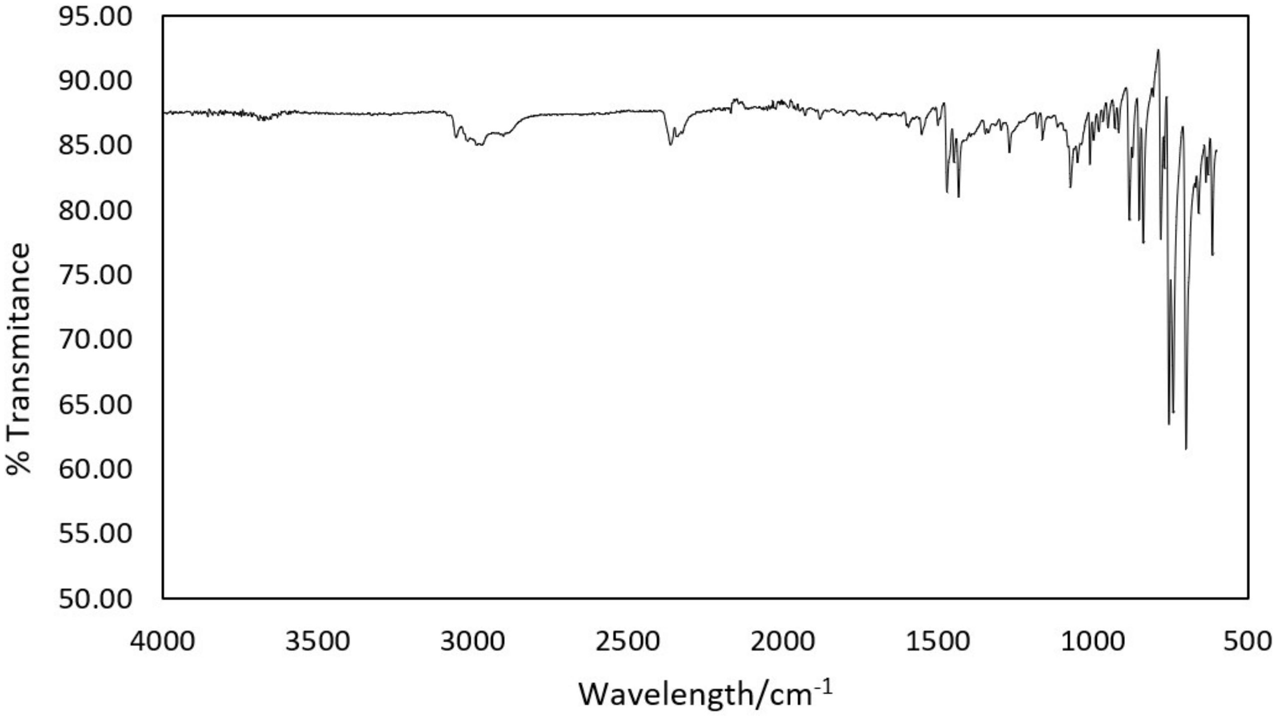

Figure S32 IR spectra of **pH-BTZ-oPh$_2$**.

4,7-Di(thiophen-2-yl)benzo[*c*][1,2,5]thiadiazole (Th-BTZ)

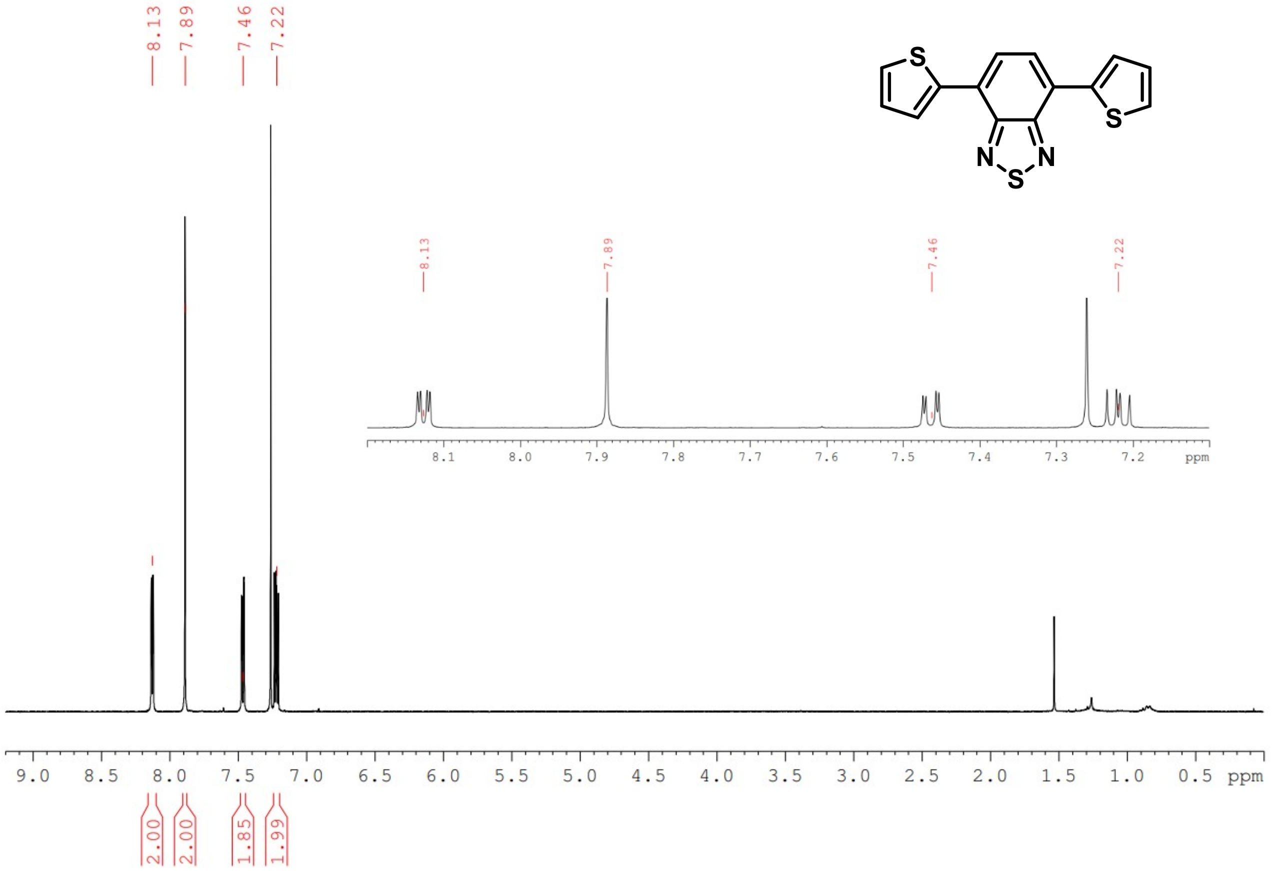

Figure S33 ^{1}H NMR of **Th-BTZ**.

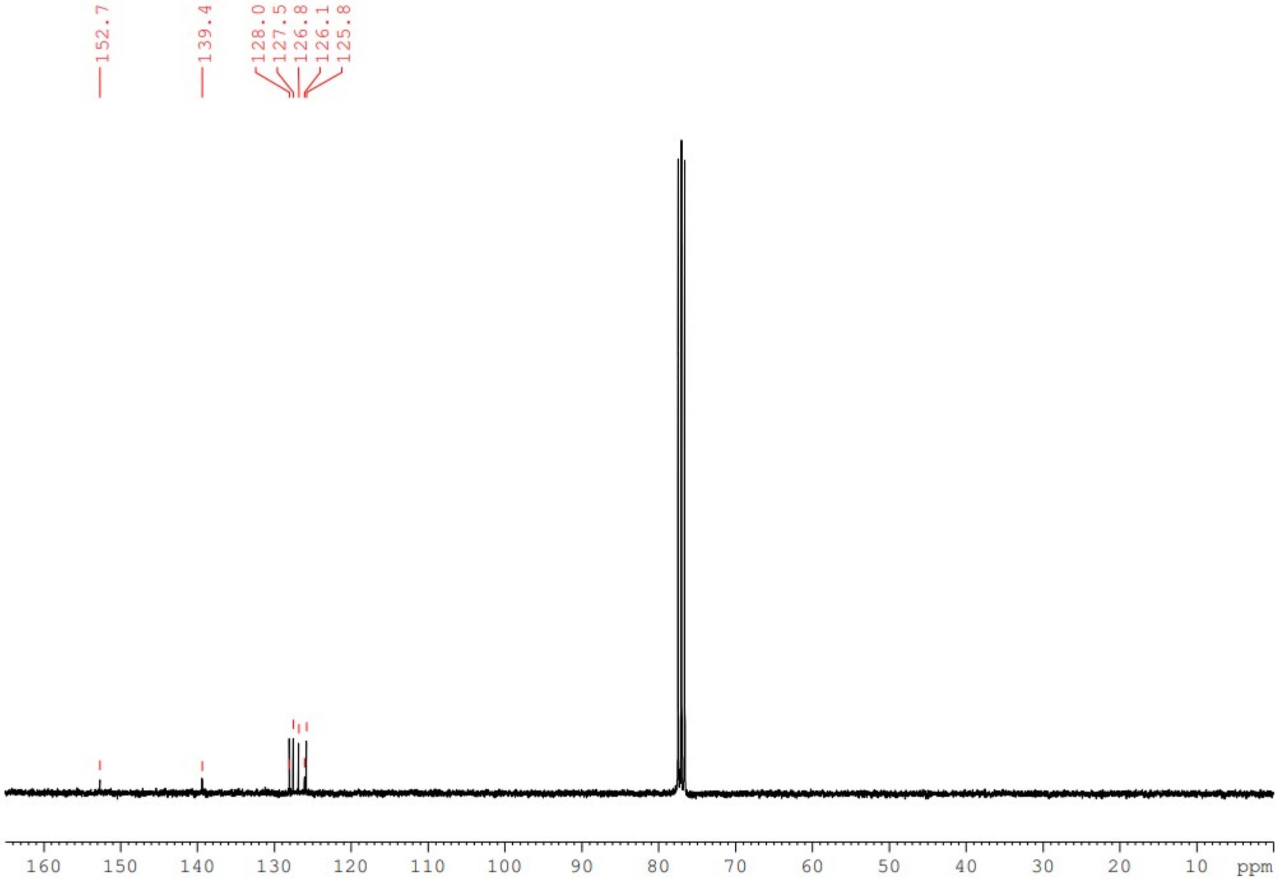

Figure S34 ^{13}C NMR of **Th-BTZ**.

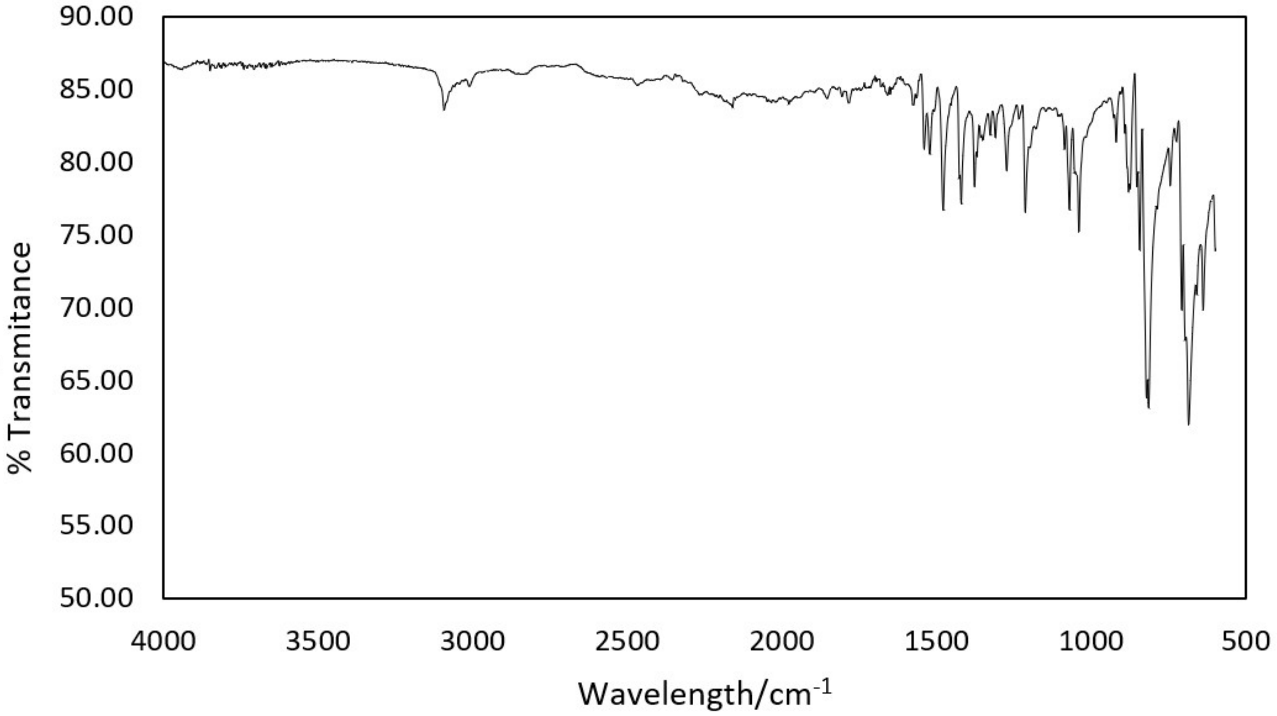

Figure S35 IR spectra of **Th-BTZ**.

Th-BTZ-BCl_2

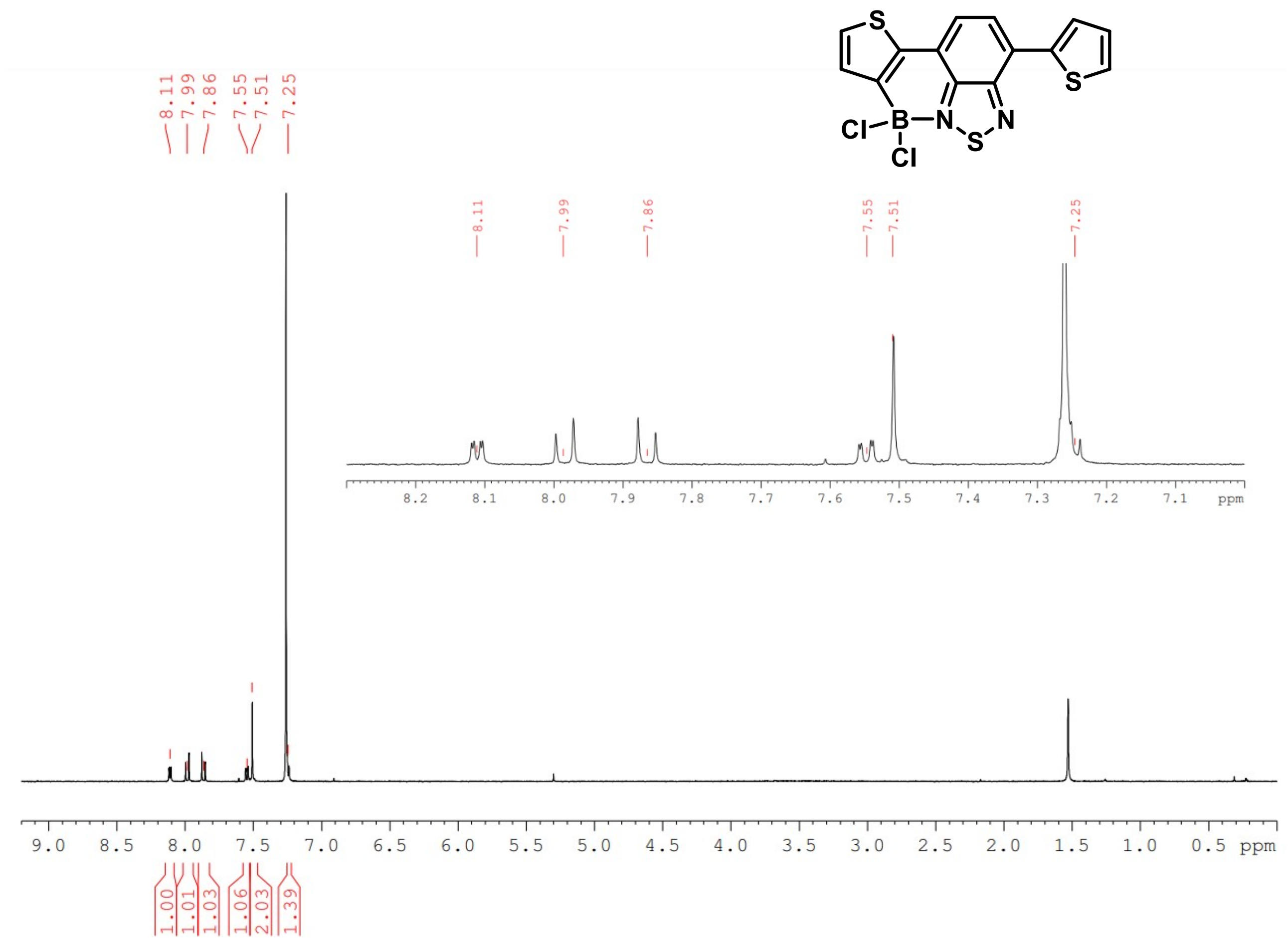

Figure S36 ^{1}H NMR of **Th-BTZ-BCl_2**.

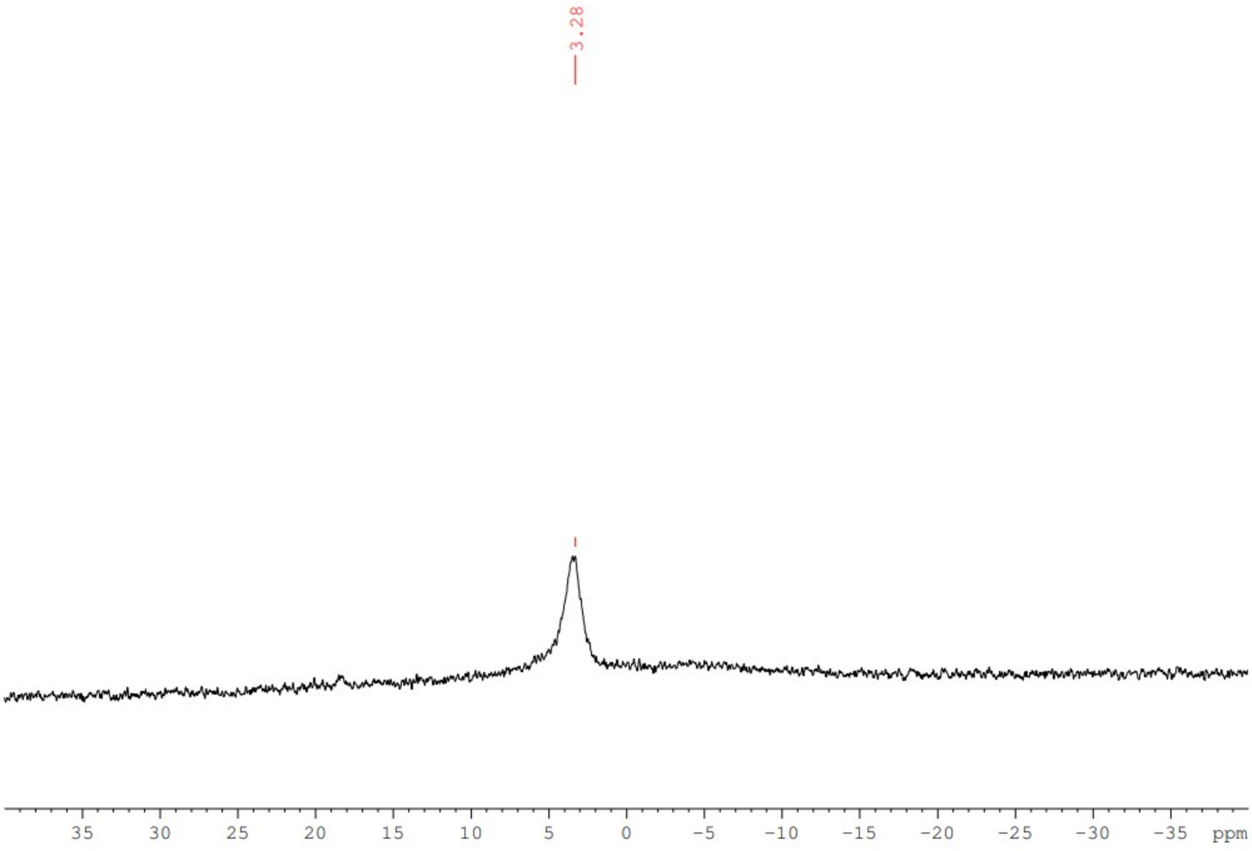

Figure S37 ^{11}B NMR of **Th-BTZ-BCl_2**.

4-(3-Phenylthiophen-2-yl)-7-(thiophen-2-yl)benzo[*c*][1,2,5]thiadiazole (Th-BTZ-oPh)

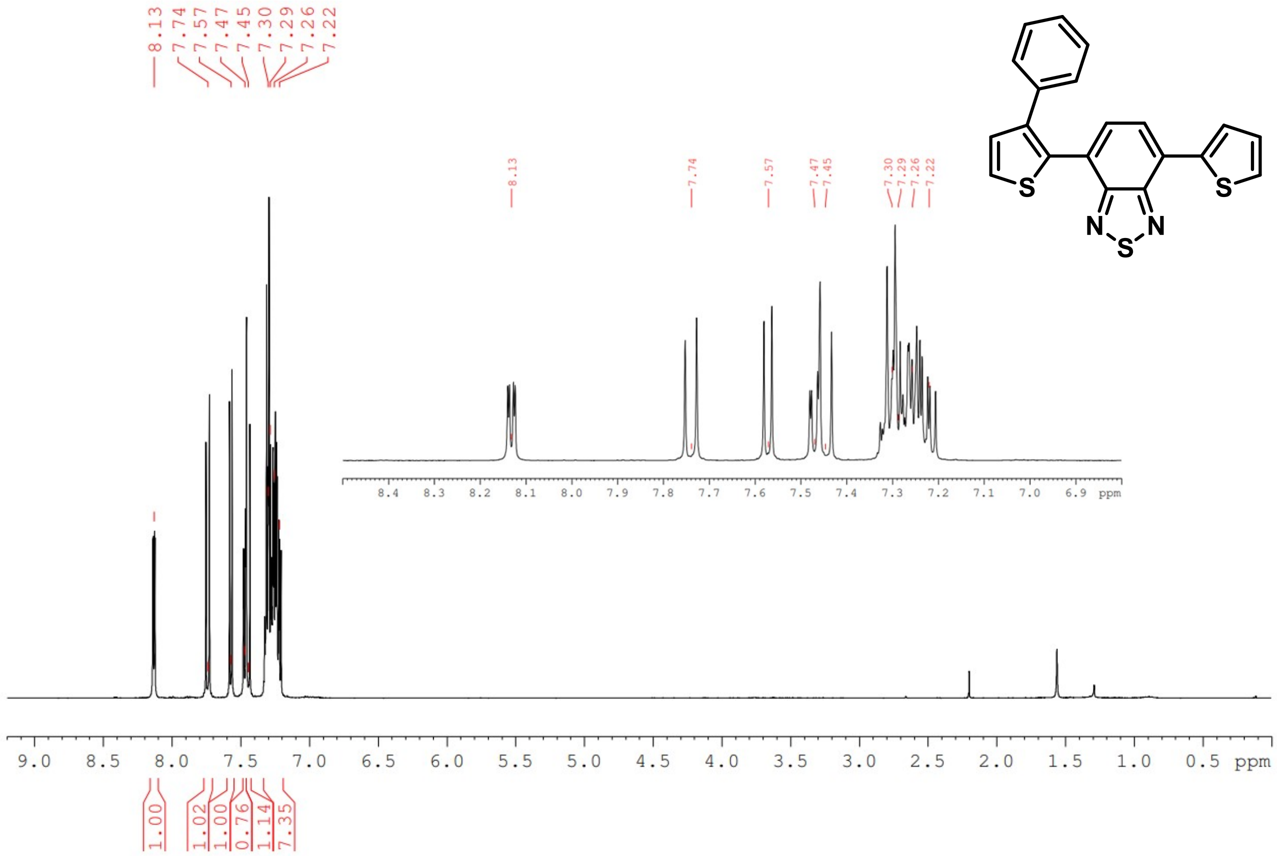

Figure S38 ^{1}H NMR of **Th-BTZ-oPh**.

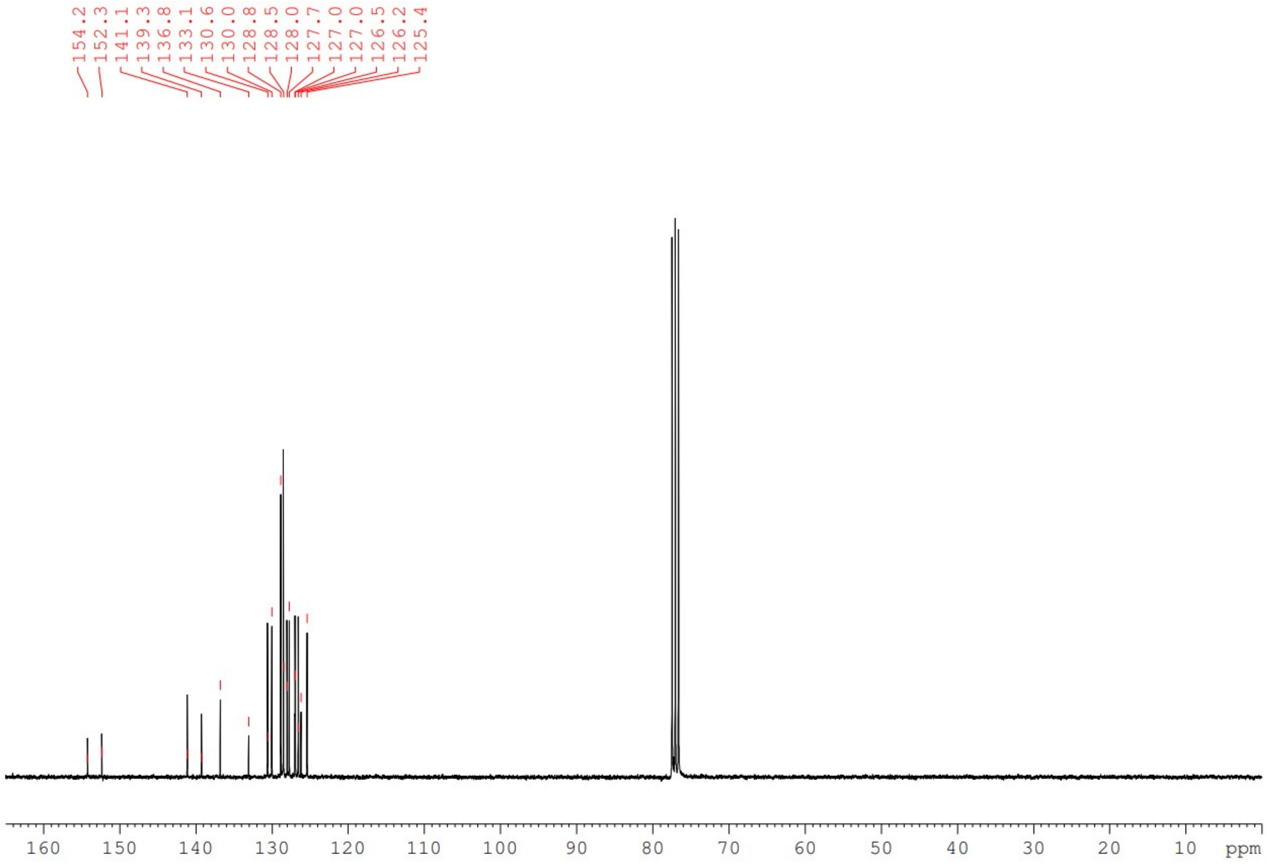

Figure S39 ^{13}C NMR of **Th-BTZ-oPh**.

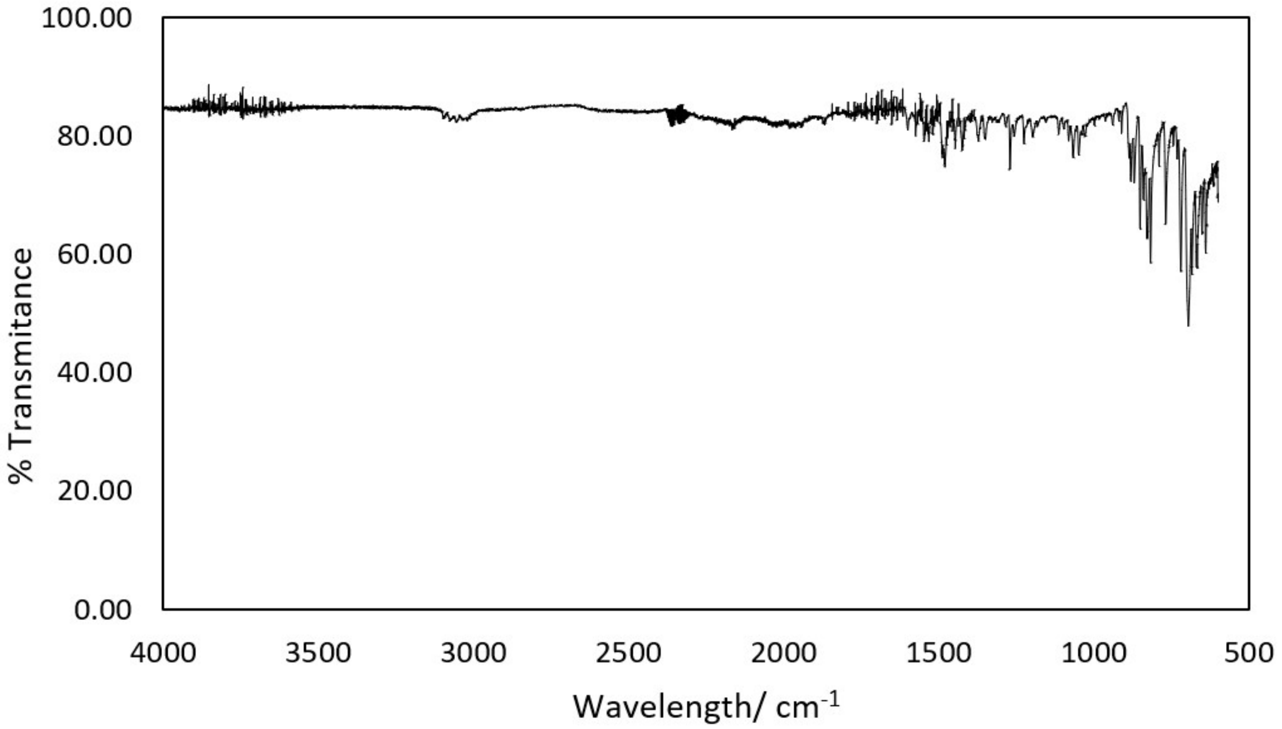

Figure S40 IR spectra of **Th-BTZ-oPh**.

4,7-Bis(3-phenylthiophen-2-yl)benzo[*c*][1,2,5]thiadiazole (ThPh-BTZ-BCl$_2$)

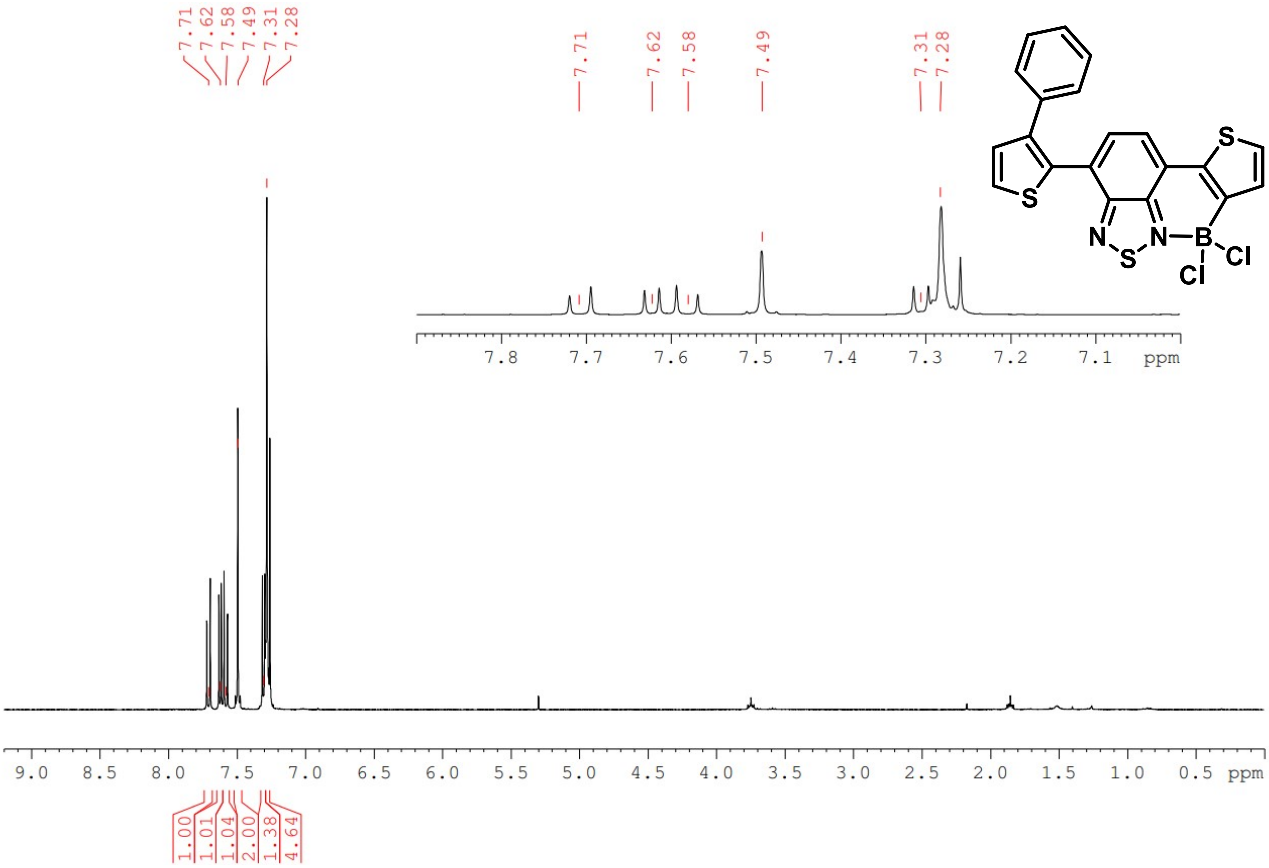

Figure S41 ^{1}H NMR of **ThPh-BTZ-BCl$_2$**

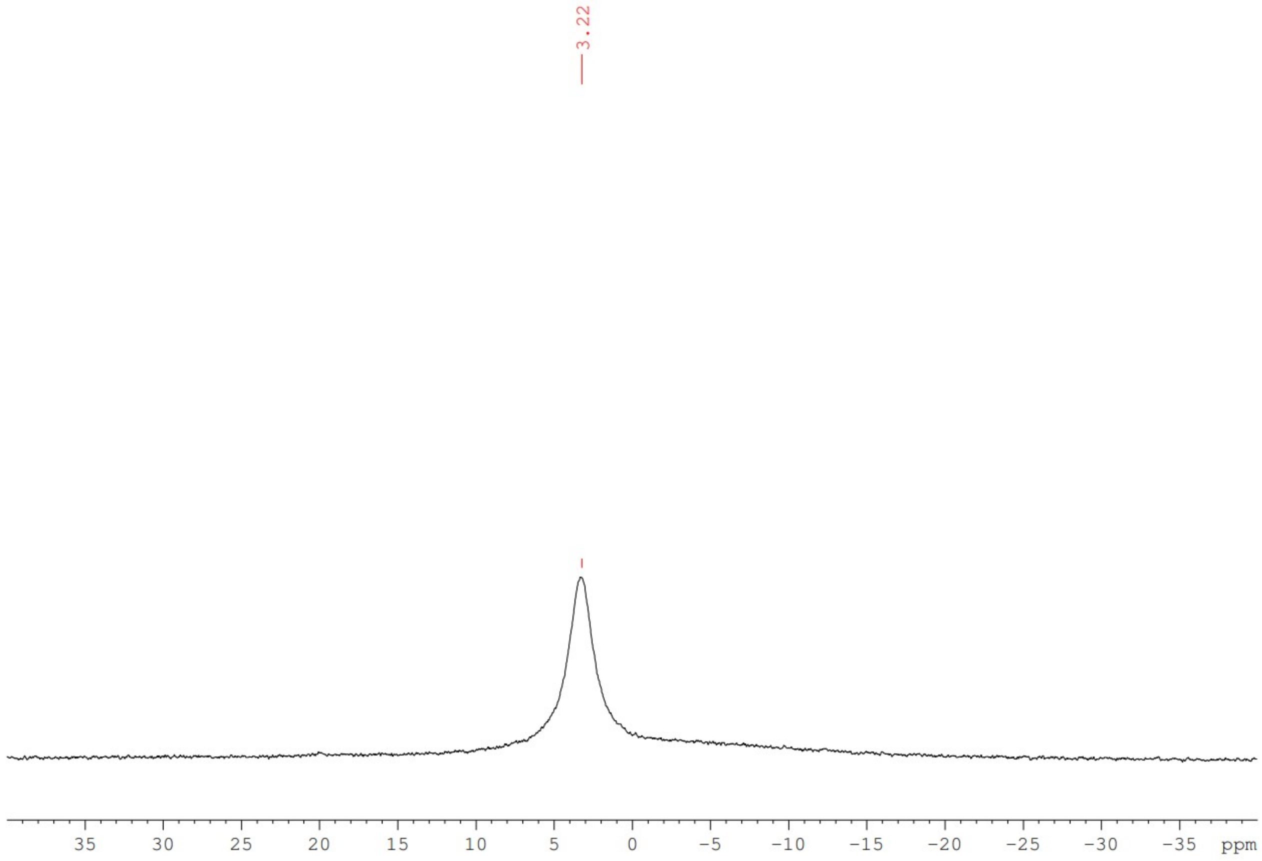

Figure S42 ^{11}B NMR of **ThPh-BTZ-BCl$_2$**

4,7-Bis(3-phenylthiophen-2-yl)benzo[*c*][1,2,5]thiadiazole (Th-BTZ-oPh$_2$)

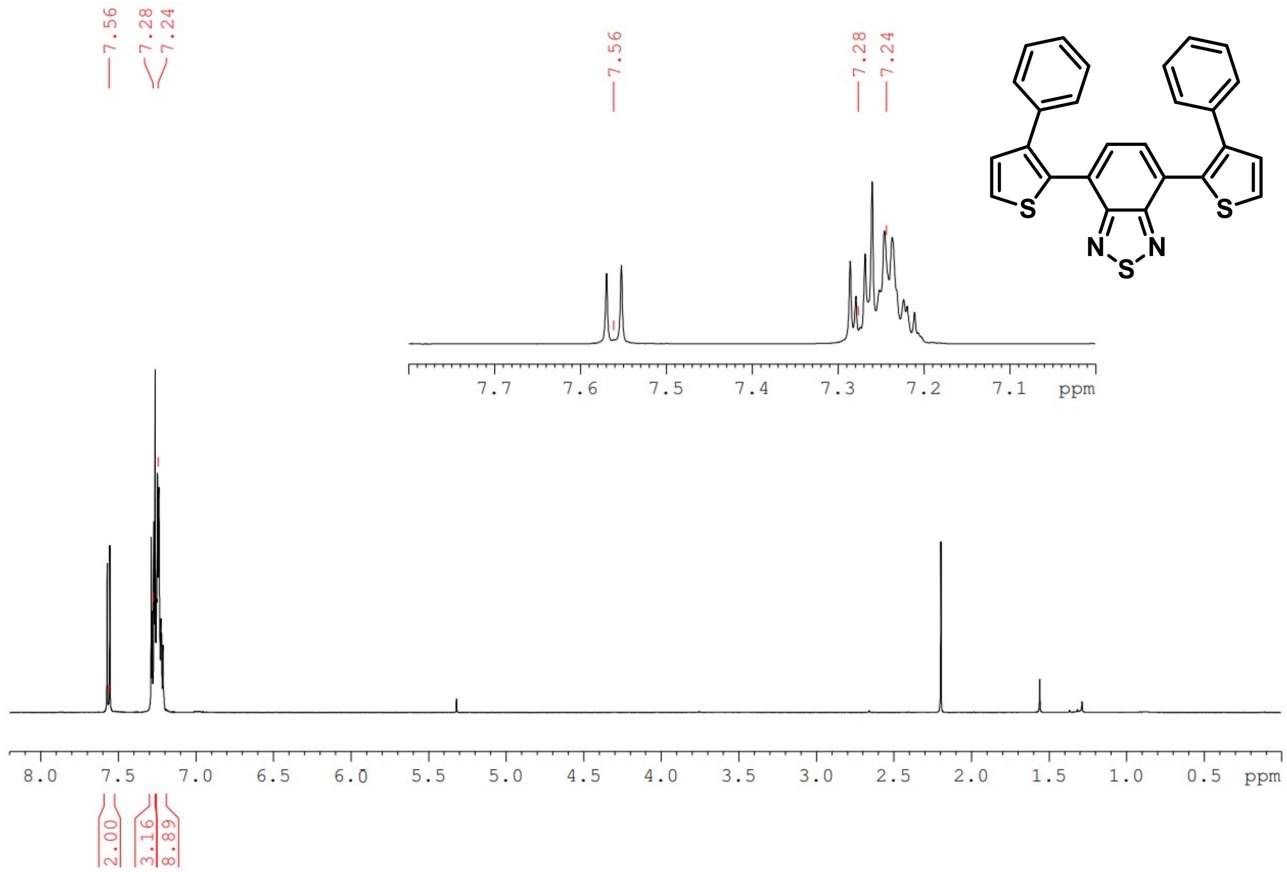

Figure S43 ^{1}H NMR of **Th-BTZ-oPh$_2$**.

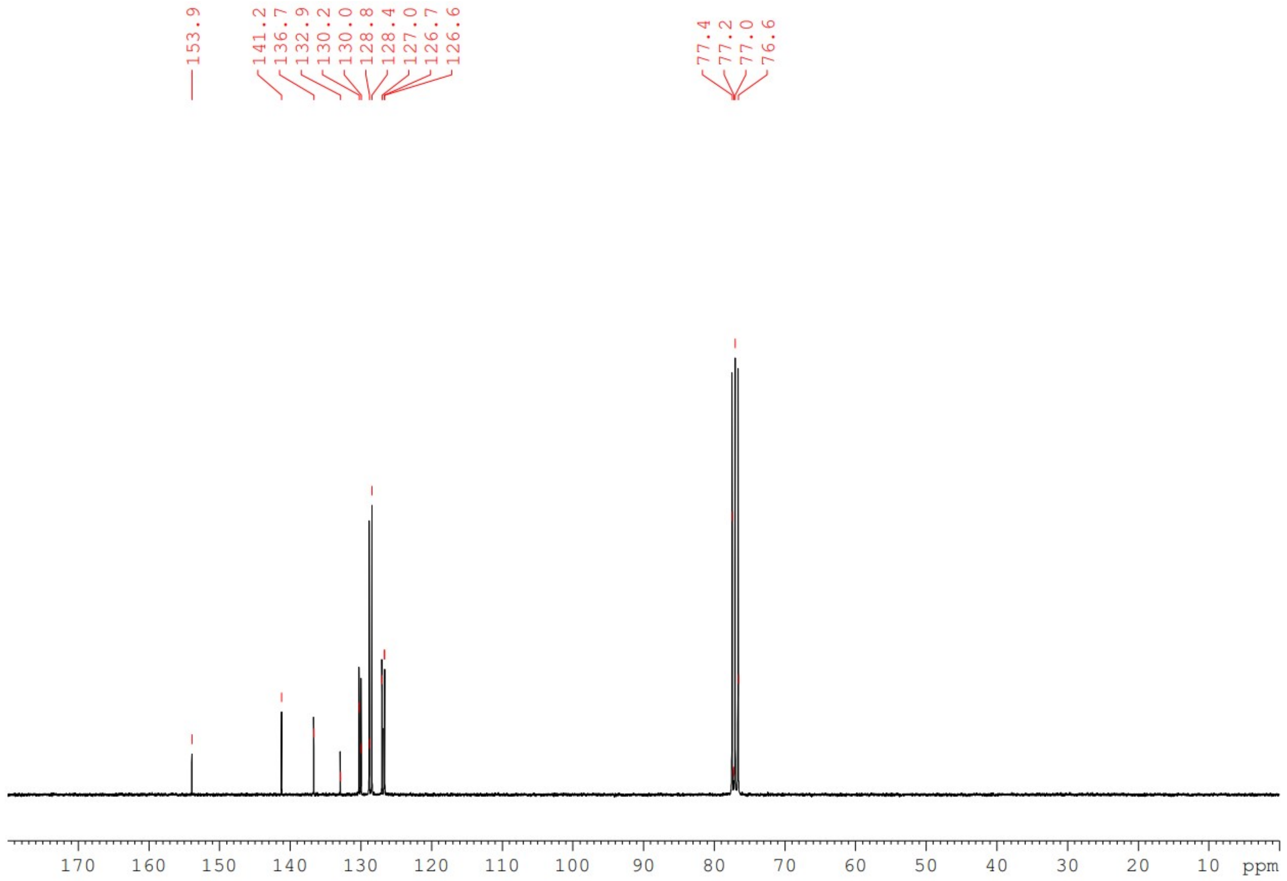

Figure S44 ^{13}C NMR of **Th-BTZ-oPh$_2$**.

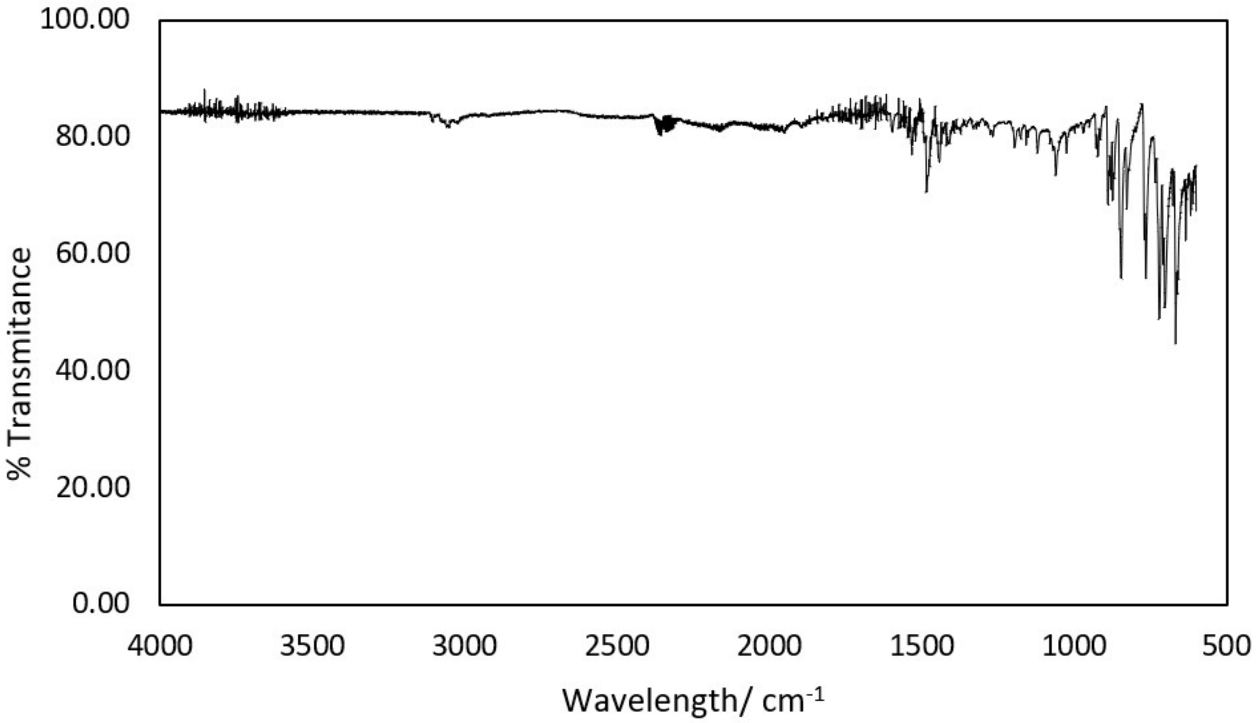

Figure S45 IR spectra of **Th-BTZ-oPh$_2$**.

7. References

1 C. M. S. Jones, A. Gakamsky and J. Marques-Hueso, *Sci. Technol. Adv. Mater.*, 2021, **22**, 810–848.

2 O. V. Dolomanov, L. J. Bourhis, R. J. Gildea, J. A. K. Howard and H. Puschmann, *J. Appl. Crystallogr.*, 2009, **42**, 339–341.

3 G. M. Sheldrick, *Acta Crystallogr. Sect. A Found. Crystallogr.*, 2015, **71**, 3–8.

4 G. M. Sheldrick, *Acta Crystallogr. Sect. C Struct. Chem.,* 2015, **71**, 3–8.

5 M. Ranger, D. Rondeau and M. Leclerc, *Macromolecules*, 1997, **30**, 7686–7691.

6 J. P. Heiskanen, P. Vivo, N. M. Saari, T. I. Hukka, T. Kastinen, K. Kaunisto, H. J. Lemmetyinen and O. E. O. Hormi, *J. Org. Chem.*, 2016, **81**, 1535–1546.

7 D. Taylor, T. Malcomson, A. Zhakeyev, S.-X. Cheng, G. M. Rosair, J. Marques-Hueso, Z. Xu, M. J. Paterson, S. J. Dalgarno and F. Vilela, *Org. Chem. Front.*, 2022, **9**, 5473–5484.

8 S. Dhar, D. K. Rana, S. S. Roy, S. Roy, S. Bhattacharya and S. C. Bhattacharya, *J. Lumin.*, 2012, **132**, 957–964.

9 M. J. Frisch, G. W. Trucks, H. B. Schlegel, G. E. Scuseria, M. A. Robb, J. R. Cheeseman, G. Scalmani, V. Barone, G. A. Petersson, H. Nakatsuji, X. Li, M. Caricato, A. V. Marenich, J. Bloino, B. G. Janesko, R. Gomperts, B. Mennucci, H. P. Hratchian, J. V. Ortiz, A. F. Izmaylov, J. L. Sonnenberg, D. Williams-Young, F. Ding, F. Lipparini, F. Egidi, J. Goings, B. Peng, A. Petrone, T. Henderson, D. Ranasinghe, V. G. Zakrzewski, J. Gao, N. Rega, G. Zheng, W. Liang, M. Hada, M. Ehara, K. Toyota, R. Fukuda, J. Hasegawa, M. Ishida, T. Nakajima, Y. Honda, O. Kitao, H. Nakai, T. Vreven, K. Throssell, J. A. Montgomery, Jr., J. E. Peralta, F. Ogliaro, M. J. Bearpark, J. J. Heyd, E. N. Brothers, K. N. Kudin, V. N. Staroverov, T. A. Keith, R. Kobayashi, J. Normand, K. Raghavachari, A. P. Rendell, J. C. Burant, S. S. Iyengar, J. Tomasi, M. Cossi, J. M. Millam, M. Klene, C. Adamo, R. Cammi, J. W. Ochterski, R. L. Martin, K. Morokuma, O. Farkas, J. B. Foresman, and D. J. Fox, Gaussian 16, Revision A.03, Inc., Wallingford CT, 2016.

10 R. Dennington, T. A. Keith and J. M. Millam, GaussView Version 6, Semichem Inc., Shawnee Mission KS, 2016.

11 B. Miehlich, A. Savin, H. Stoll and H. Preuss, *Chem. Phys. Lett.*, 1989, **157**, 200–206.

12 C. Lee, W. Yang and R. G. Parr, *Phys. Rev. B*, 1988, **37**, 785–789.

13 A. D. Becke, *J. Chem. Phys.*, 1993, **98**, 1372–1377.

14 A. D. Becke, *Phys. Rev. A*, 1988, **38**, 3098–3100.

15 R. Krishnan, J. S. Binkley, R. Seeger and J. A. Pople, *J. Chem. Phys.*, 1980, **72**, 650–654.

16 T. H. Dunning, *J. Chem. Phys.*, 1989, **90**, 1007–1023.

17 A. V. Marenich, C. J. Cramer and D. G. Truhlar, *J. Phys. Chem. B*, 2009, **113**, 6378–6396.

18 T. Yanai, D. P. Tew and N. C. Handy, *Chem. Phys. Lett.*, 2004, **393**, 51–57.

19 T. Malcomson and M. J. Paterson, *Photochem. Photobiol. Sci.*, 2020, **19**, 1538–1547.

20 M. J. Paterson, O. Christiansen, F. Pawłowski, P. Jørgensen, C. Hättig, T. Helgaker and P. Sałek, *J. Chem. Phys.*, 2006, **124**, 054322.

21 J. Arnbjerg, M. J. Paterson, C. B. Nielsen, M. Jørgensen, O. Christiansen and P. R. Ogilby, *J. Phys. Chem. A*, 2007, **111**, 5756–5767.

22 M. Johnsen, M. J. Paterson, J. Arnbjerg, O. Christiansen, C. B. Nielsen, M. Jørgensen and P. R. Ogilby, *Phys. Chem. Chem. Phys.*, 2008, **10**, 1177–1191.

23 L. Therese Bergendahl and M. J. Paterson, *Chem. Commun.*, 2012, **48**, 1544–1546.

24 S. I. Kato, T. Matsumoto, M. Shigeiwa, H. Gorohmaru, S. Maeda, T. Ishi-i and S. Mataka, *Chem. - A Eur. J.*, 2006, **12**, 2303–2317.

25 C. B. Nielsen, A. J. P. White and I. McCulloch, *J. Org. Chem.,* 2015, **80**, 5045–5048.
